# Supplementary figures and images for: STAT3 promotes RNA polymerase III-directed transcription by controlling the miR-106a-5p/TP73 axis (part 1 of 2)
Source: eLife. 2023 Jan 19;12:e82826. doi: 10.7554/eLife.82826 (PMC9851613; doi:10.7554/eLife.82826)

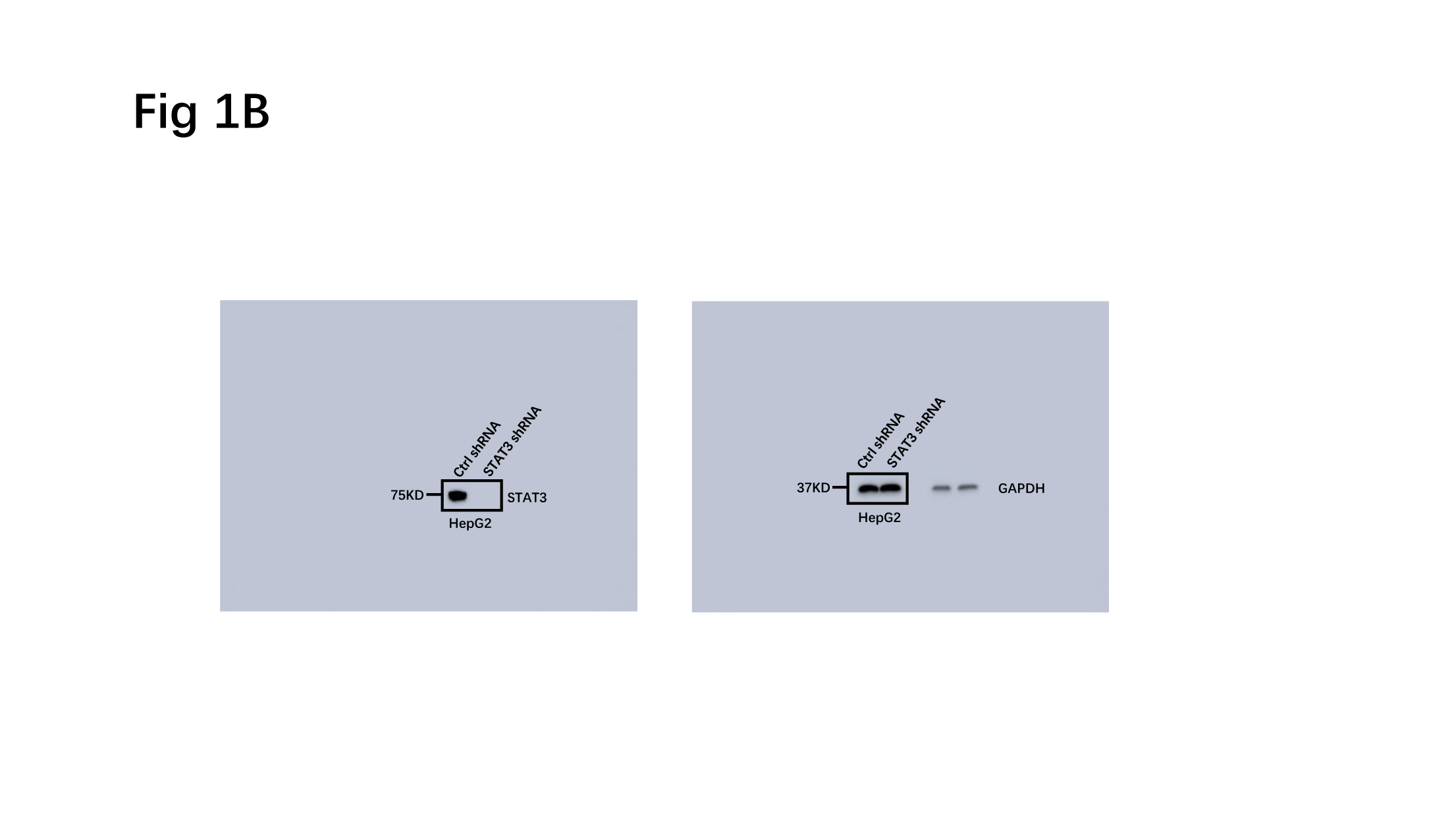

Supplement: Figure 1—source data 1. [file elife-82826-fig1-data1.zip › Figure 1-source data 1/Labeled Western blot/Figure 1B.tif]

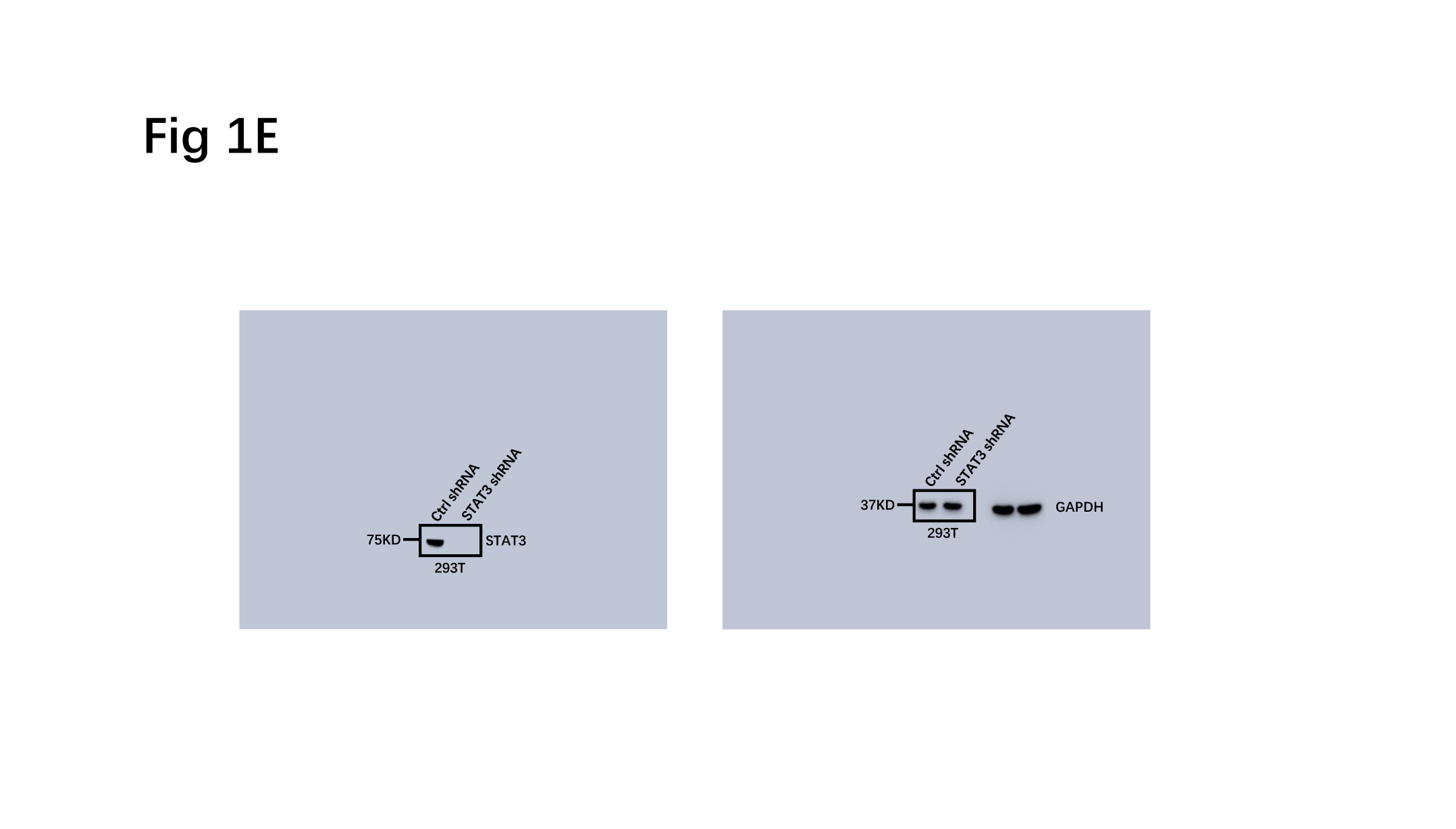

Supplement: Figure 1—source data 1. [file elife-82826-fig1-data1.zip › Figure 1-source data 1/Labeled Western blot/Figure 1E.tif]

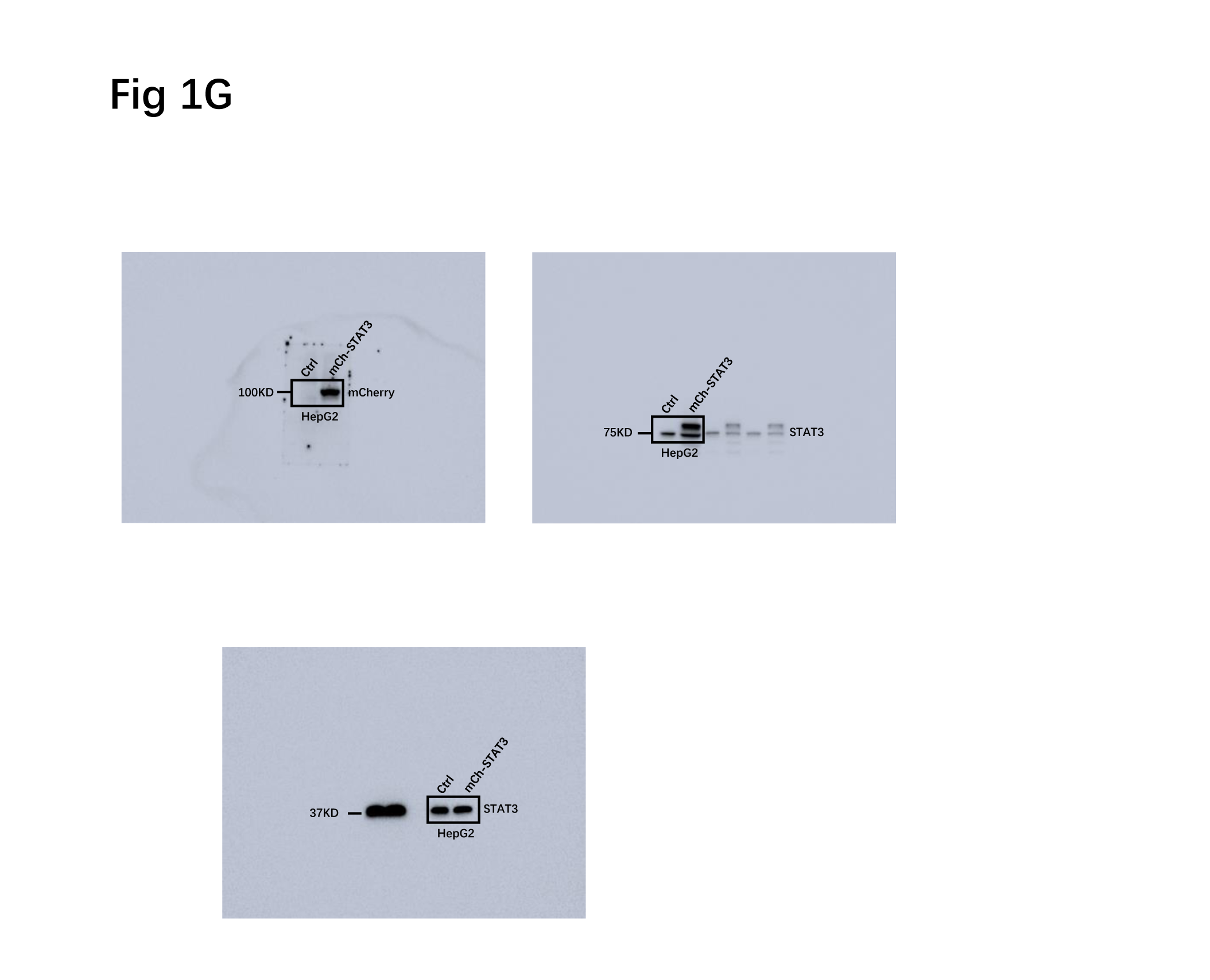

Supplement: Figure 1—source data 1. [file elife-82826-fig1-data1.zip › Figure 1-source data 1/Labeled Western blot/Figure 1G.tif]

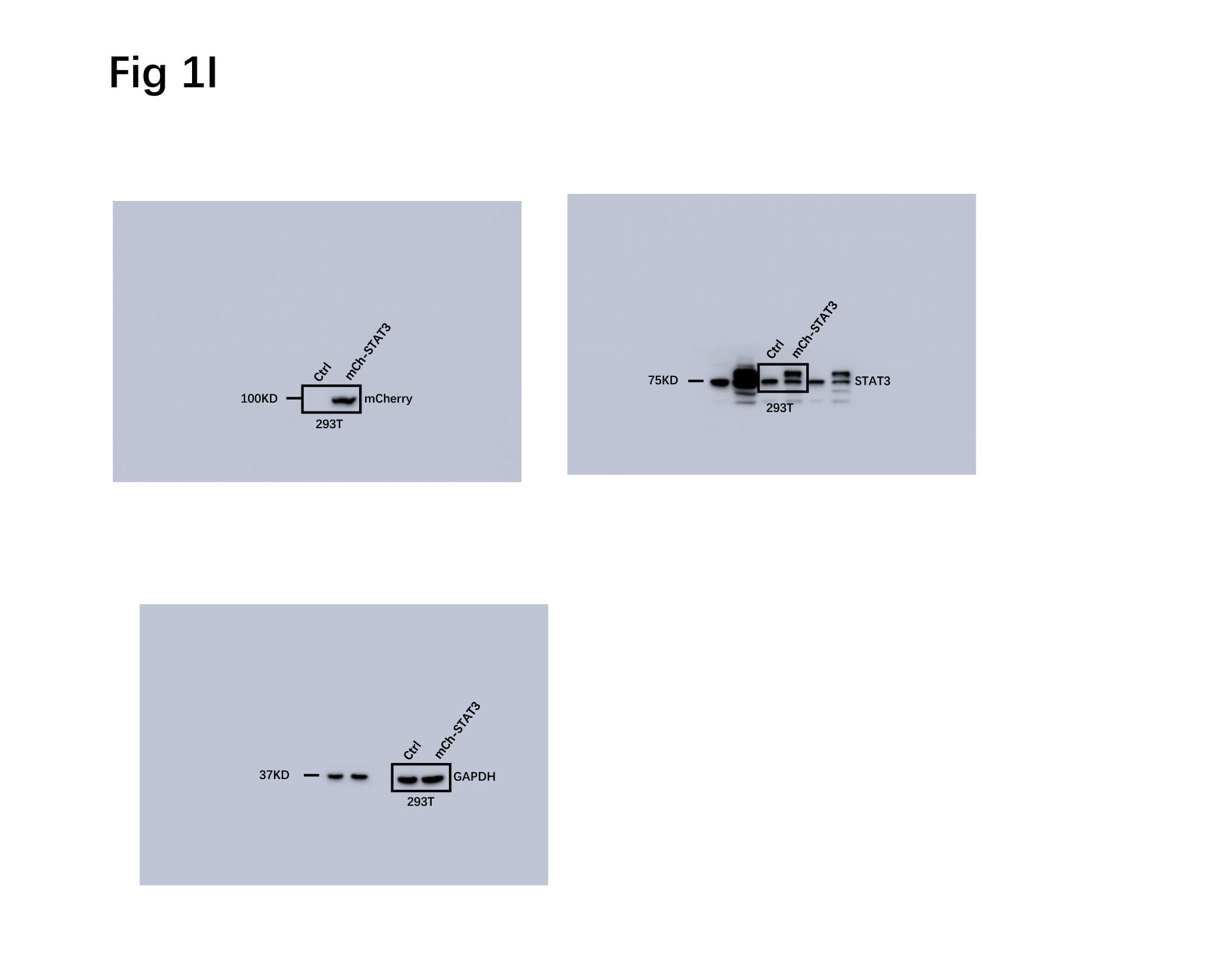

Supplement: Figure 1—source data 1. [file elife-82826-fig1-data1.zip › Figure 1-source data 1/Labeled Western blot/Figure 1I.tif]

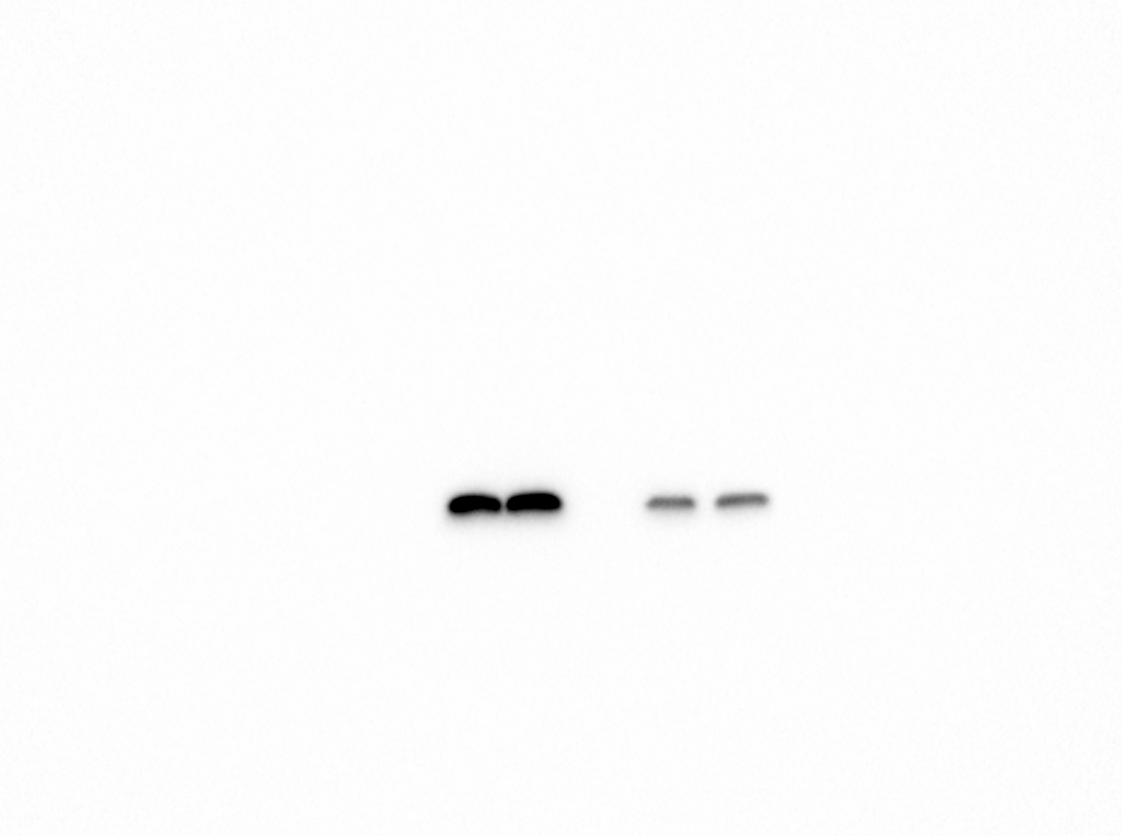

Supplement: Figure 1—source data 1. [file elife-82826-fig1-data1.zip › Figure 1-source data 1/Unlabeled Western blot/Figure 1B-GAPDH.tif]

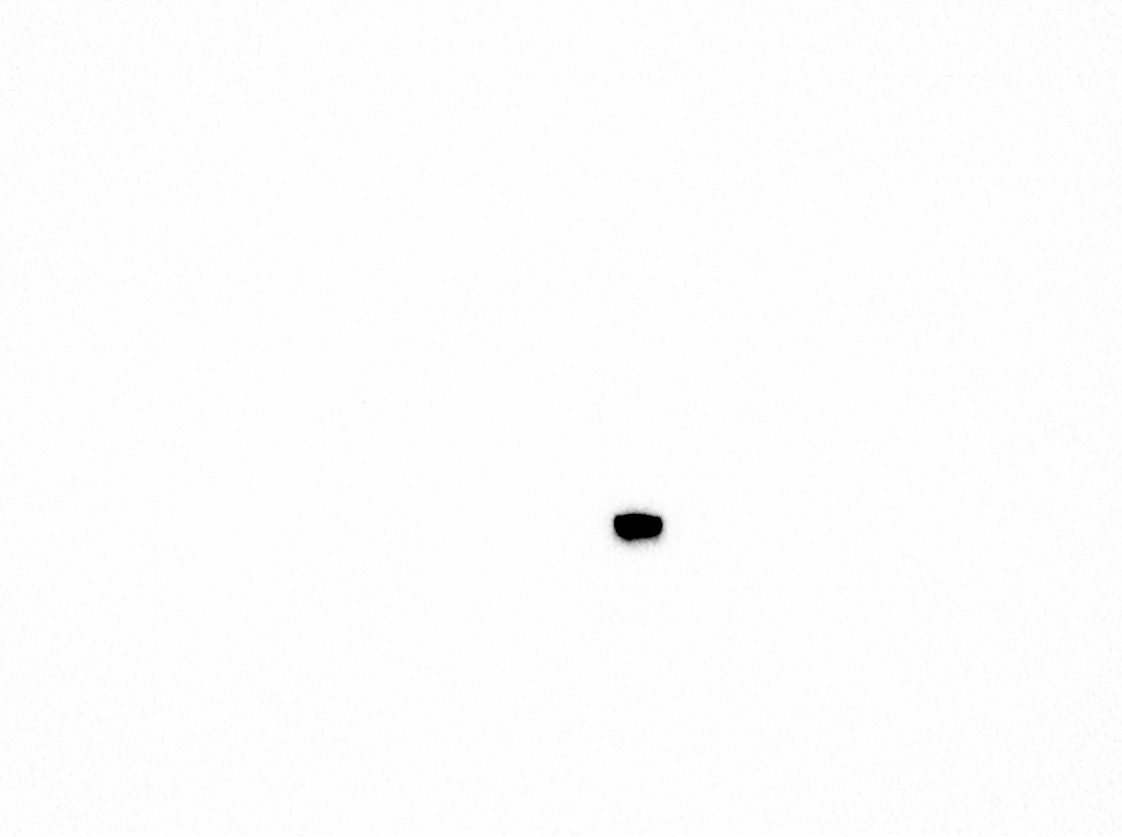

Supplement: Figure 1—source data 1. [file elife-82826-fig1-data1.zip › Figure 1-source data 1/Unlabeled Western blot/Figure 1B-STAT3.tif]

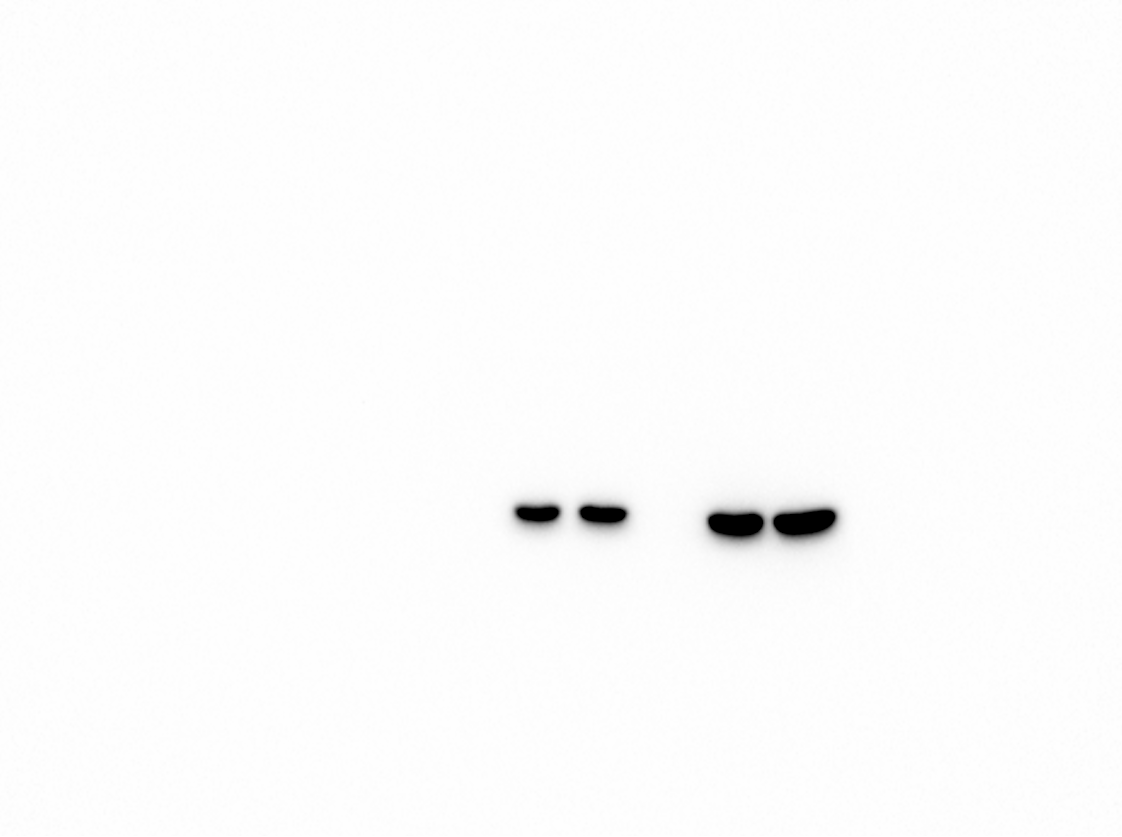

Supplement: Figure 1—source data 1. [file elife-82826-fig1-data1.zip › Figure 1-source data 1/Unlabeled Western blot/Figure 1E-GAPDH.tif]

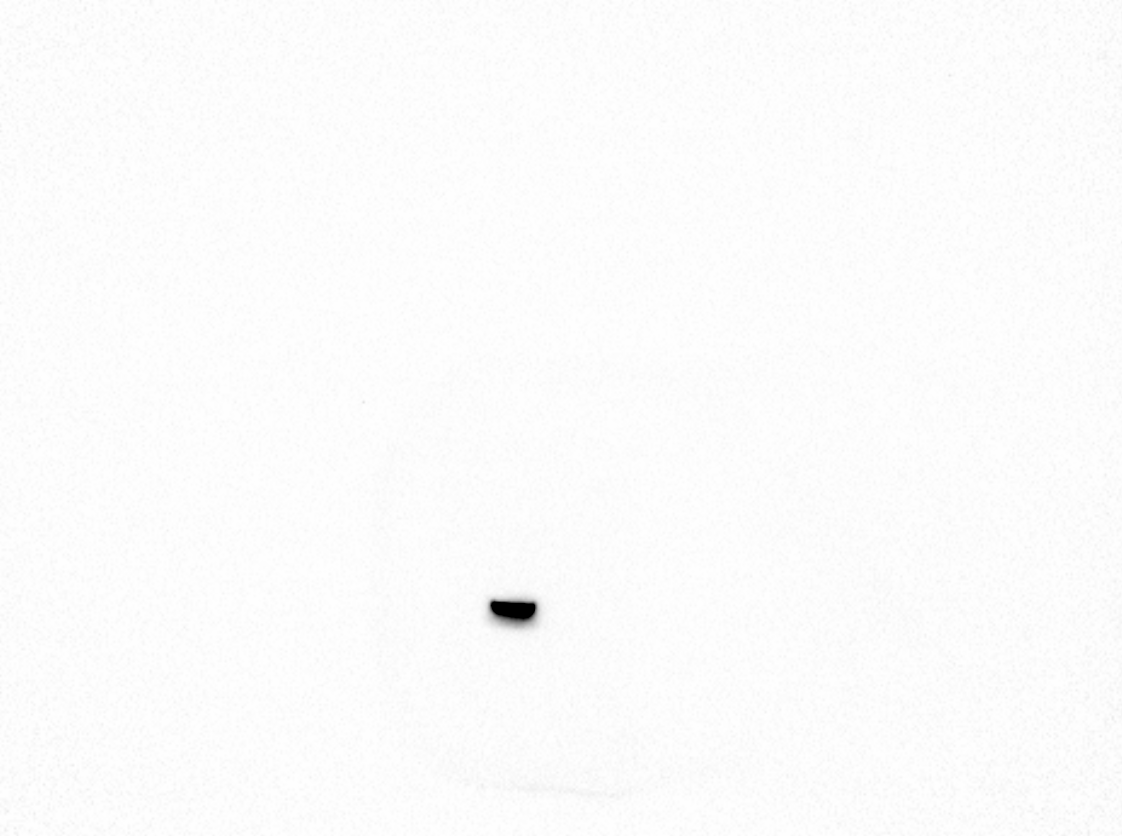

Supplement: Figure 1—source data 1. [file elife-82826-fig1-data1.zip › Figure 1-source data 1/Unlabeled Western blot/Figure 1E-STAT3.tif]

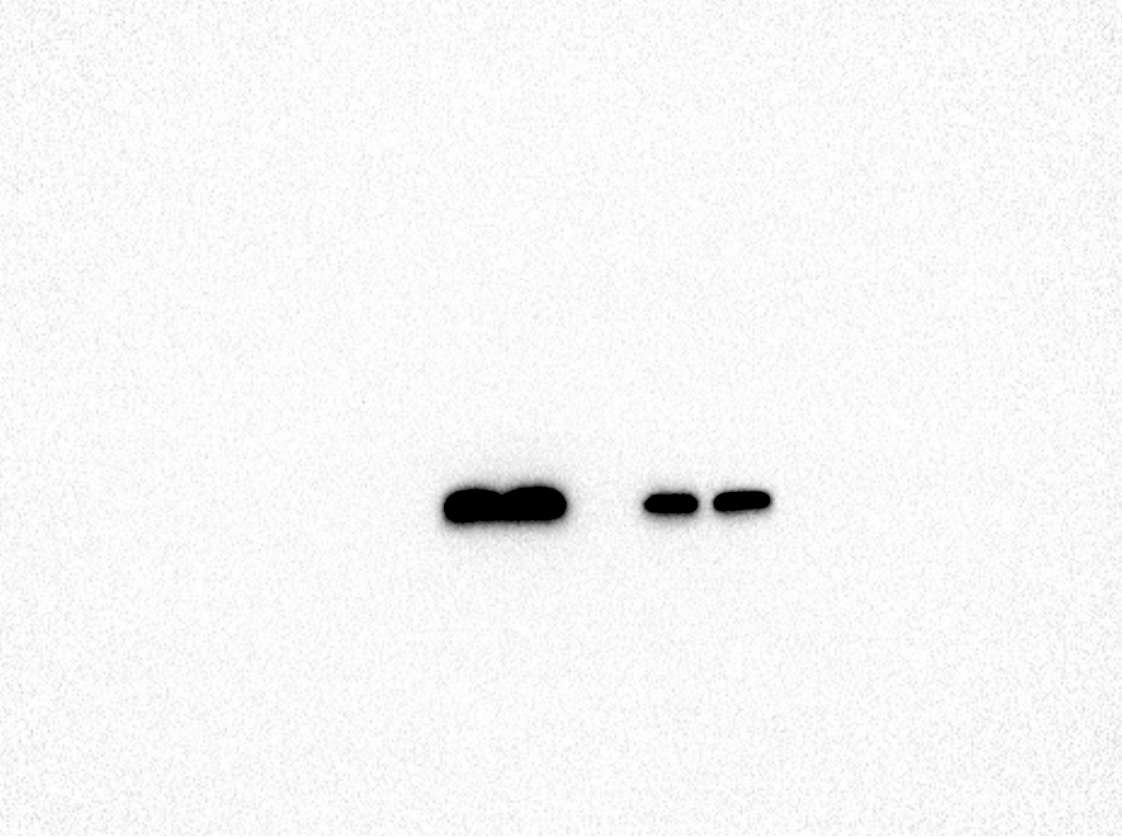

Supplement: Figure 1—source data 1. [file elife-82826-fig1-data1.zip › Figure 1-source data 1/Unlabeled Western blot/Figure 1G-GAPDH.tif]

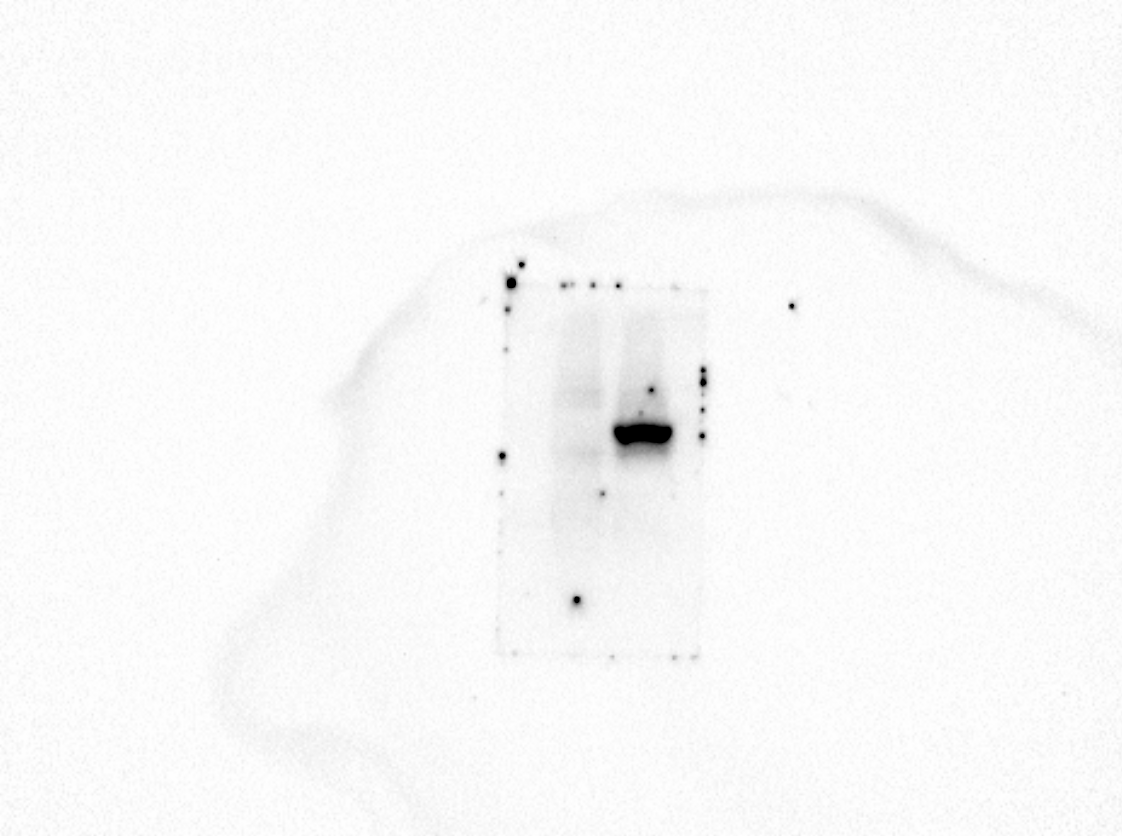

Supplement: Figure 1—source data 1. [file elife-82826-fig1-data1.zip › Figure 1-source data 1/Unlabeled Western blot/Figure 1G-mCherry.tif]

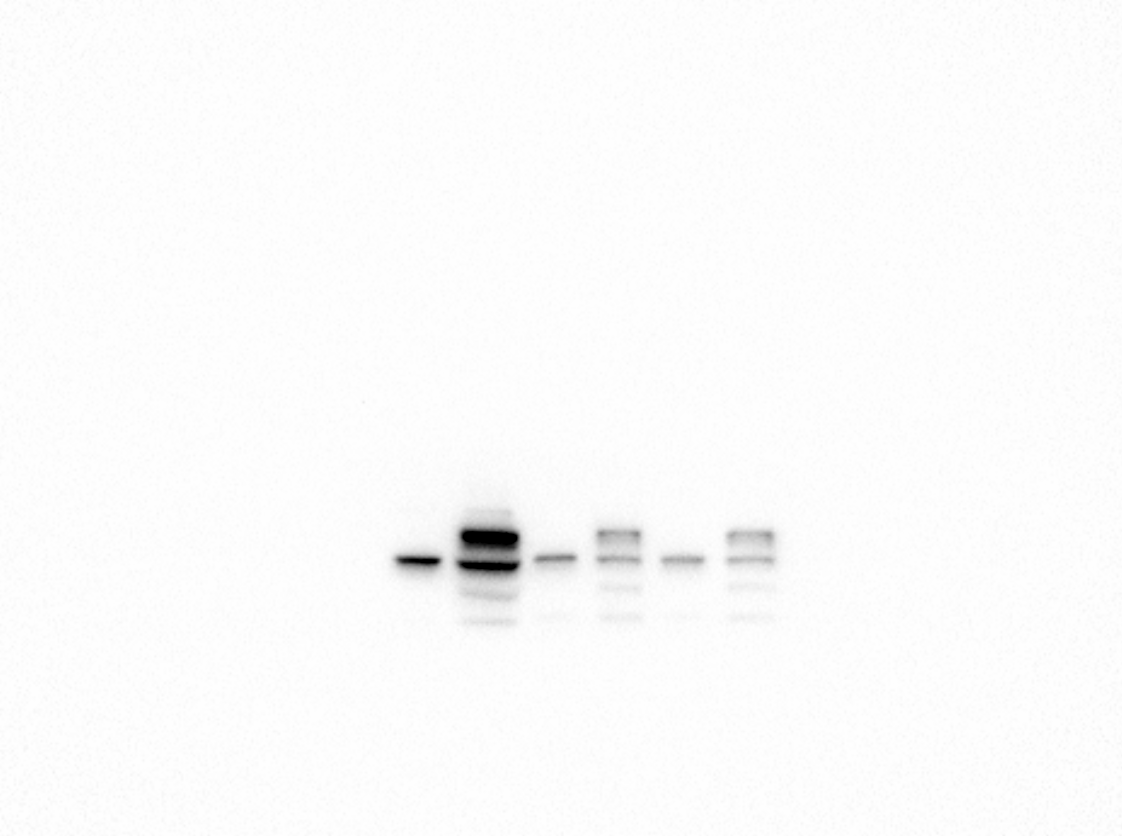

Supplement: Figure 1—source data 1. [file elife-82826-fig1-data1.zip › Figure 1-source data 1/Unlabeled Western blot/Figure 1G-STAT3.tif]

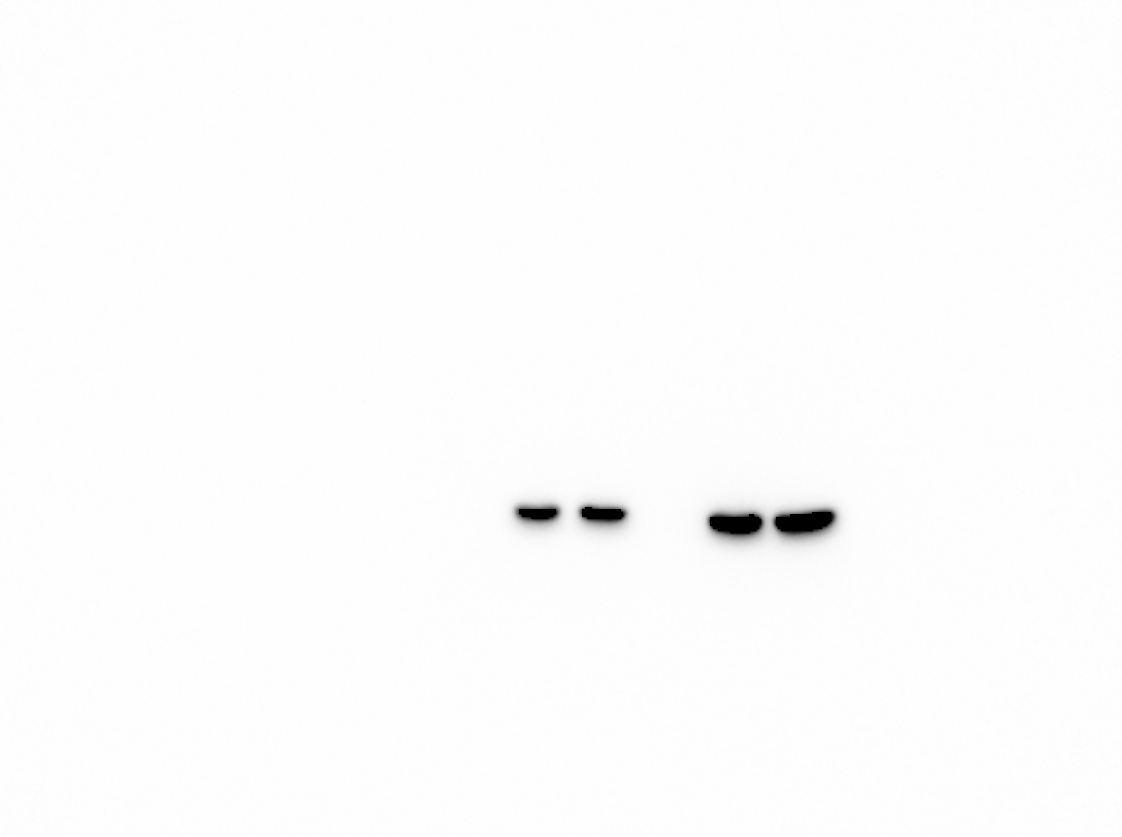

Supplement: Figure 1—source data 1. [file elife-82826-fig1-data1.zip › Figure 1-source data 1/Unlabeled Western blot/Figure 1I-GAPDH.tif]

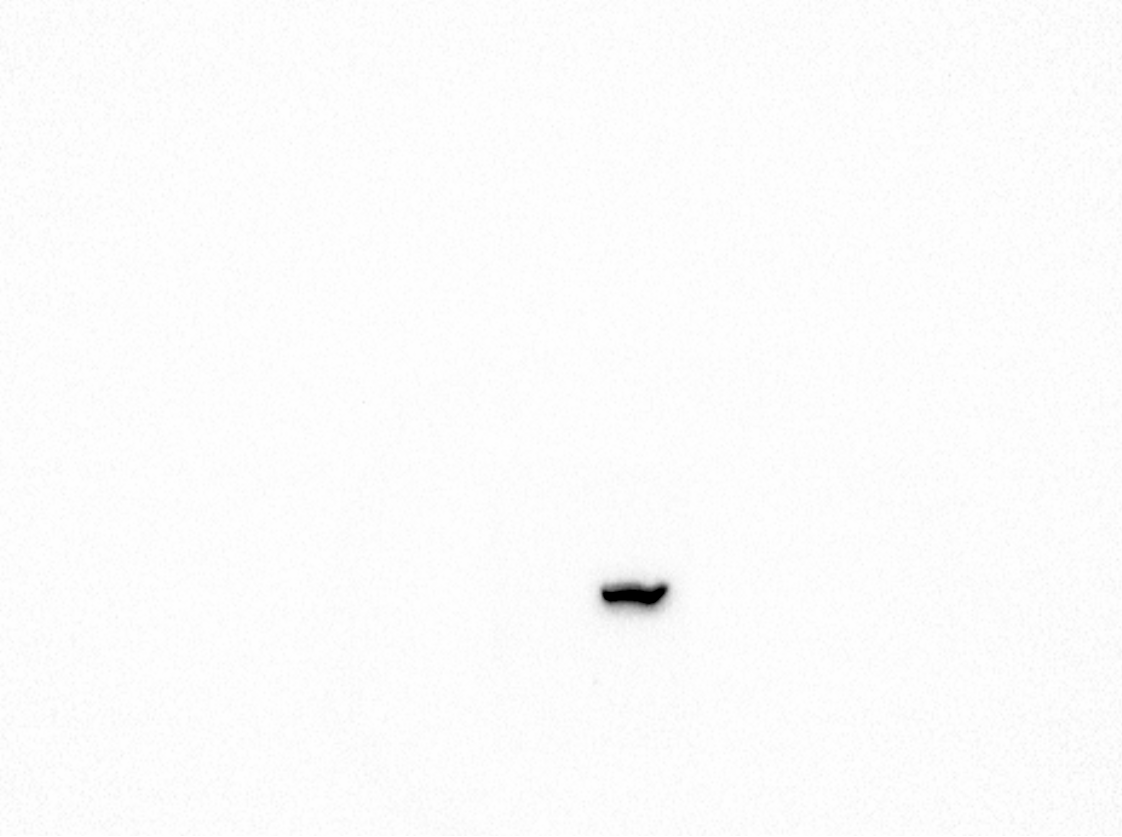

Supplement: Figure 1—source data 1. [file elife-82826-fig1-data1.zip › Figure 1-source data 1/Unlabeled Western blot/Figure 1I-mCherry.tif]

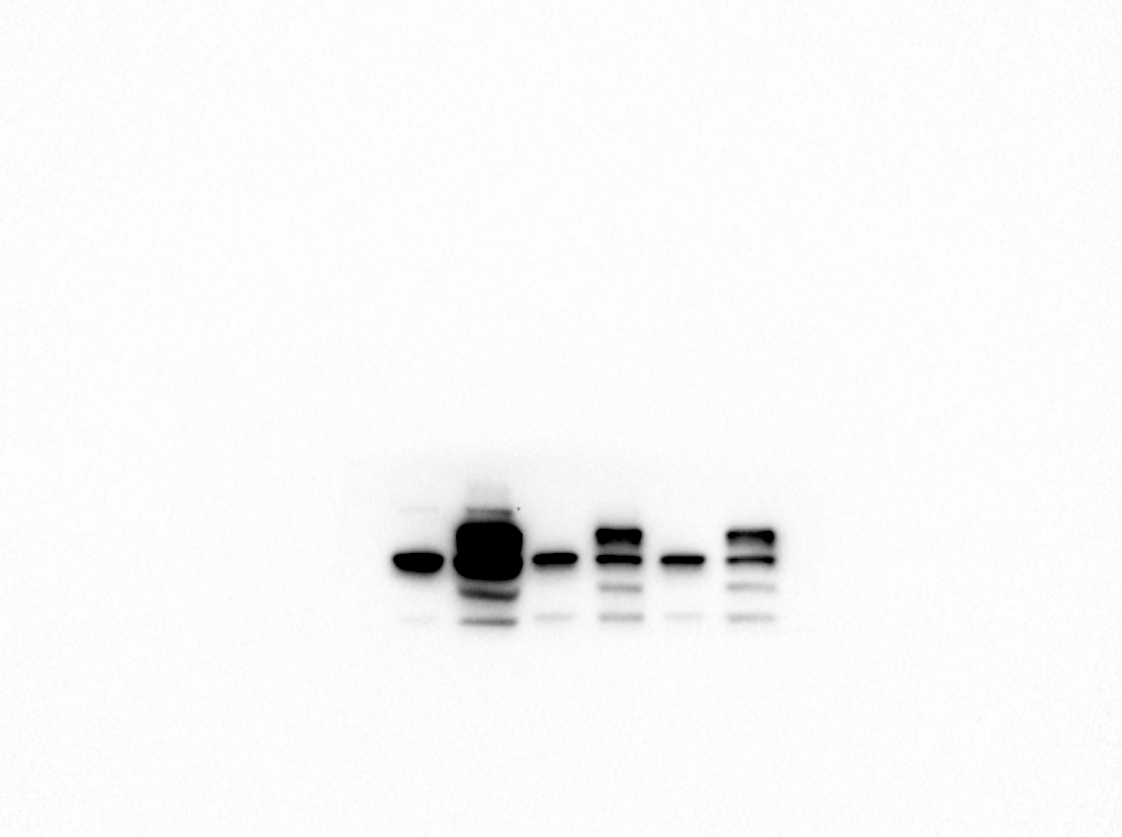

Supplement: Figure 1—source data 1. [file elife-82826-fig1-data1.zip › Figure 1-source data 1/Unlabeled Western blot/Figure 1I-STAT3.tif]

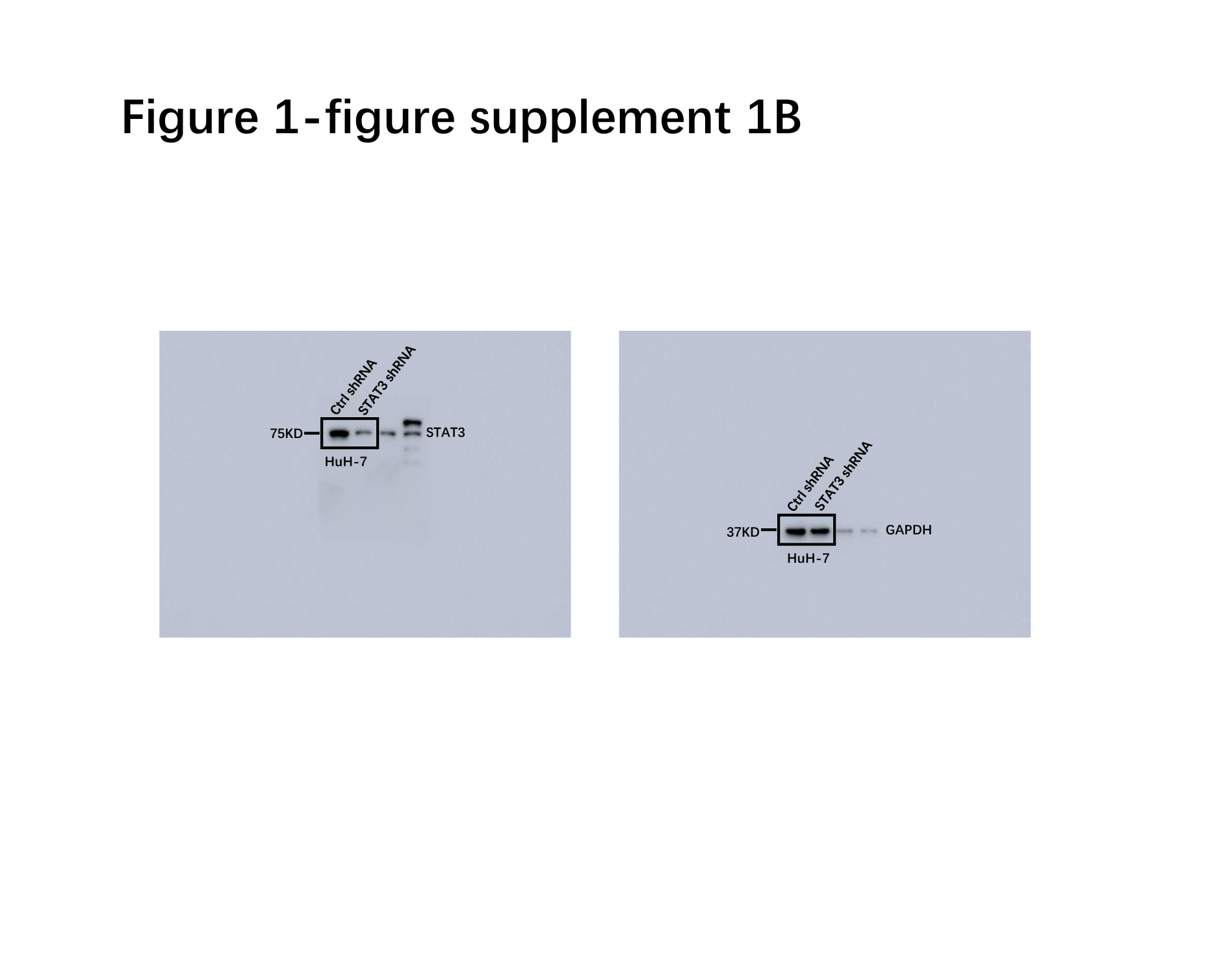

Supplement: Figure 1—figure supplement 1—source data 1. [file elife-82826-fig1-figsupp1-data1.zip › Figure 1-figure supplement 1-source data 1/Labeled Western blot/Figure 1-figure supplement 1B.tif]

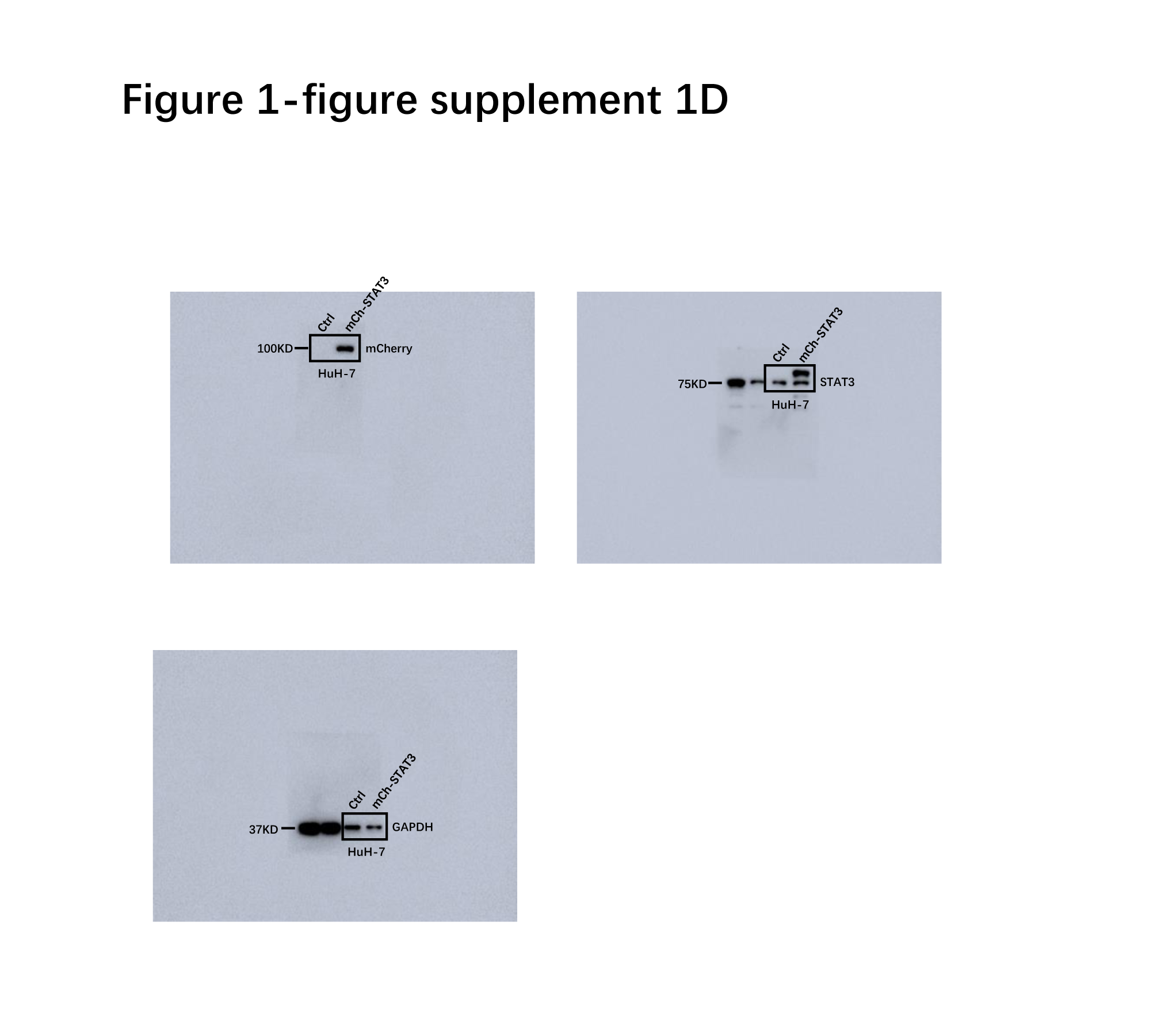

Supplement: Figure 1—figure supplement 1—source data 1. [file elife-82826-fig1-figsupp1-data1.zip › Figure 1-figure supplement 1-source data 1/Labeled Western blot/Figure 1-figure supplement 1D.tif]

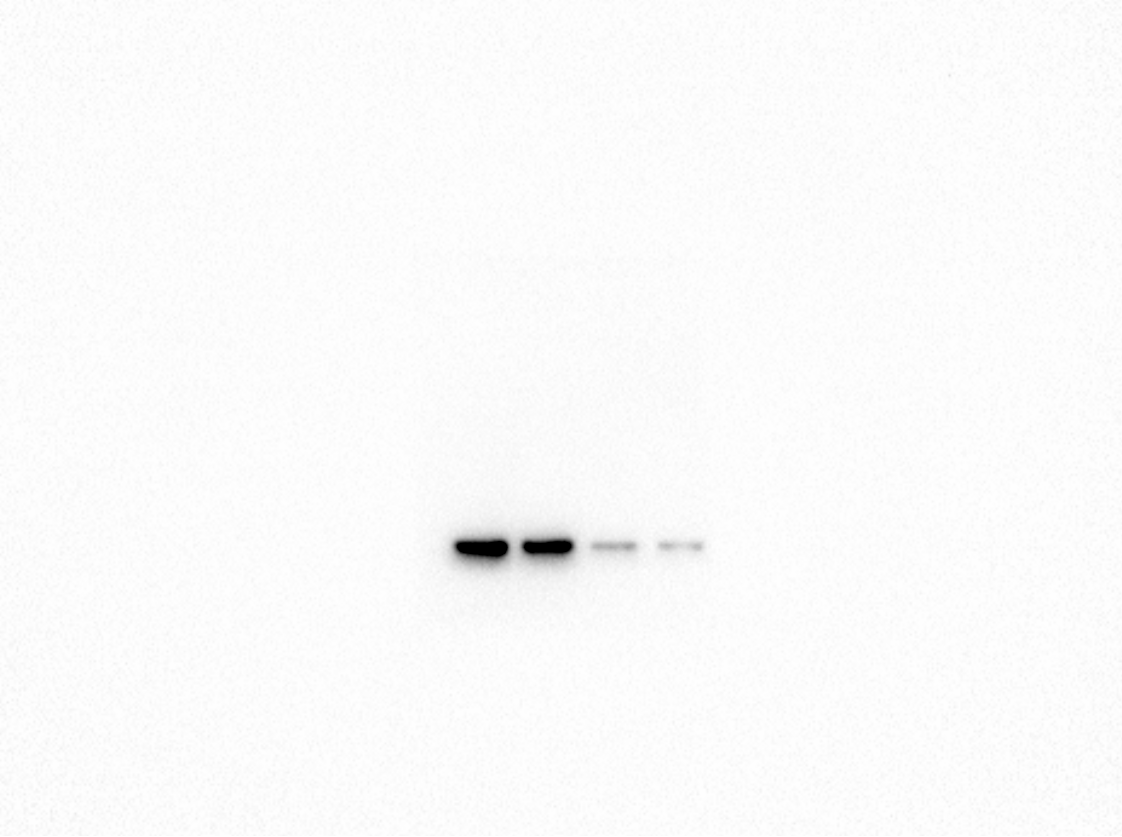

Supplement: Figure 1—figure supplement 1—source data 1. [file elife-82826-fig1-figsupp1-data1.zip › Figure 1-figure supplement 1-source data 1/Unlabeled Western blot/Fig 1-fig sup 1B-GAPDH.tif]

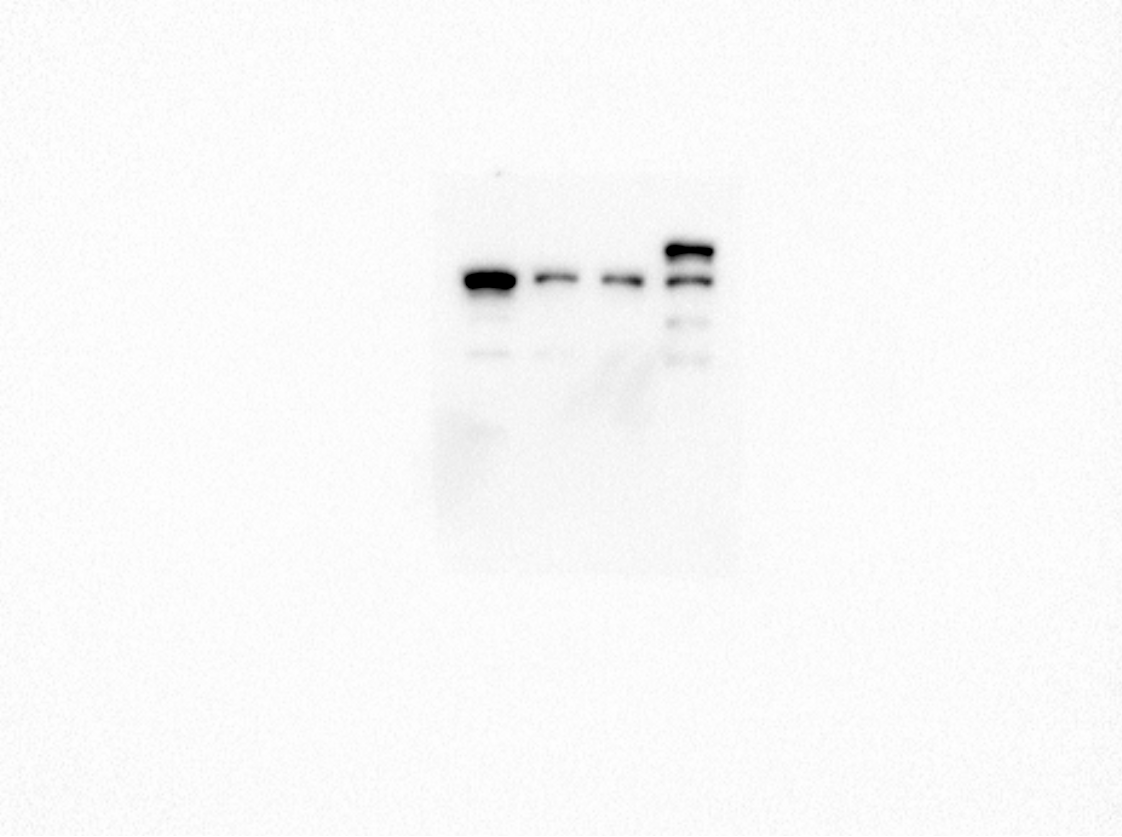

Supplement: Figure 1—figure supplement 1—source data 1. [file elife-82826-fig1-figsupp1-data1.zip › Figure 1-figure supplement 1-source data 1/Unlabeled Western blot/Fig 1-fig sup 1B-STAT3.tif]

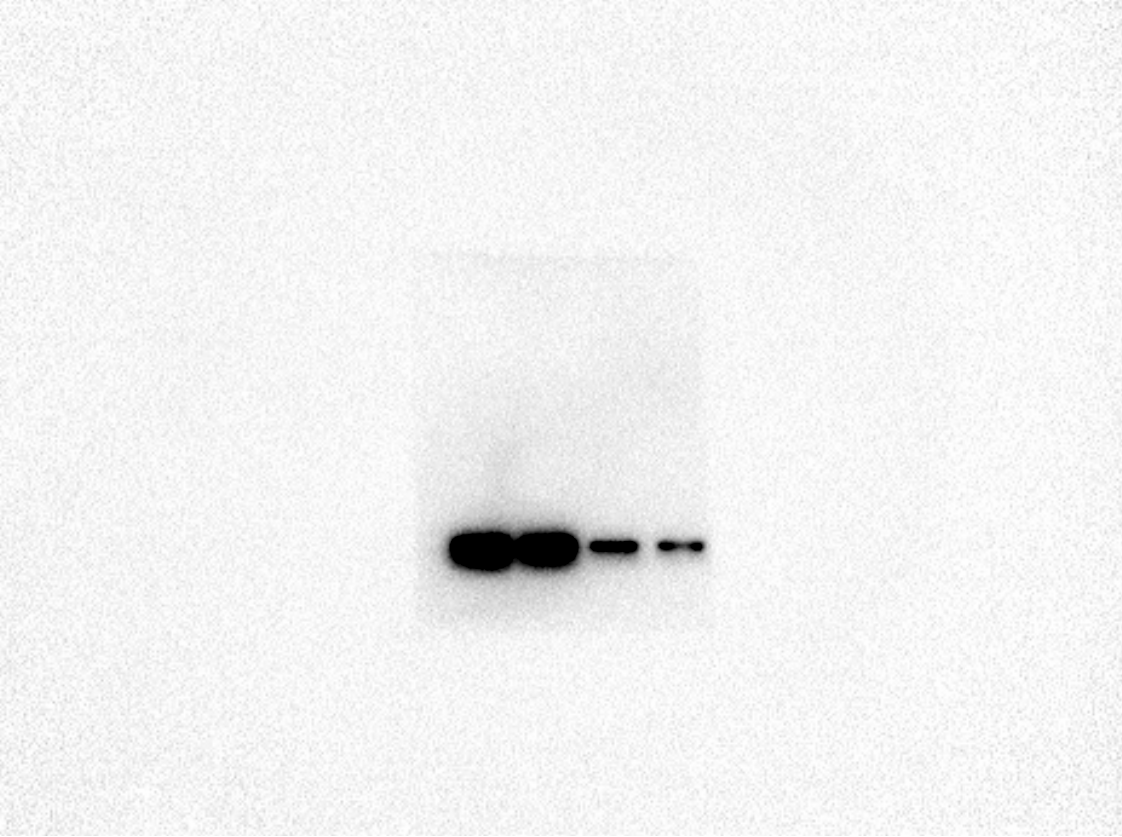

Supplement: Figure 1—figure supplement 1—source data 1. [file elife-82826-fig1-figsupp1-data1.zip › Figure 1-figure supplement 1-source data 1/Unlabeled Western blot/Fig 1-fig sup 1D-GAPDH.tif]

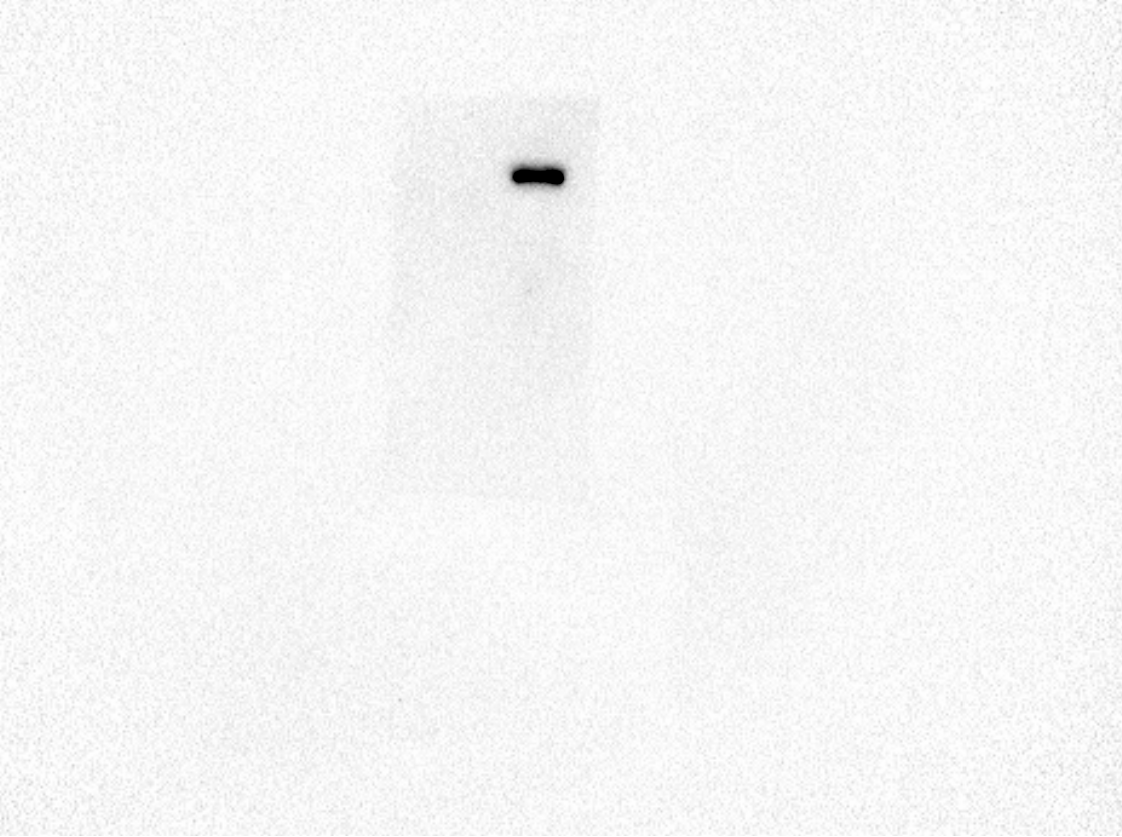

Supplement: Figure 1—figure supplement 1—source data 1. [file elife-82826-fig1-figsupp1-data1.zip › Figure 1-figure supplement 1-source data 1/Unlabeled Western blot/Fig1-fig sup 1D-mCherry.tif]

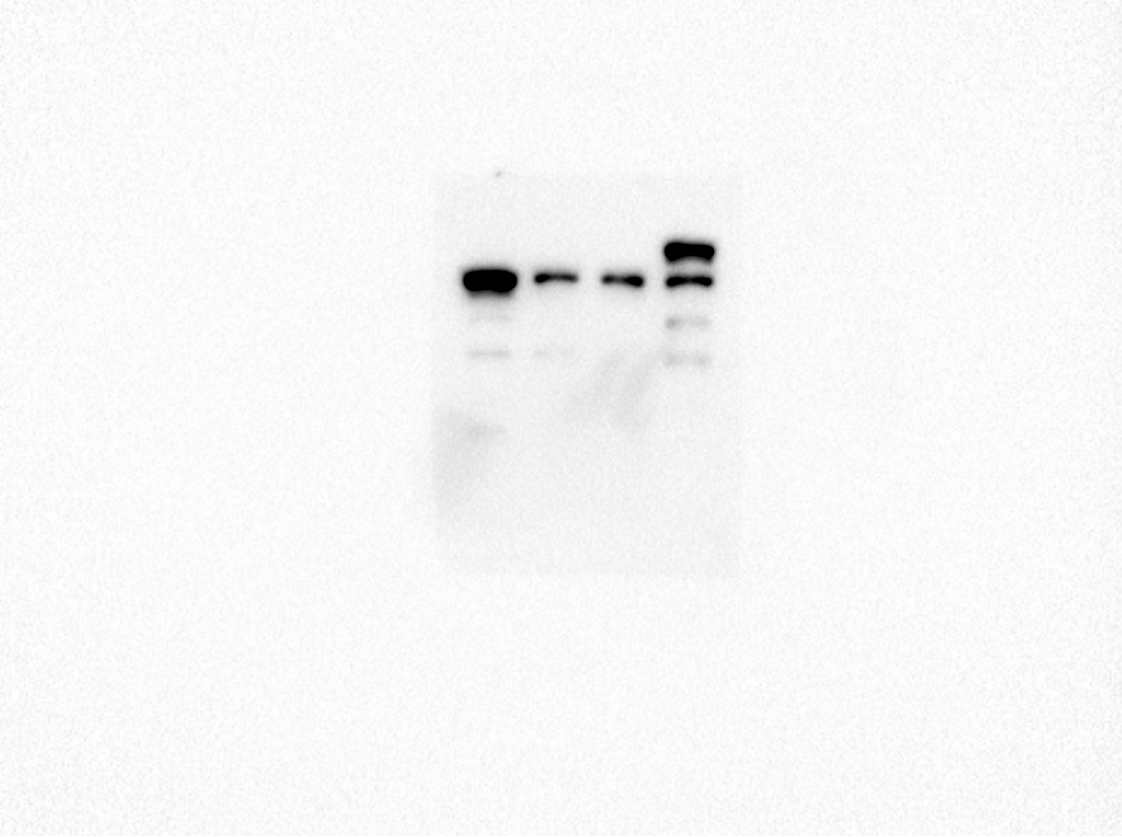

Supplement: Figure 1—figure supplement 1—source data 1. [file elife-82826-fig1-figsupp1-data1.zip › Figure 1-figure supplement 1-source data 1/Unlabeled Western blot/Fig1-fig sup 1D-STAT3.tif]

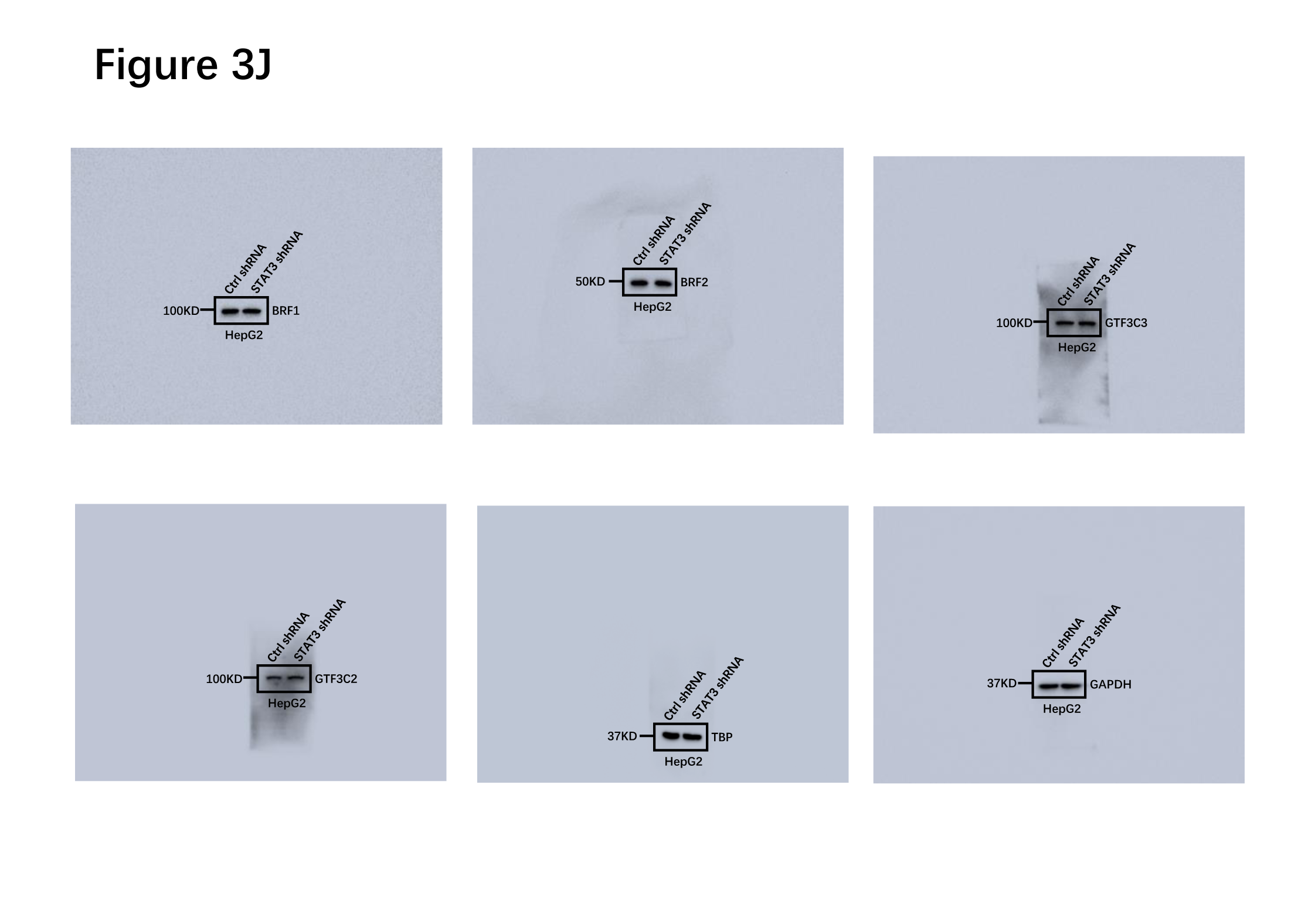

Supplement: Figure 3—source data 1. [file elife-82826-fig3-data1.zip › Figure 3-source data1/Labeled Western blot/Figure 3J.tif]

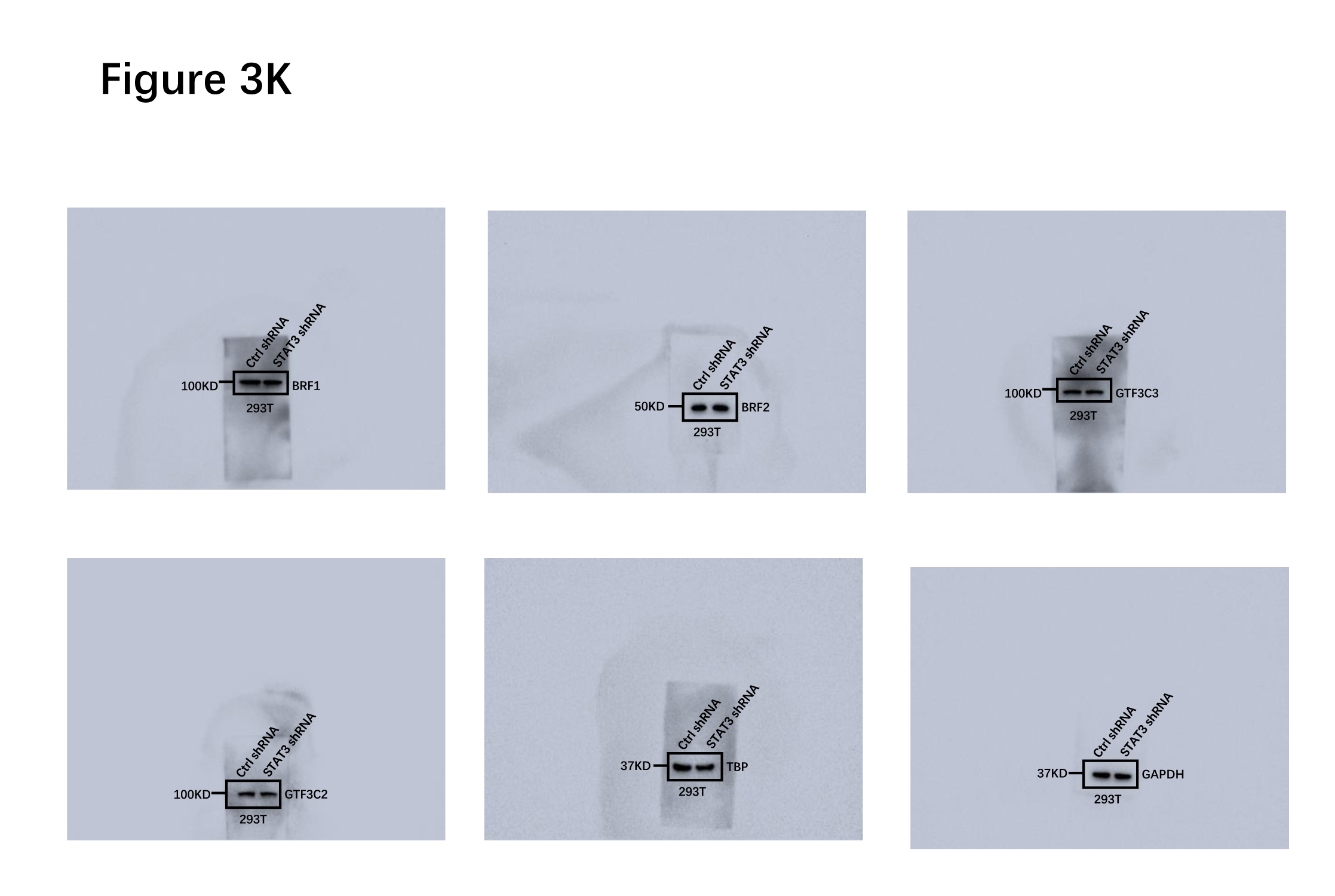

Supplement: Figure 3—source data 1. [file elife-82826-fig3-data1.zip › Figure 3-source data1/Labeled Western blot/Figure 3K.tif]

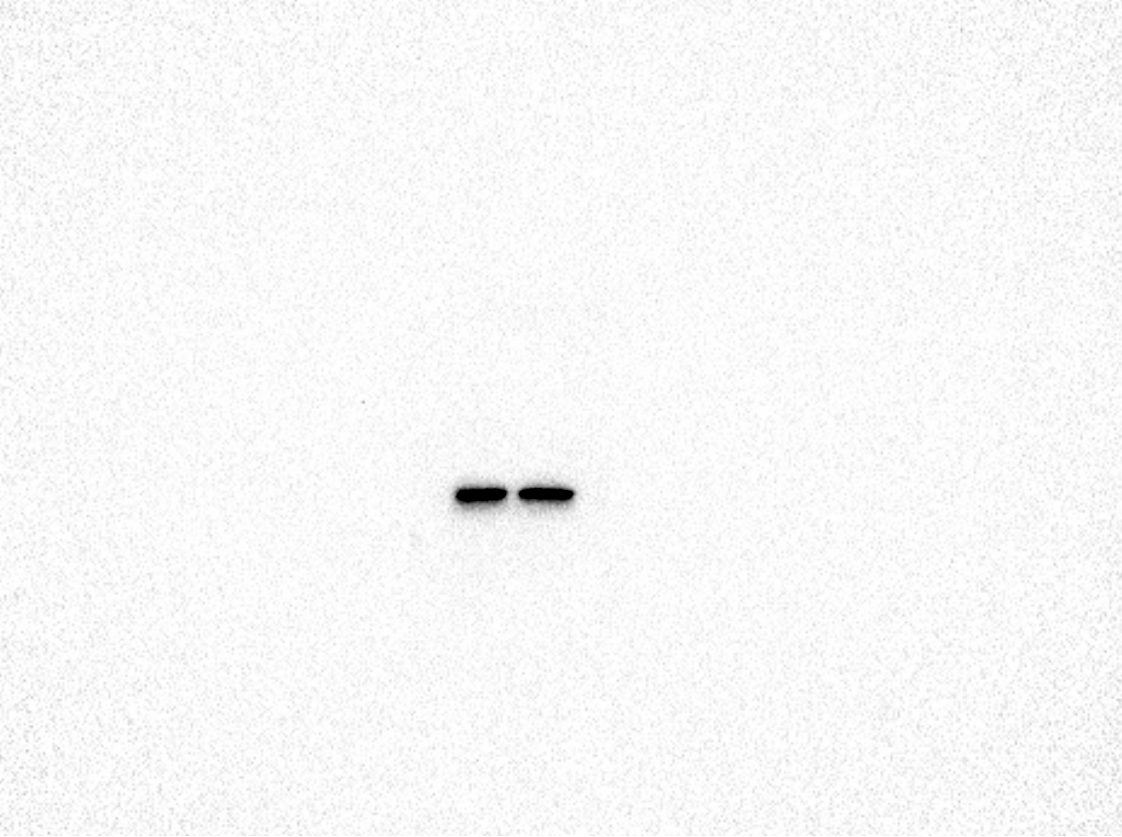

Supplement: Figure 3—source data 1. [file elife-82826-fig3-data1.zip › Figure 3-source data1/Unlabeled Western blot/Figure 3J-BRF1.tif]

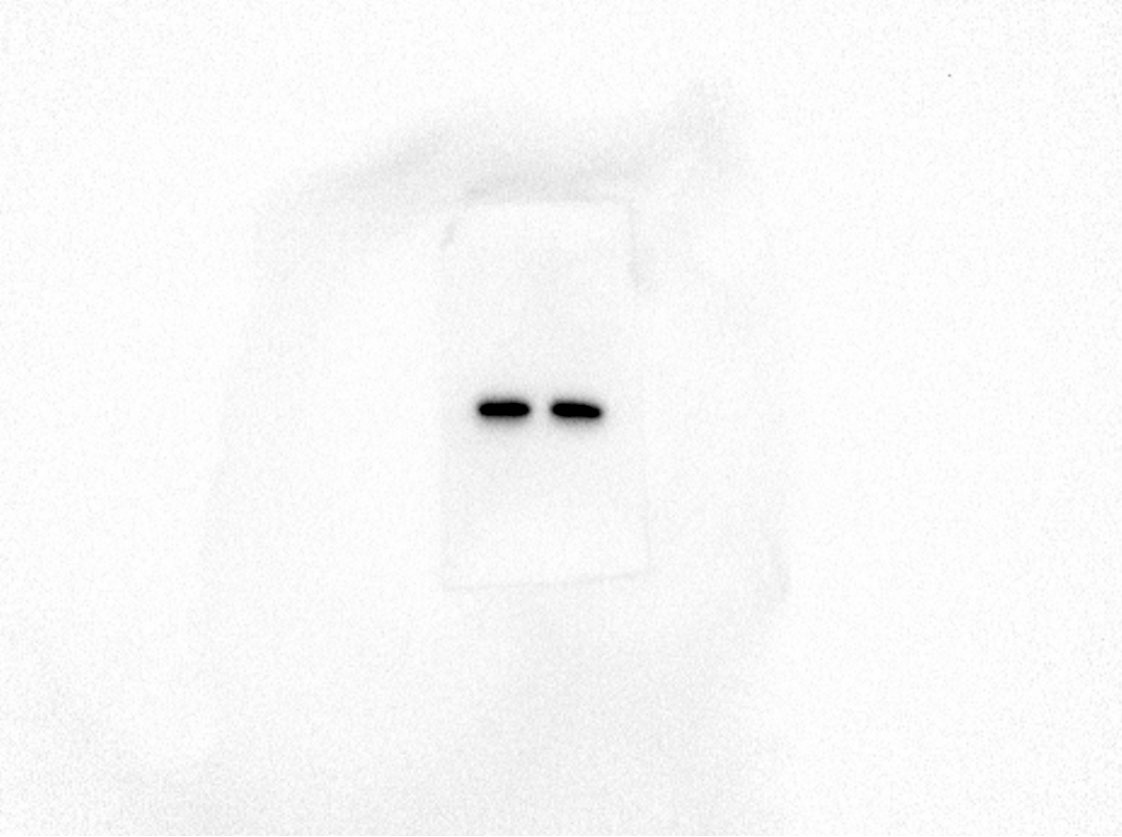

Supplement: Figure 3—source data 1. [file elife-82826-fig3-data1.zip › Figure 3-source data1/Unlabeled Western blot/Figure 3J-BRF2.tif]

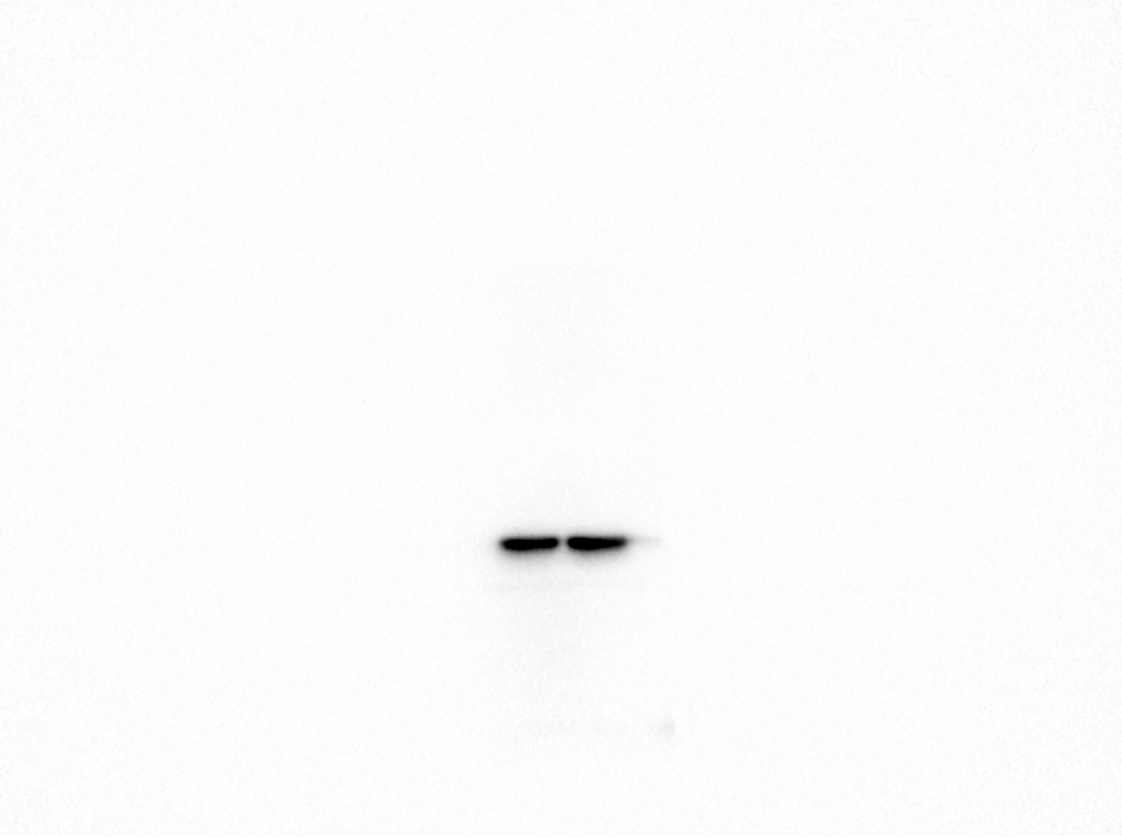

Supplement: Figure 3—source data 1. [file elife-82826-fig3-data1.zip › Figure 3-source data1/Unlabeled Western blot/Figure 3J-GAPDH.tif]

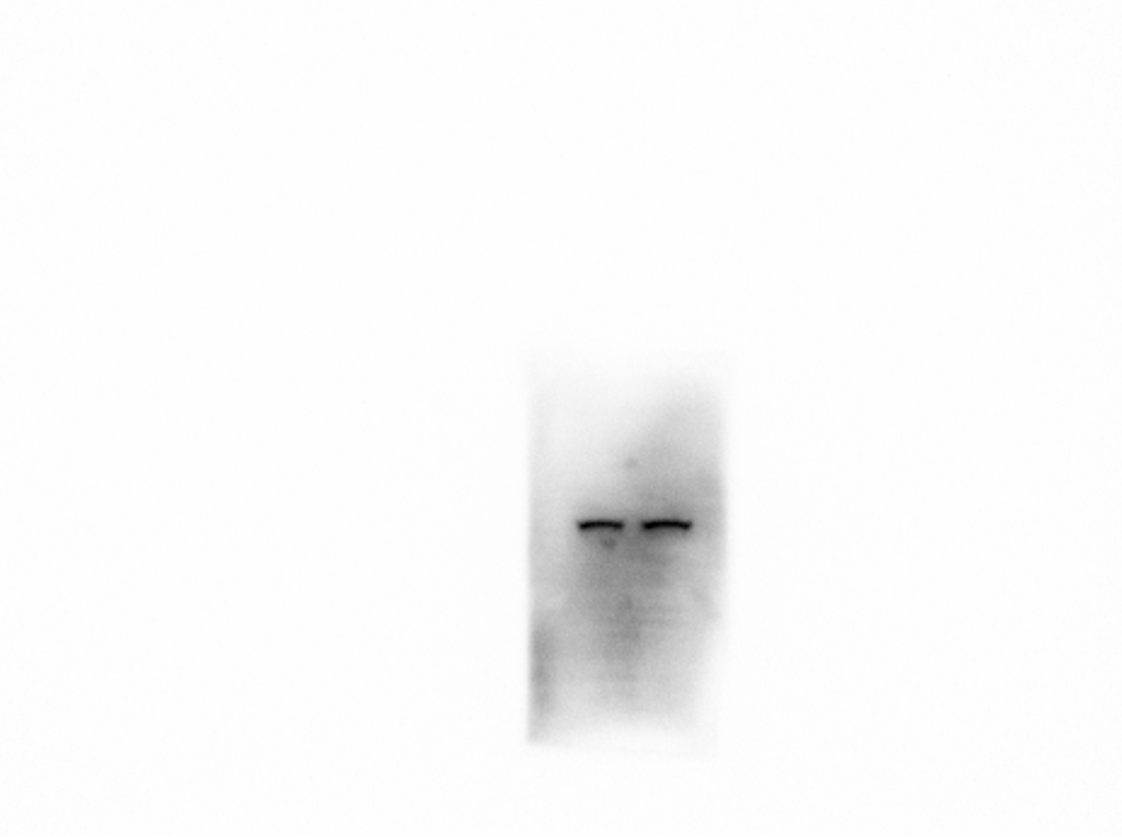

Supplement: Figure 3—source data 1. [file elife-82826-fig3-data1.zip › Figure 3-source data1/Unlabeled Western blot/Figure 3J-GTF3C2.tif]

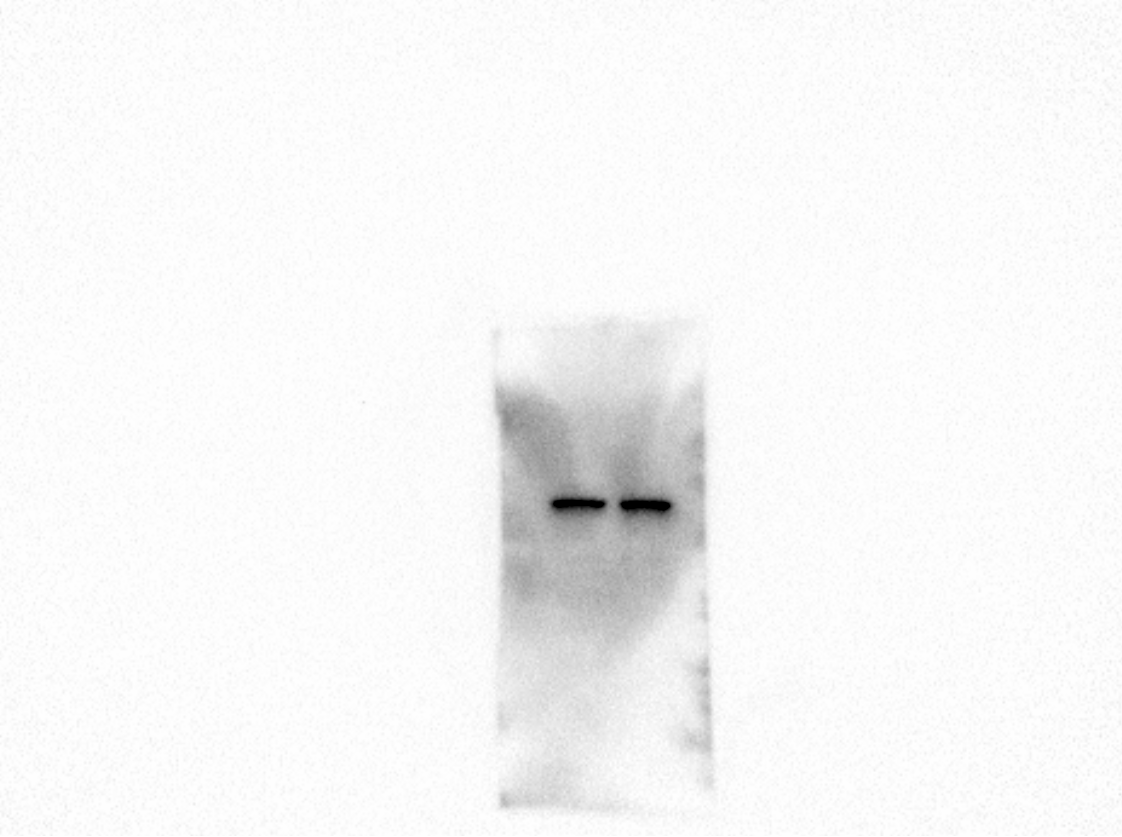

Supplement: Figure 3—source data 1. [file elife-82826-fig3-data1.zip › Figure 3-source data1/Unlabeled Western blot/Figure 3J-GTF3C3.tif]

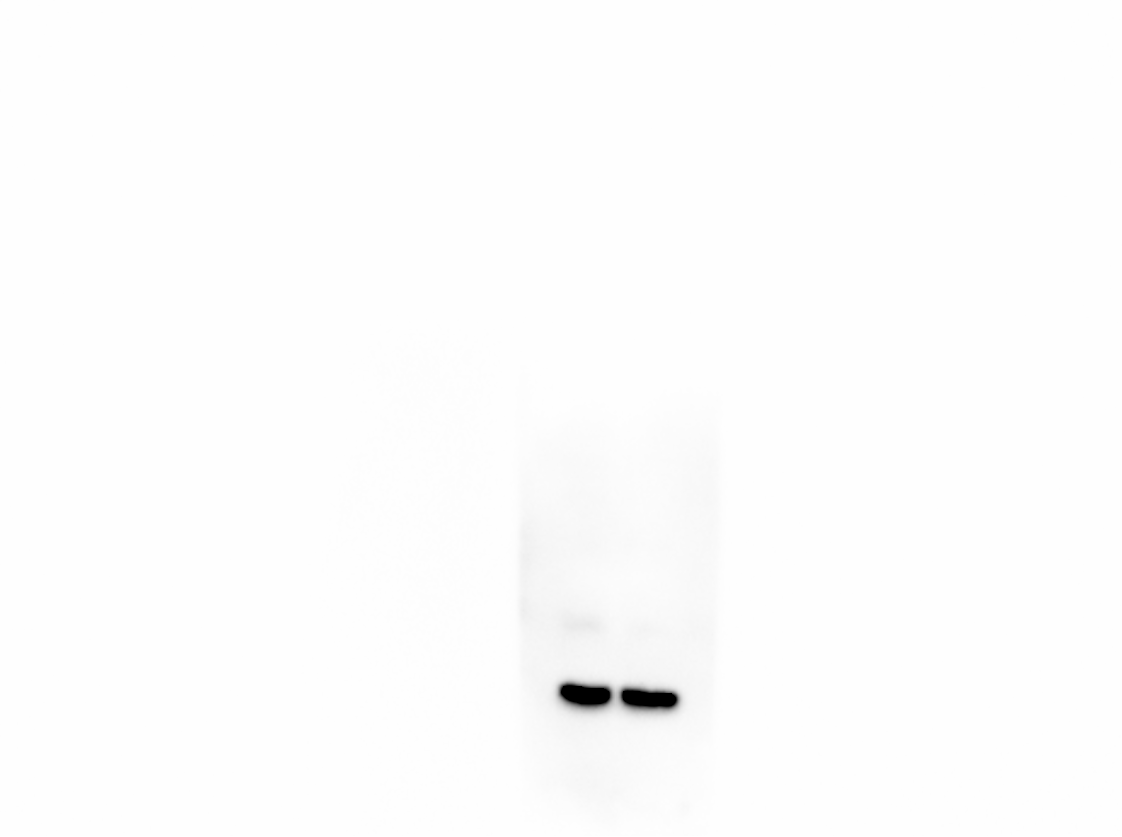

Supplement: Figure 3—source data 1. [file elife-82826-fig3-data1.zip › Figure 3-source data1/Unlabeled Western blot/Figure 3J-TBP.tif]

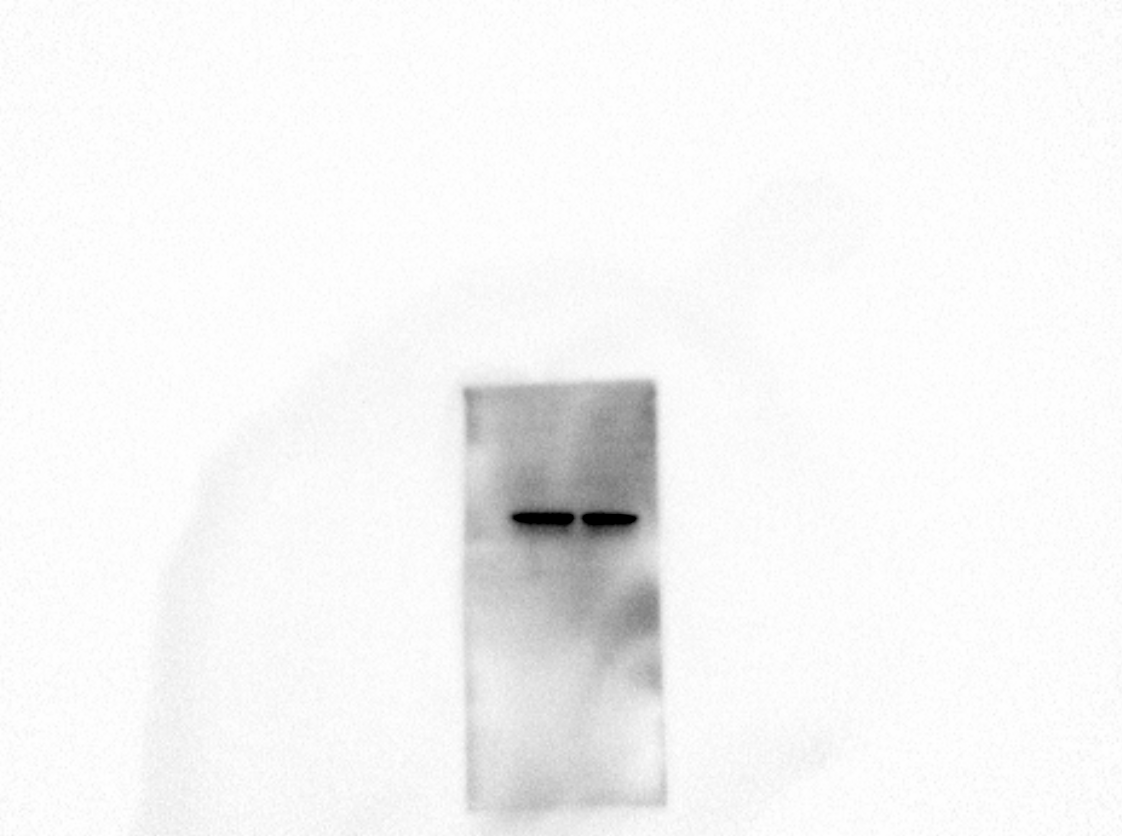

Supplement: Figure 3—source data 1. [file elife-82826-fig3-data1.zip › Figure 3-source data1/Unlabeled Western blot/Figure 3K-BRF1.tif]

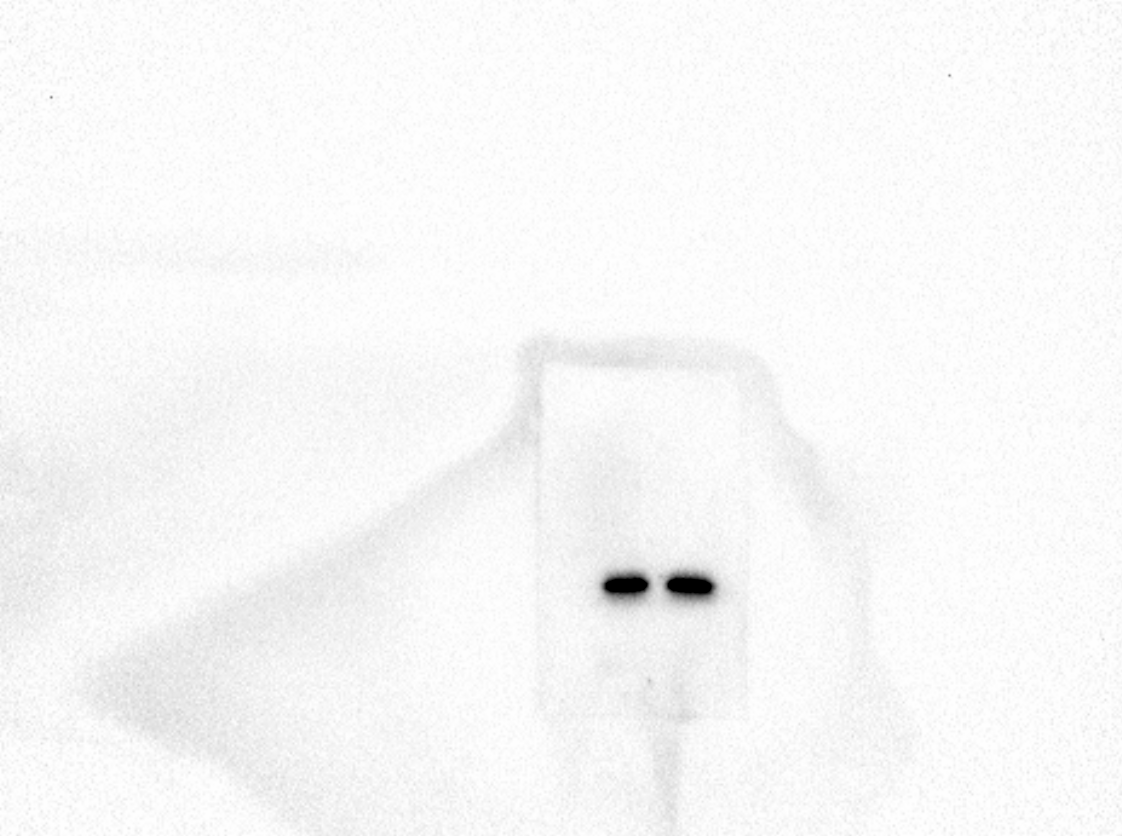

Supplement: Figure 3—source data 1. [file elife-82826-fig3-data1.zip › Figure 3-source data1/Unlabeled Western blot/Figure 3K-BRF2.tif]

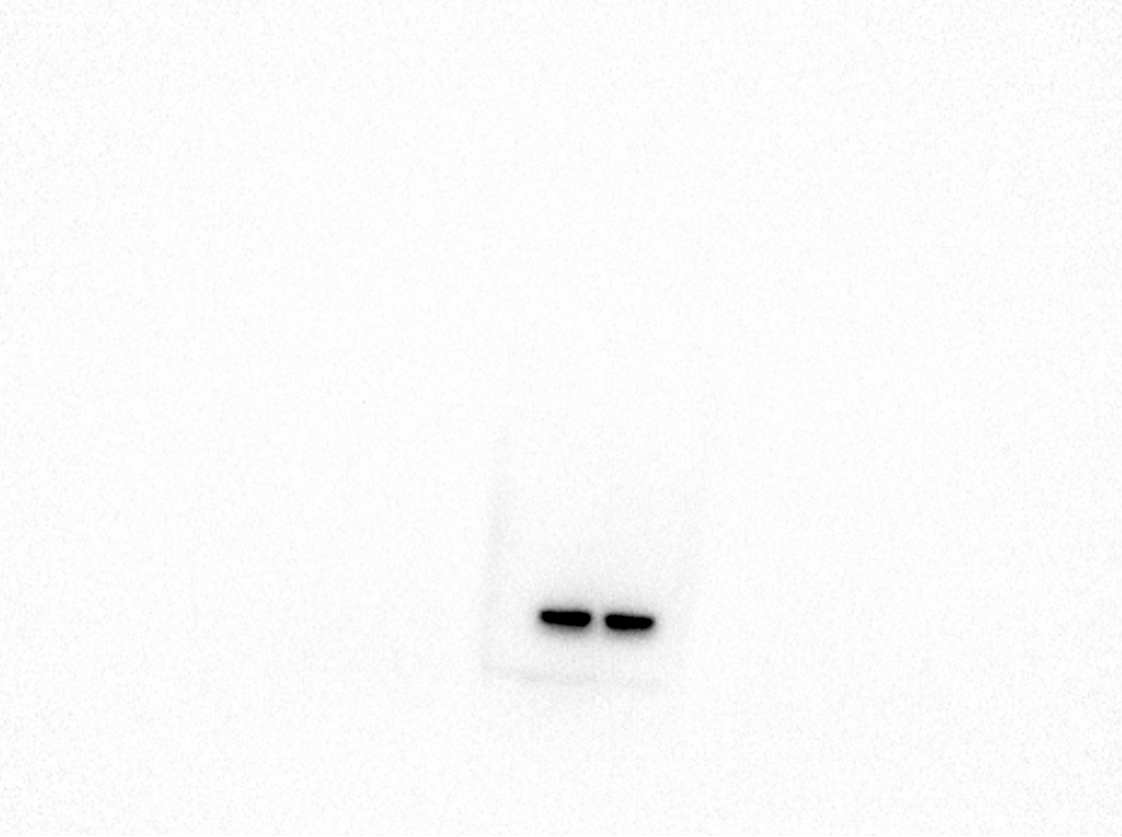

Supplement: Figure 3—source data 1. [file elife-82826-fig3-data1.zip › Figure 3-source data1/Unlabeled Western blot/Figure 3K-GAPDH.tif]

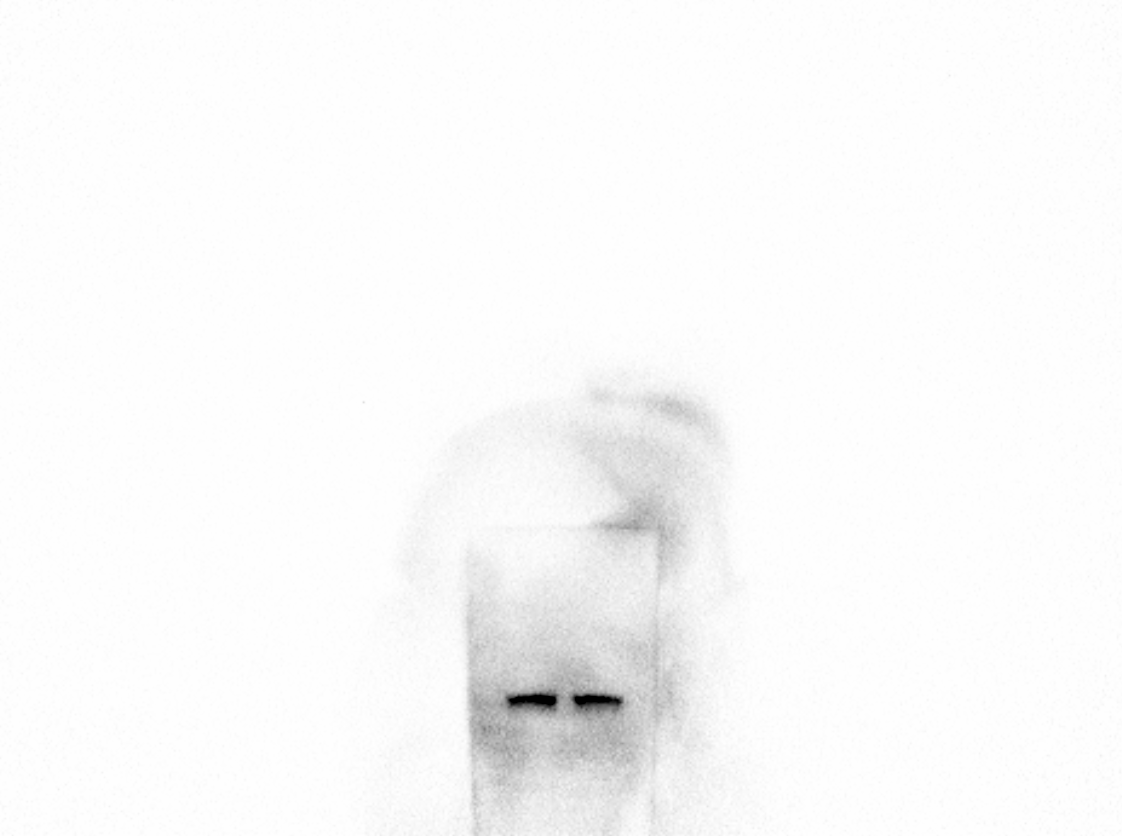

Supplement: Figure 3—source data 1. [file elife-82826-fig3-data1.zip › Figure 3-source data1/Unlabeled Western blot/Figure 3K-GTF3C2.tif]

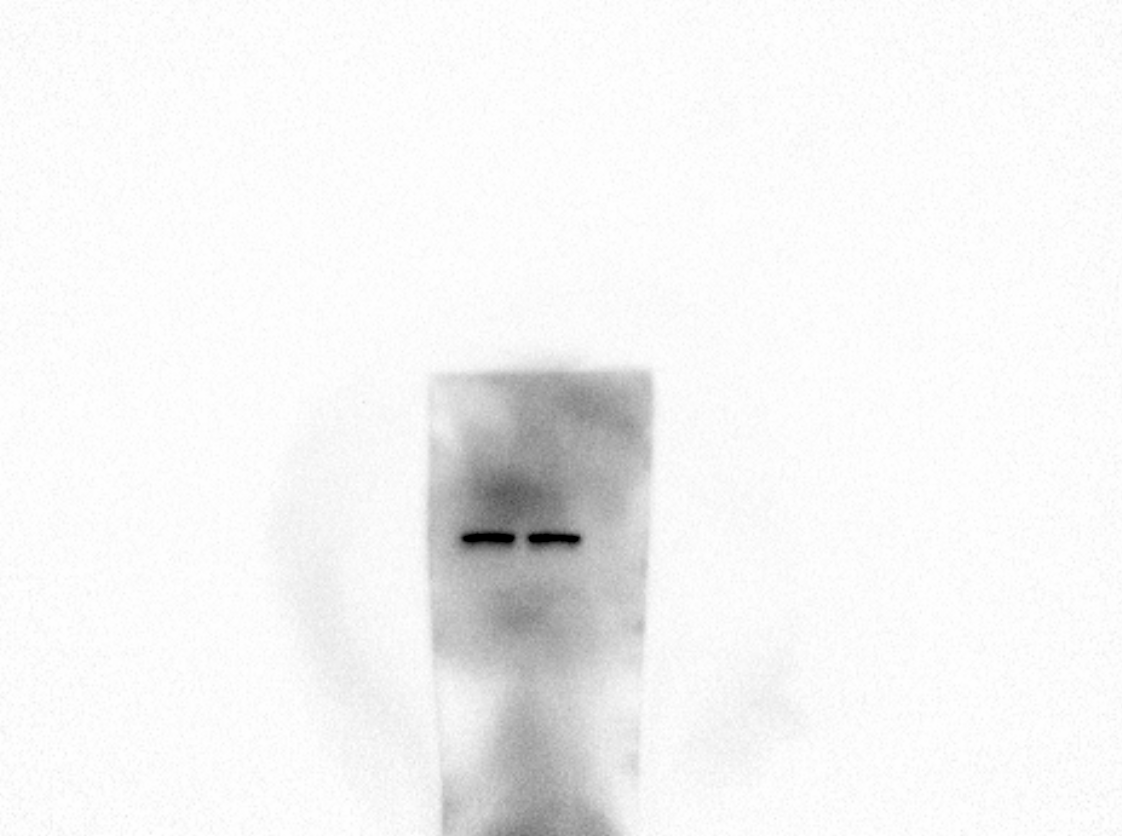

Supplement: Figure 3—source data 1. [file elife-82826-fig3-data1.zip › Figure 3-source data1/Unlabeled Western blot/Figure 3K-GTF3C3.tif]

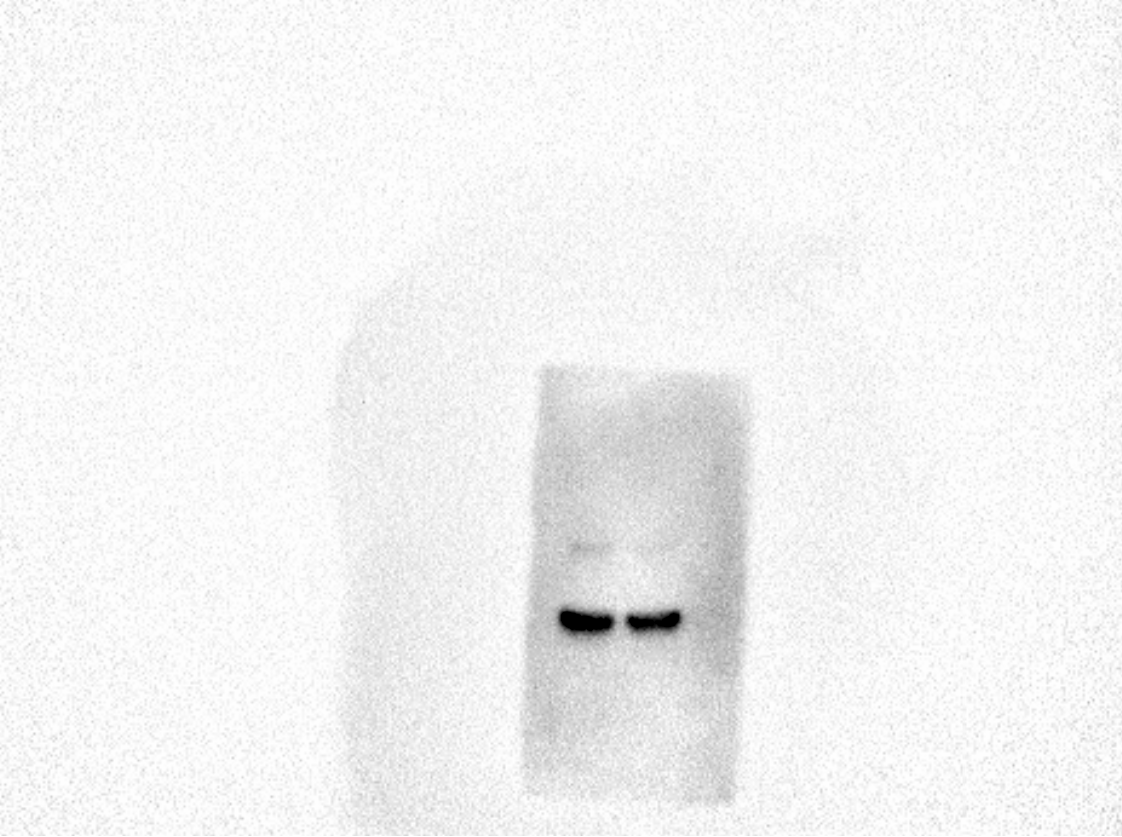

Supplement: Figure 3—source data 1. [file elife-82826-fig3-data1.zip › Figure 3-source data1/Unlabeled Western blot/Figure 3K-TBP.tif]

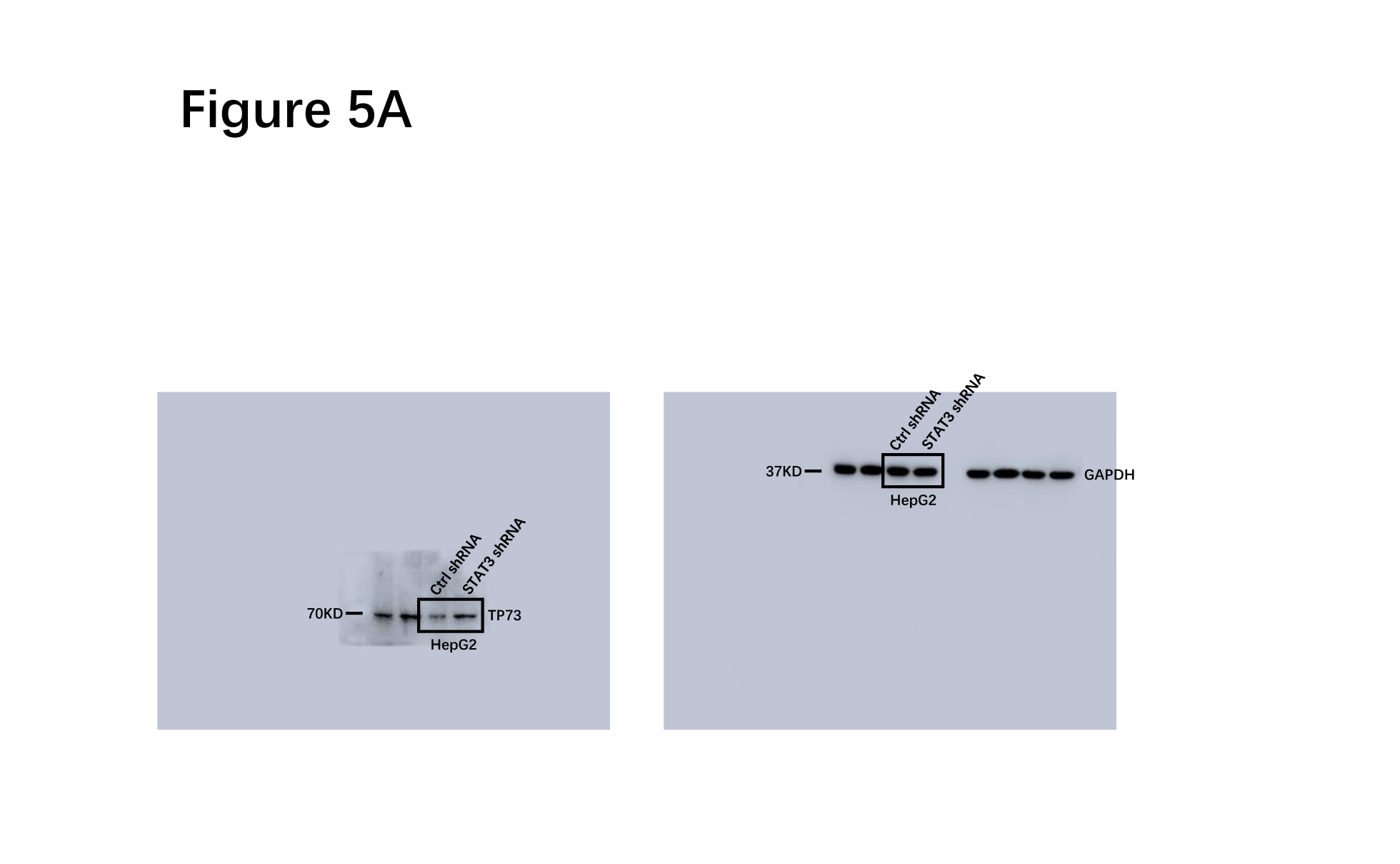

Supplement: Figure 5—source data 1. [file elife-82826-fig5-data1.zip › Figure 5-source data1/Labeled Western blot/Figure 5A.tif]

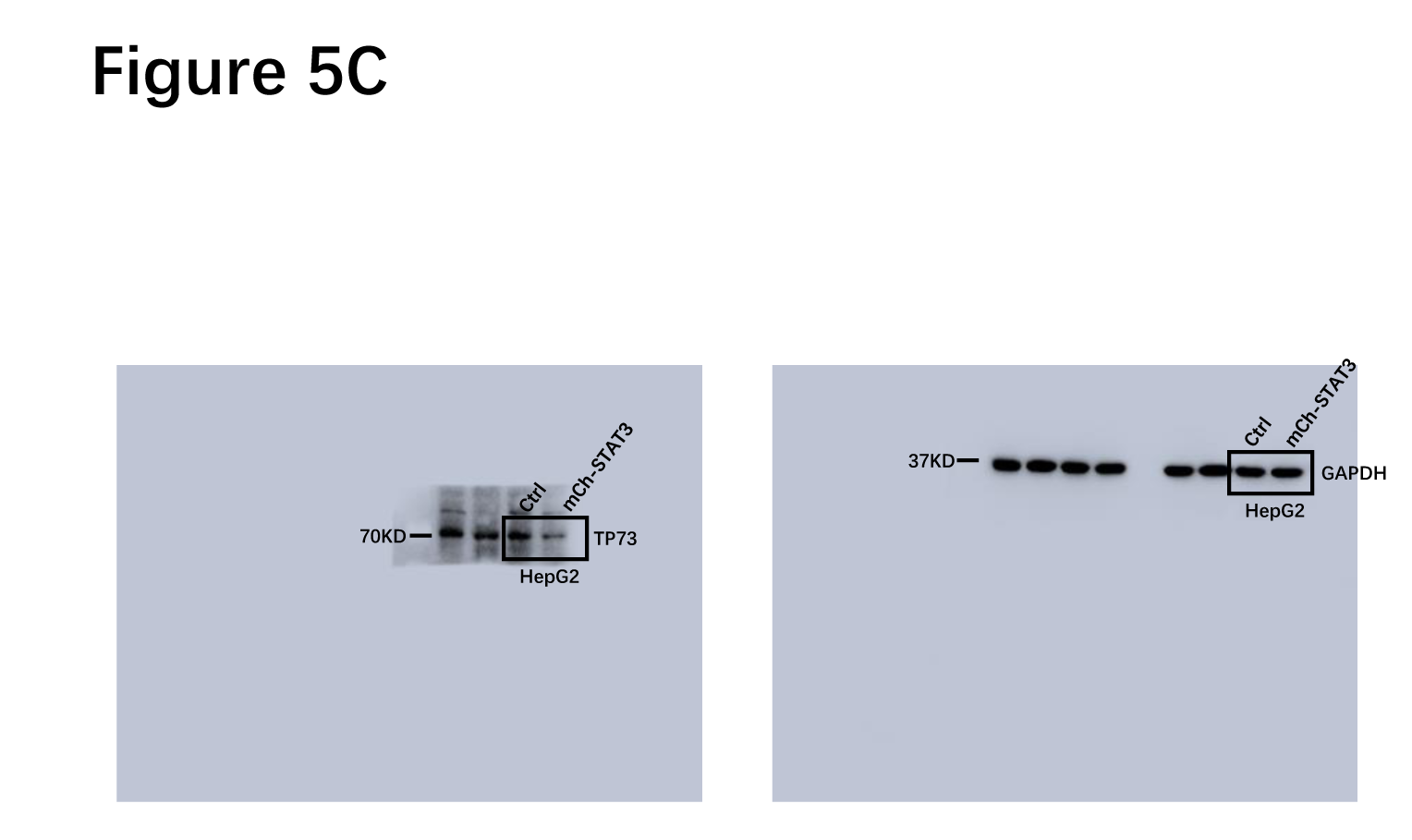

Supplement: Figure 5—source data 1. [file elife-82826-fig5-data1.zip › Figure 5-source data1/Labeled Western blot/Figure 5C.tif]

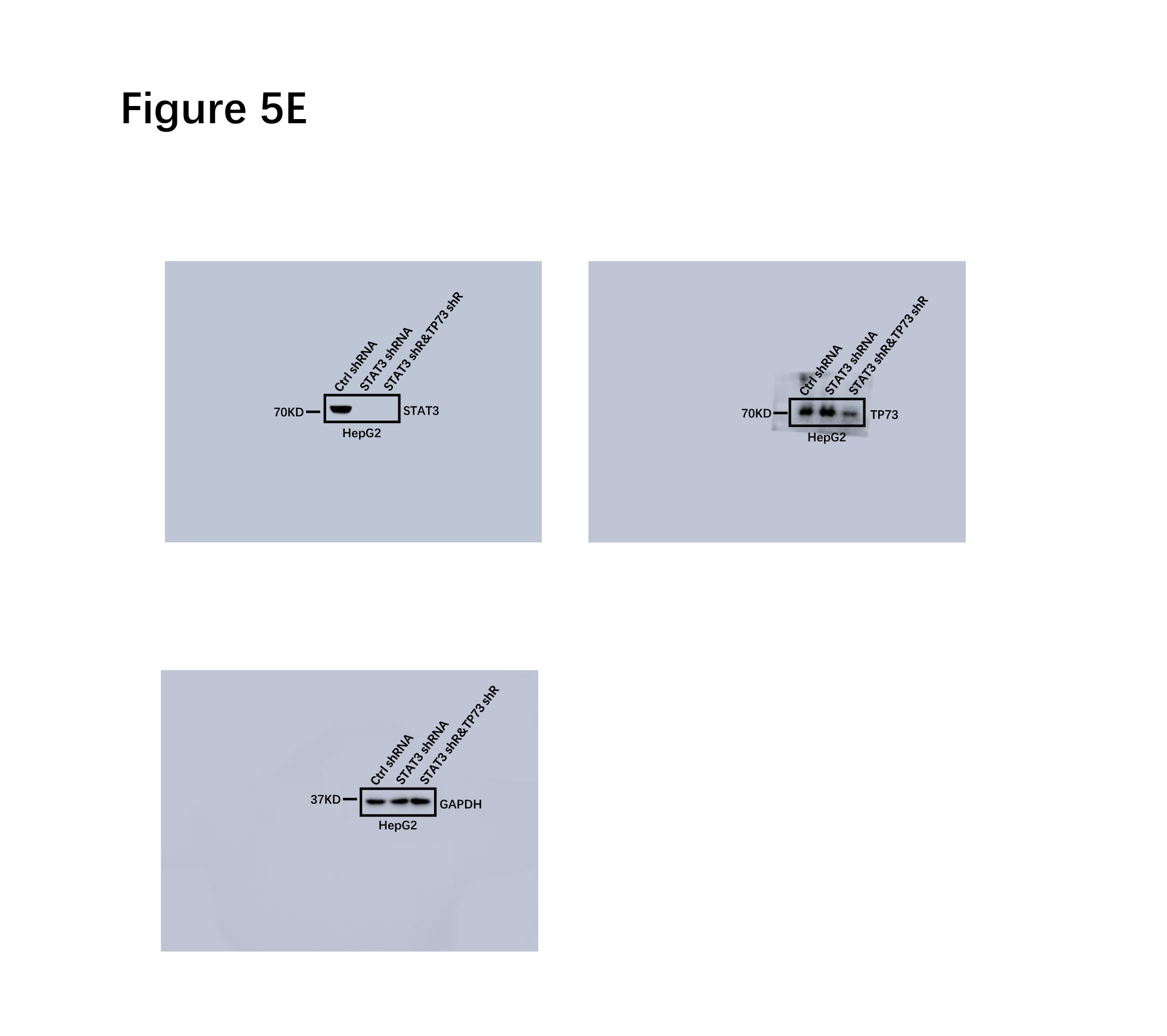

Supplement: Figure 5—source data 1. [file elife-82826-fig5-data1.zip › Figure 5-source data1/Labeled Western blot/Figure 5E.tif]

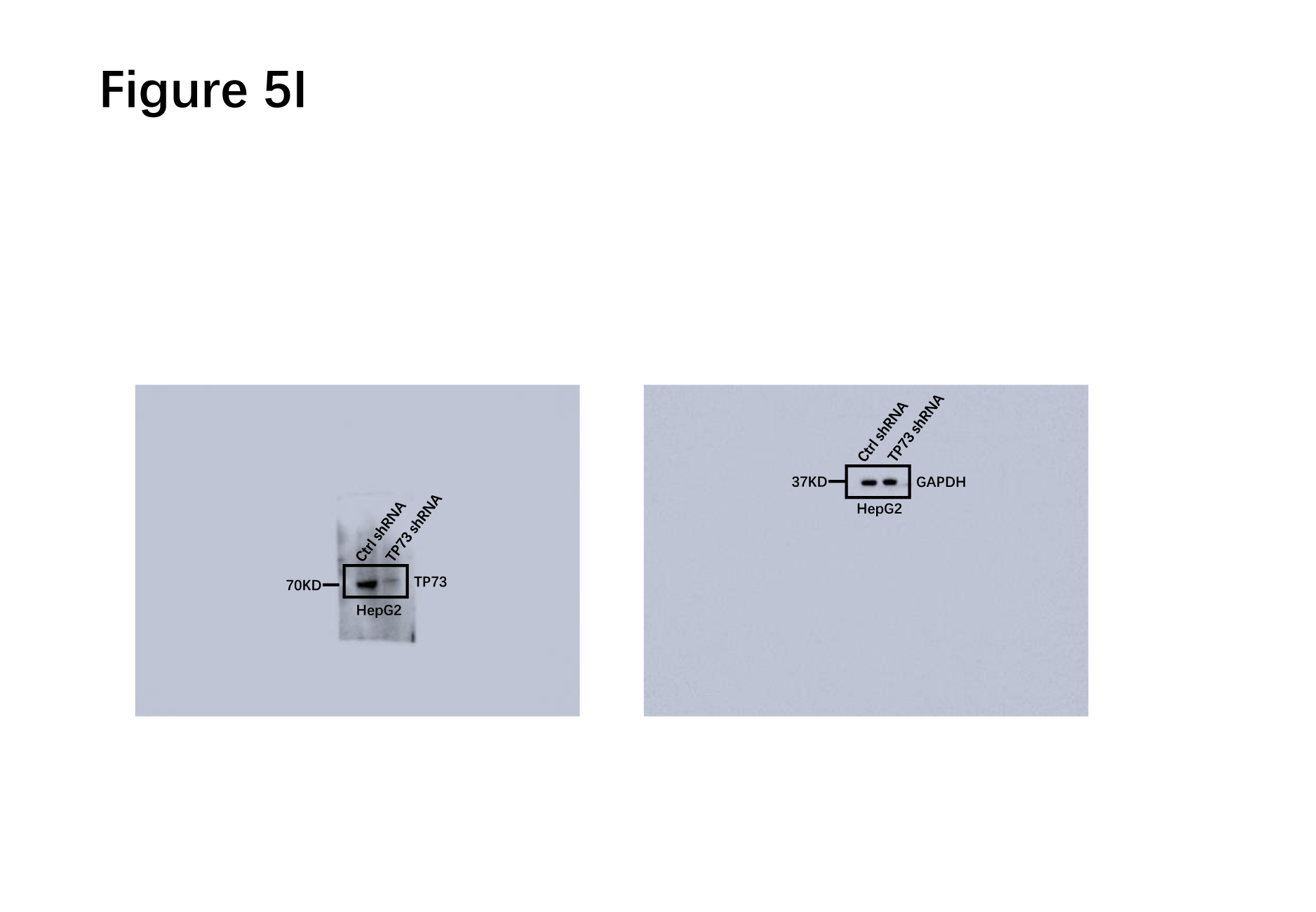

Supplement: Figure 5—source data 1. [file elife-82826-fig5-data1.zip › Figure 5-source data1/Labeled Western blot/Figure 5I.tif]

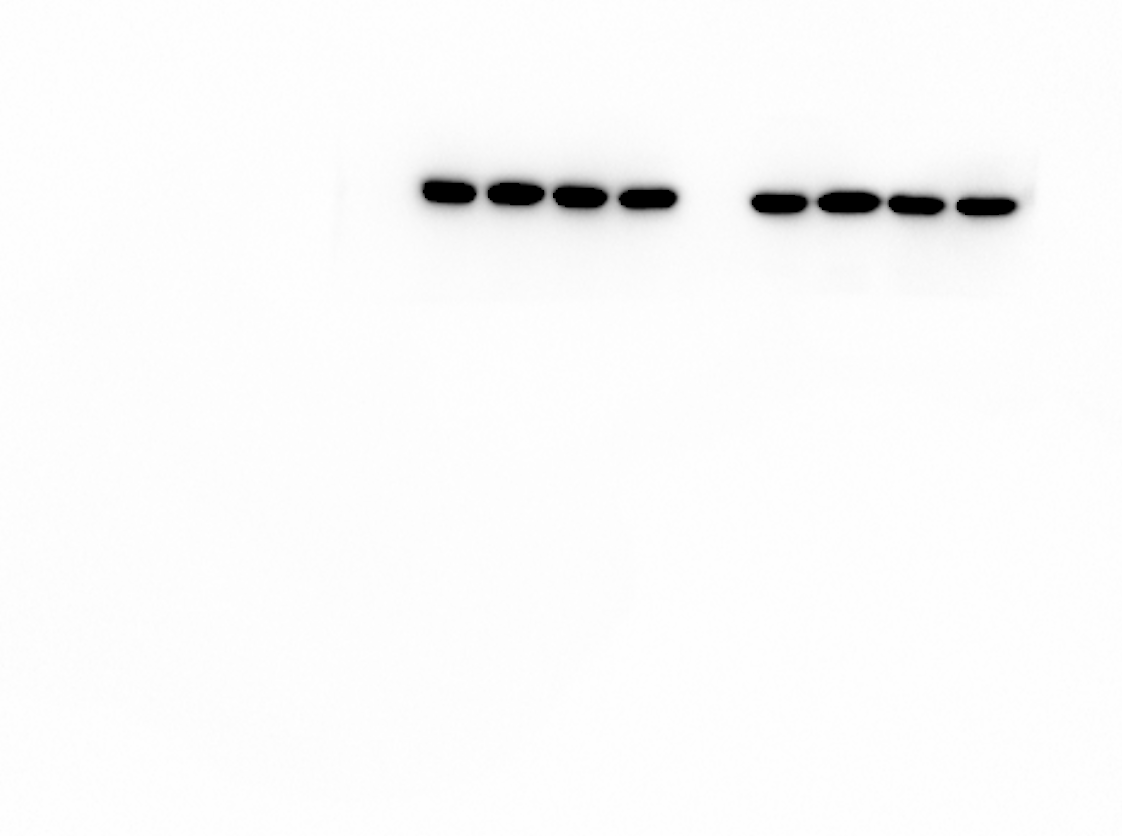

Supplement: Figure 5—source data 1. [file elife-82826-fig5-data1.zip › Figure 5-source data1/Unlabeled Western blot/Figure 5A-GAPDH.tif]

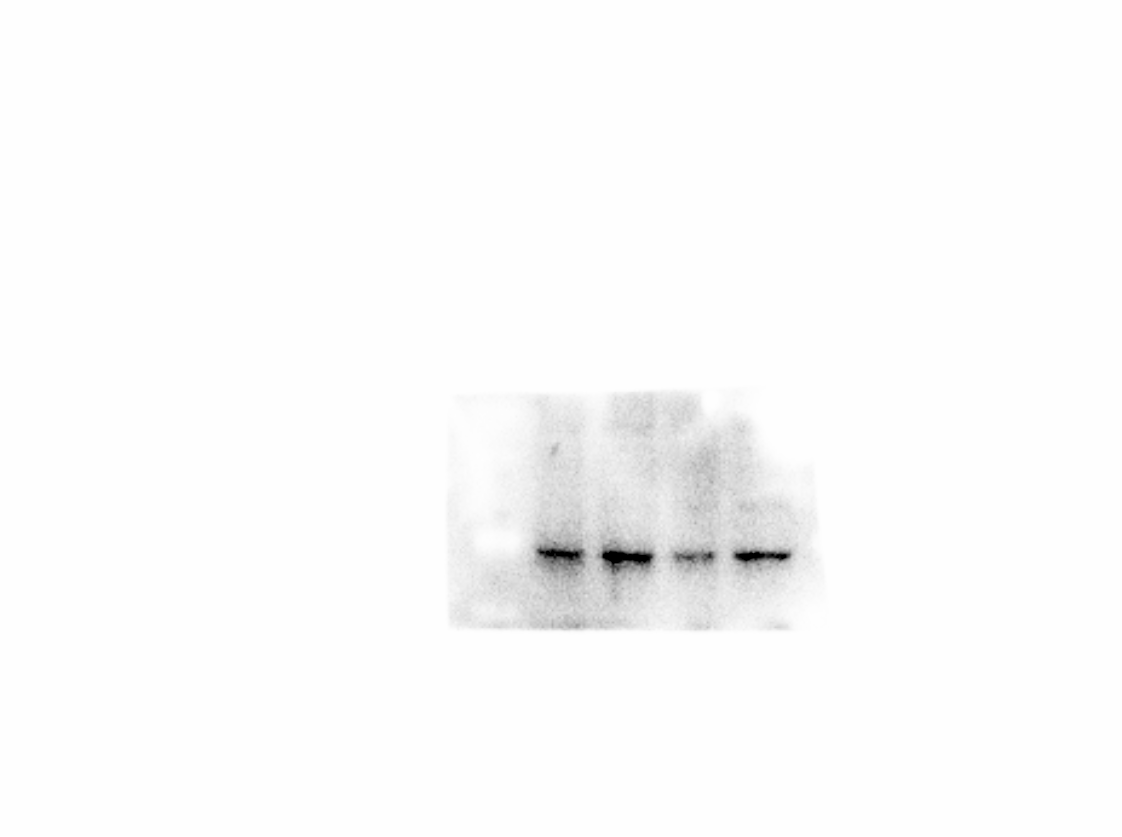

Supplement: Figure 5—source data 1. [file elife-82826-fig5-data1.zip › Figure 5-source data1/Unlabeled Western blot/Figure 5A-TP73.tif]

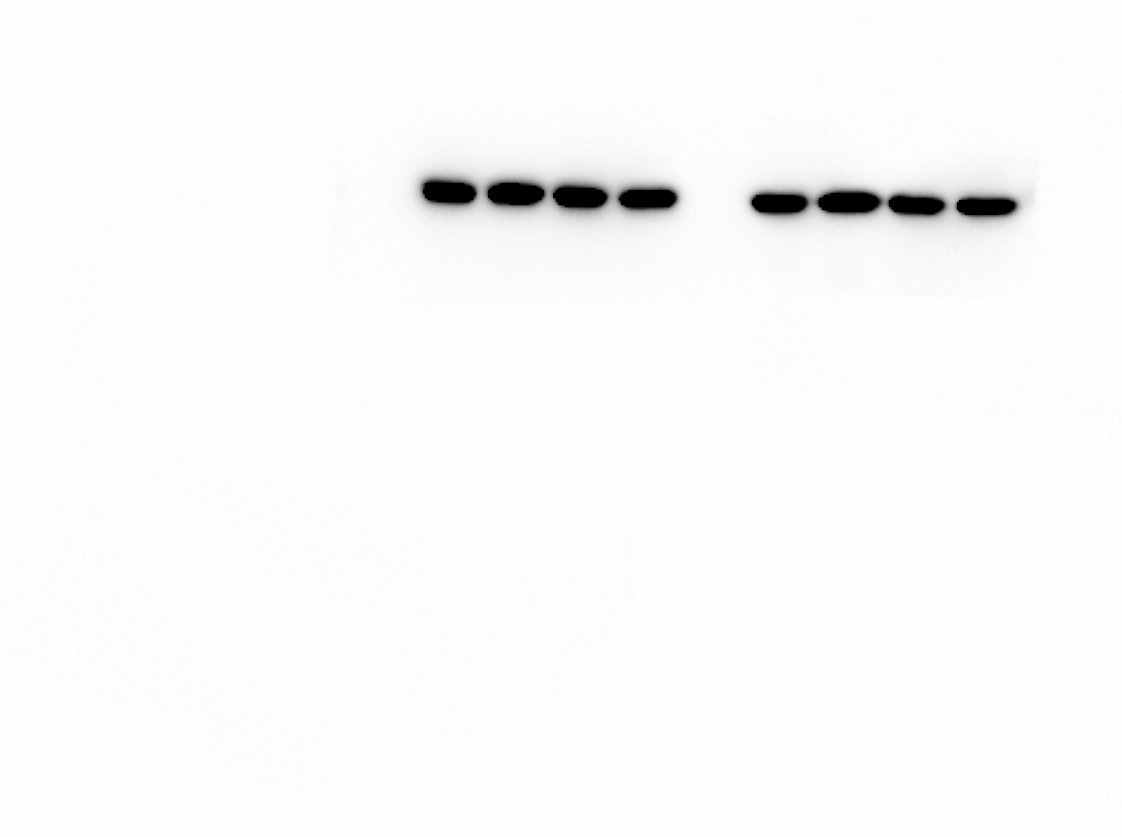

Supplement: Figure 5—source data 1. [file elife-82826-fig5-data1.zip › Figure 5-source data1/Unlabeled Western blot/Figure 5C-GAPDH.tif]

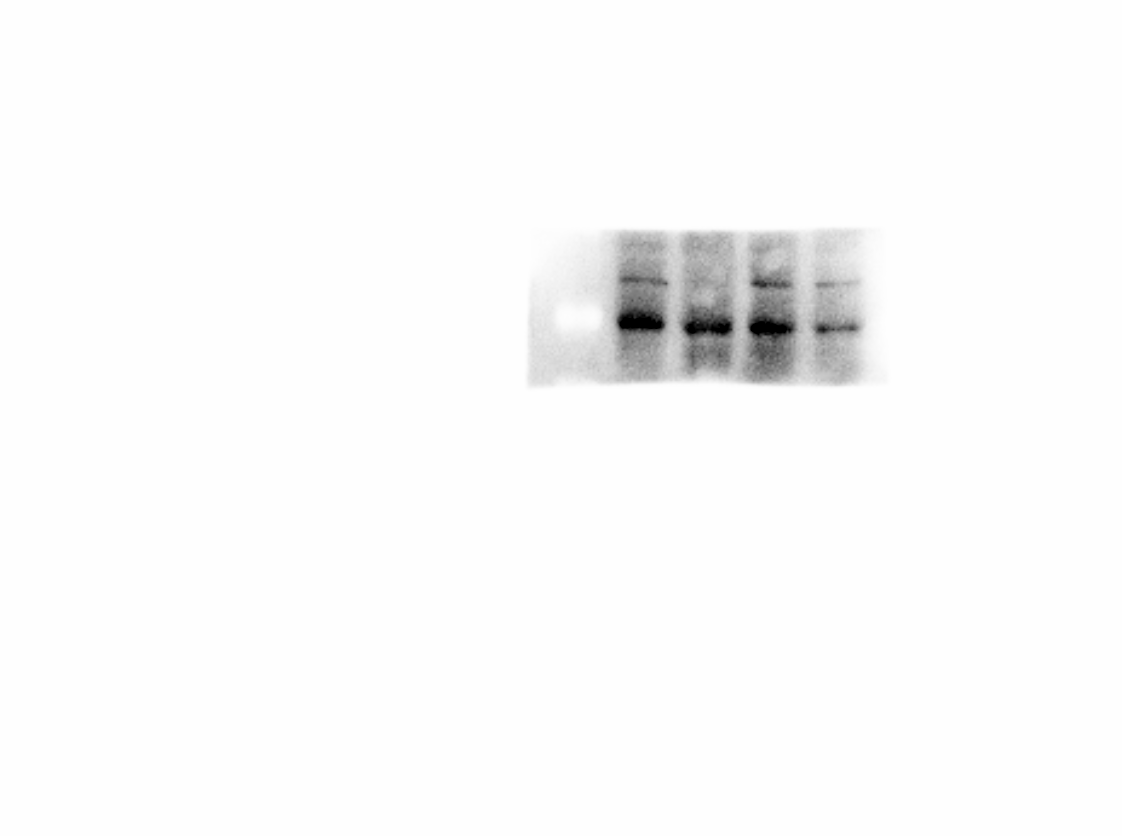

Supplement: Figure 5—source data 1. [file elife-82826-fig5-data1.zip › Figure 5-source data1/Unlabeled Western blot/Figure 5C-TP73.tif]

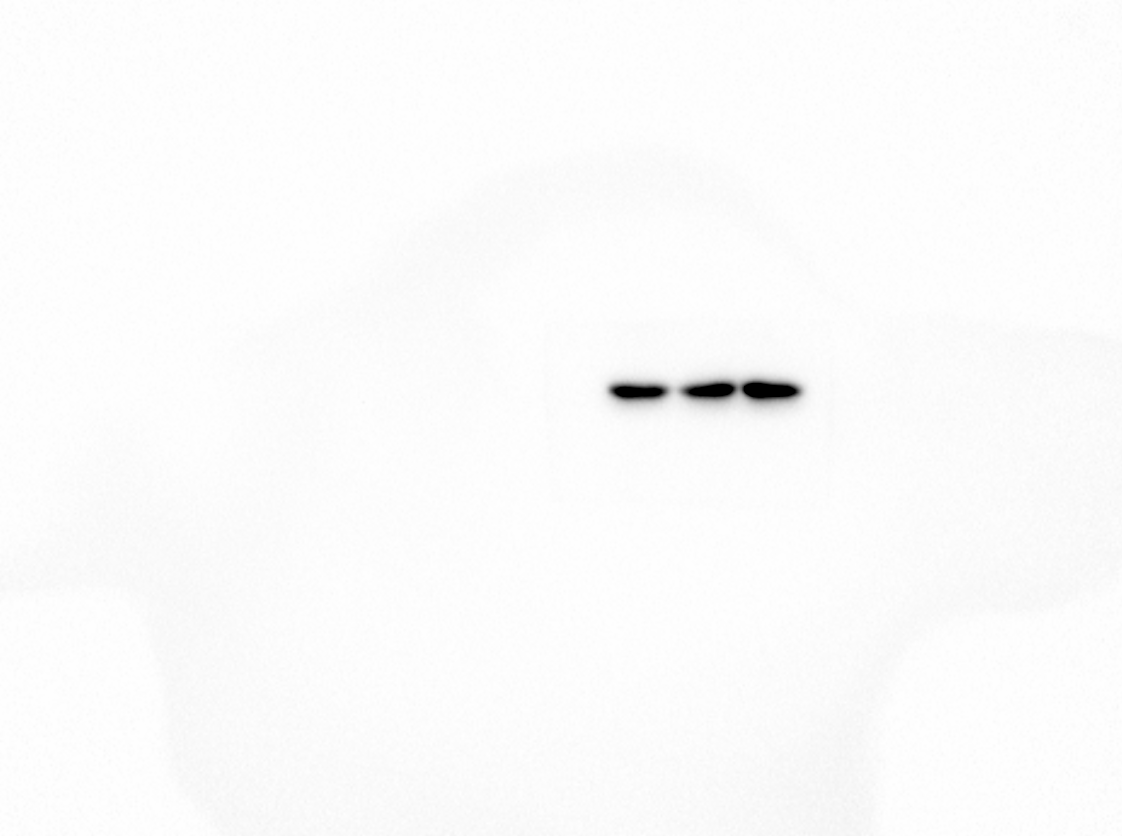

Supplement: Figure 5—source data 1. [file elife-82826-fig5-data1.zip › Figure 5-source data1/Unlabeled Western blot/Figure 5E-GAPDH.tif]

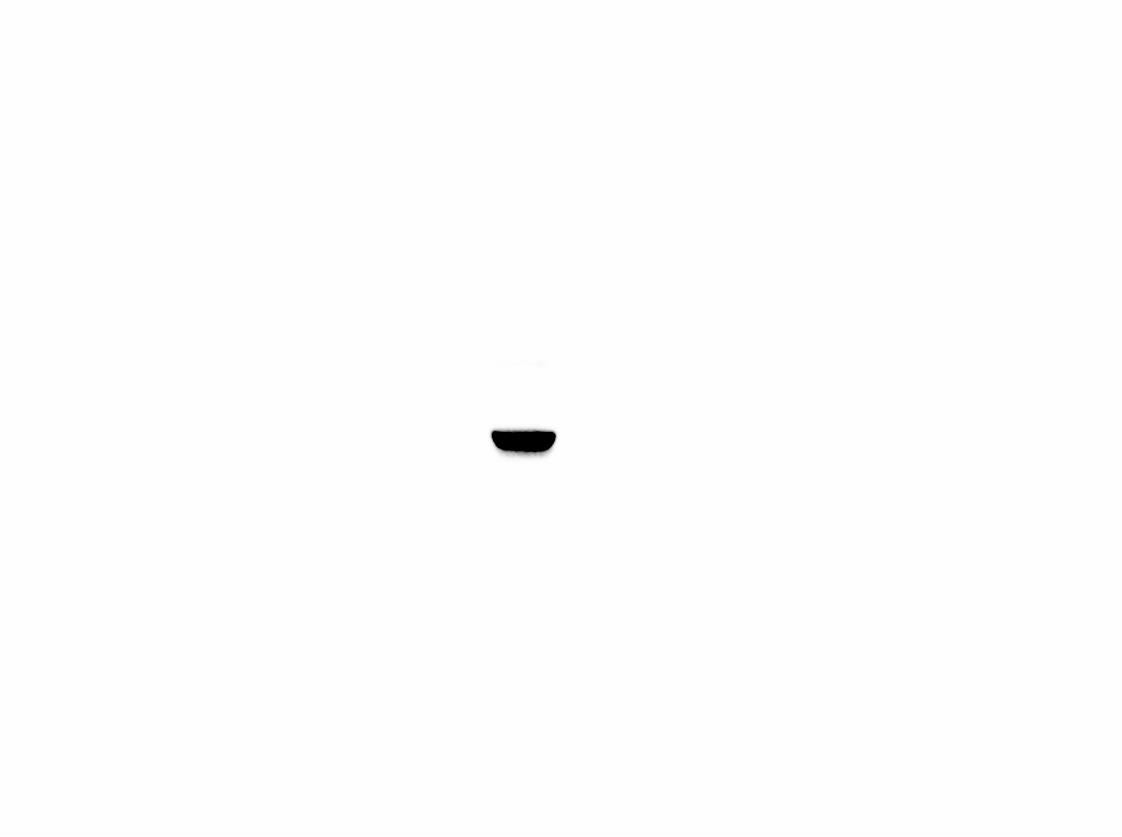

Supplement: Figure 5—source data 1. [file elife-82826-fig5-data1.zip › Figure 5-source data1/Unlabeled Western blot/Figure 5E-STAT3.tif]

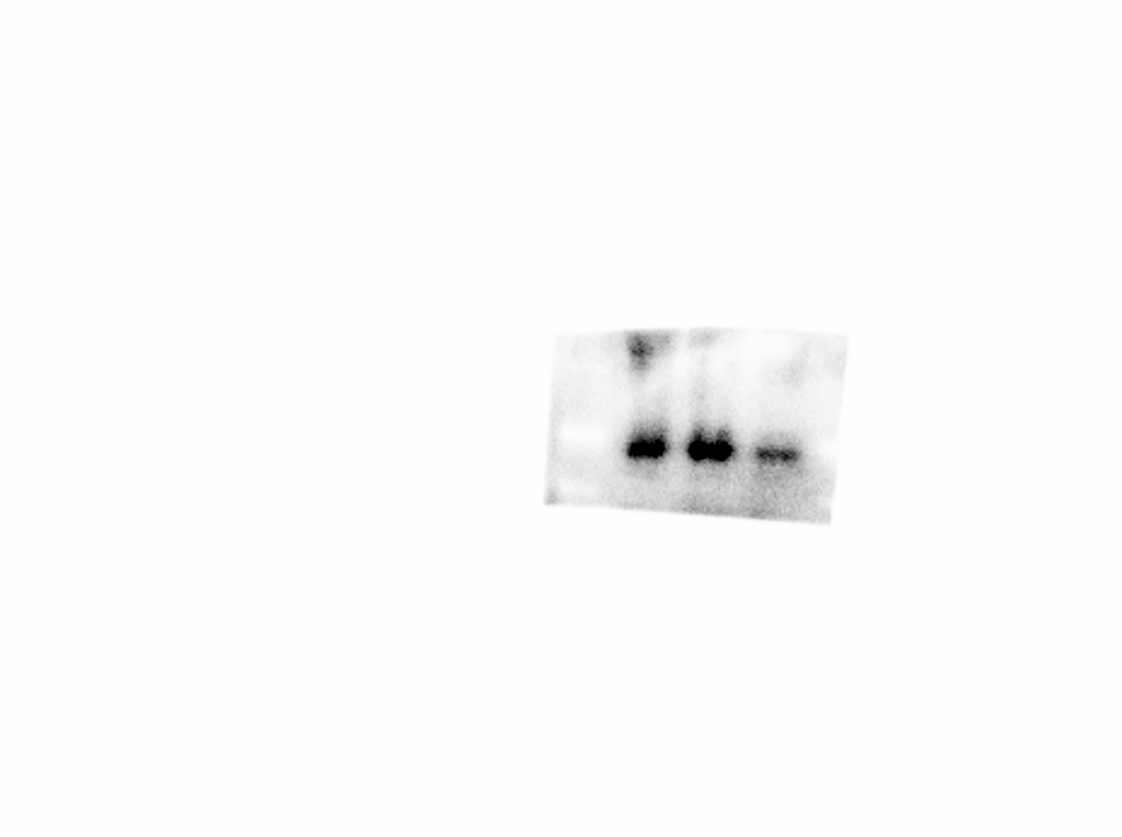

Supplement: Figure 5—source data 1. [file elife-82826-fig5-data1.zip › Figure 5-source data1/Unlabeled Western blot/Figure 5E-TP73.tif]

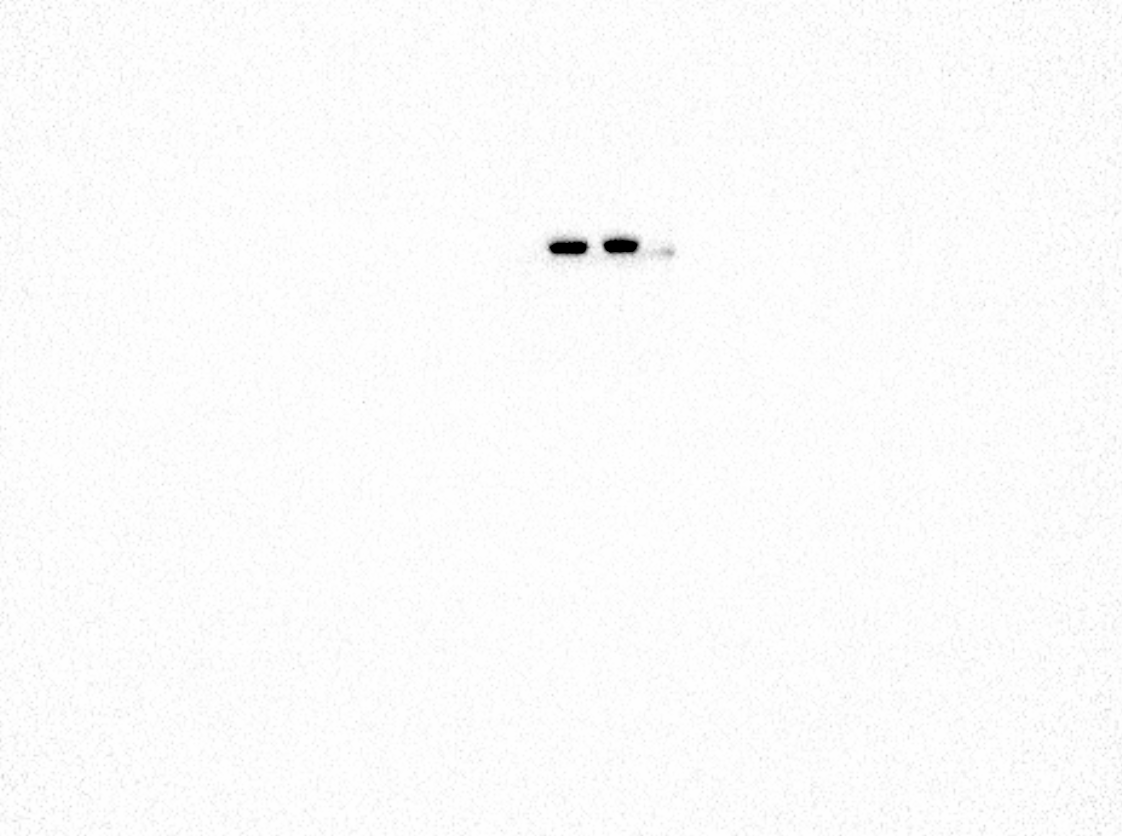

Supplement: Figure 5—source data 1. [file elife-82826-fig5-data1.zip › Figure 5-source data1/Unlabeled Western blot/I-GAPDH.tif]

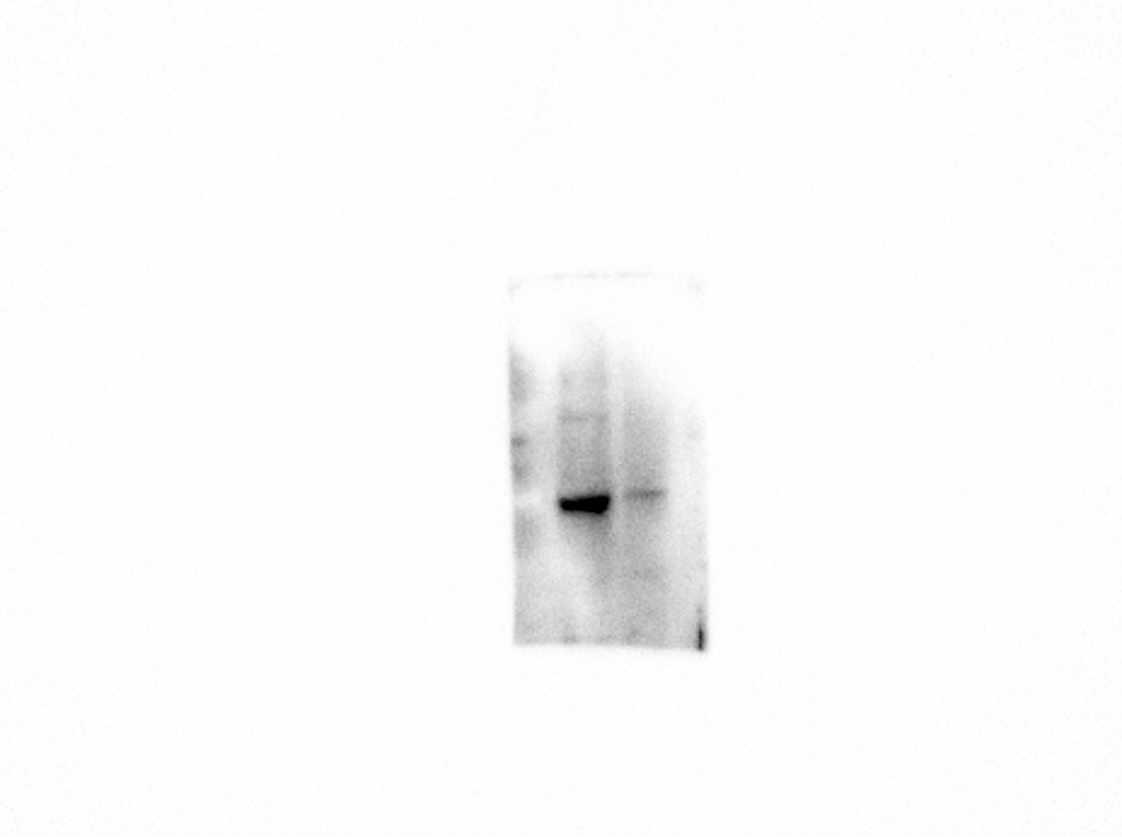

Supplement: Figure 5—source data 1. [file elife-82826-fig5-data1.zip › Figure 5-source data1/Unlabeled Western blot/I-TP73.tif]

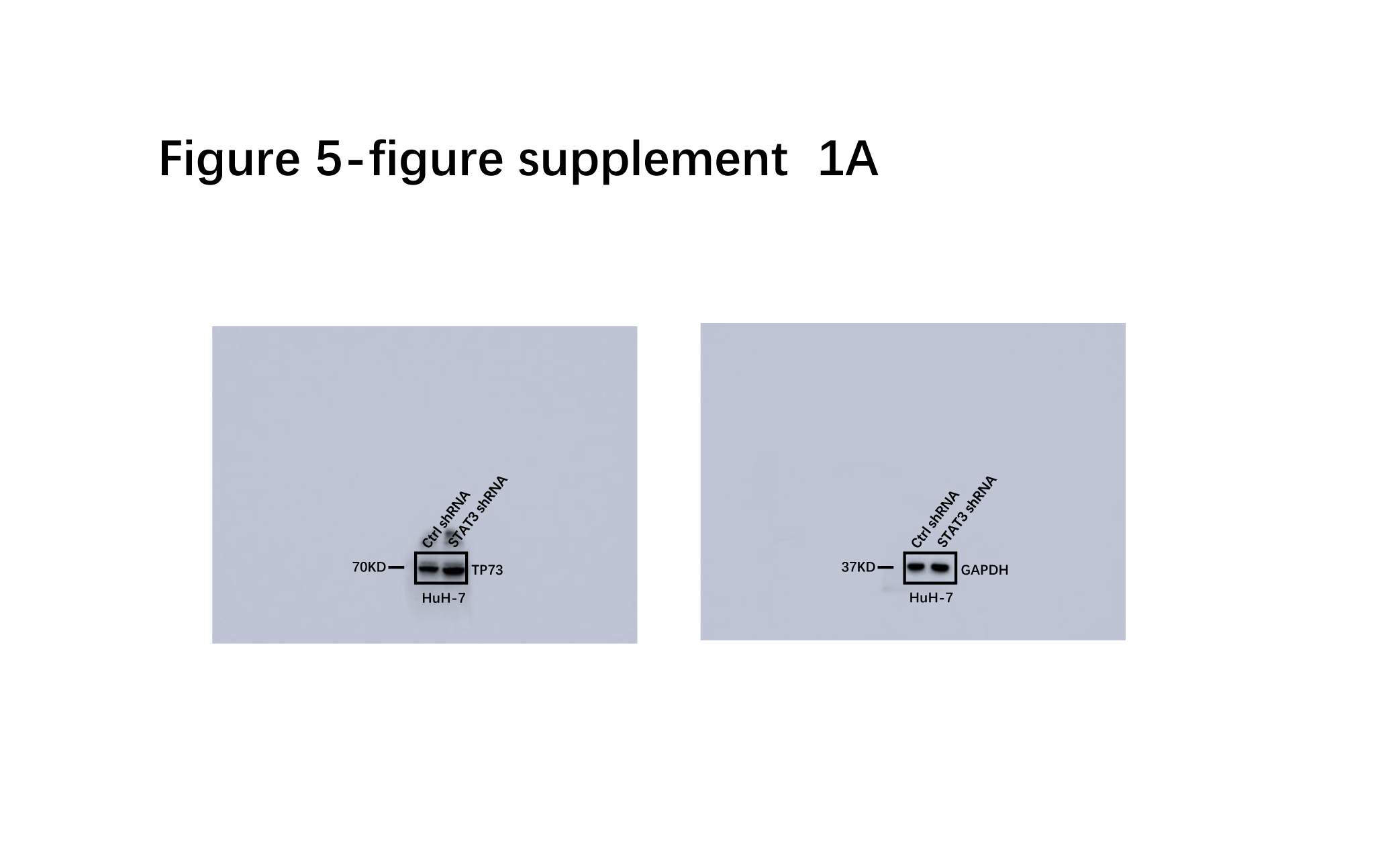

Supplement: Figure 5—figure supplement 1—source data 1. [file elife-82826-fig5-figsupp1-data1.zip › Figure 5-figure supplement 1-source data 1/Labeled Western blot/Figure 5-figure supplement 1A.tif]

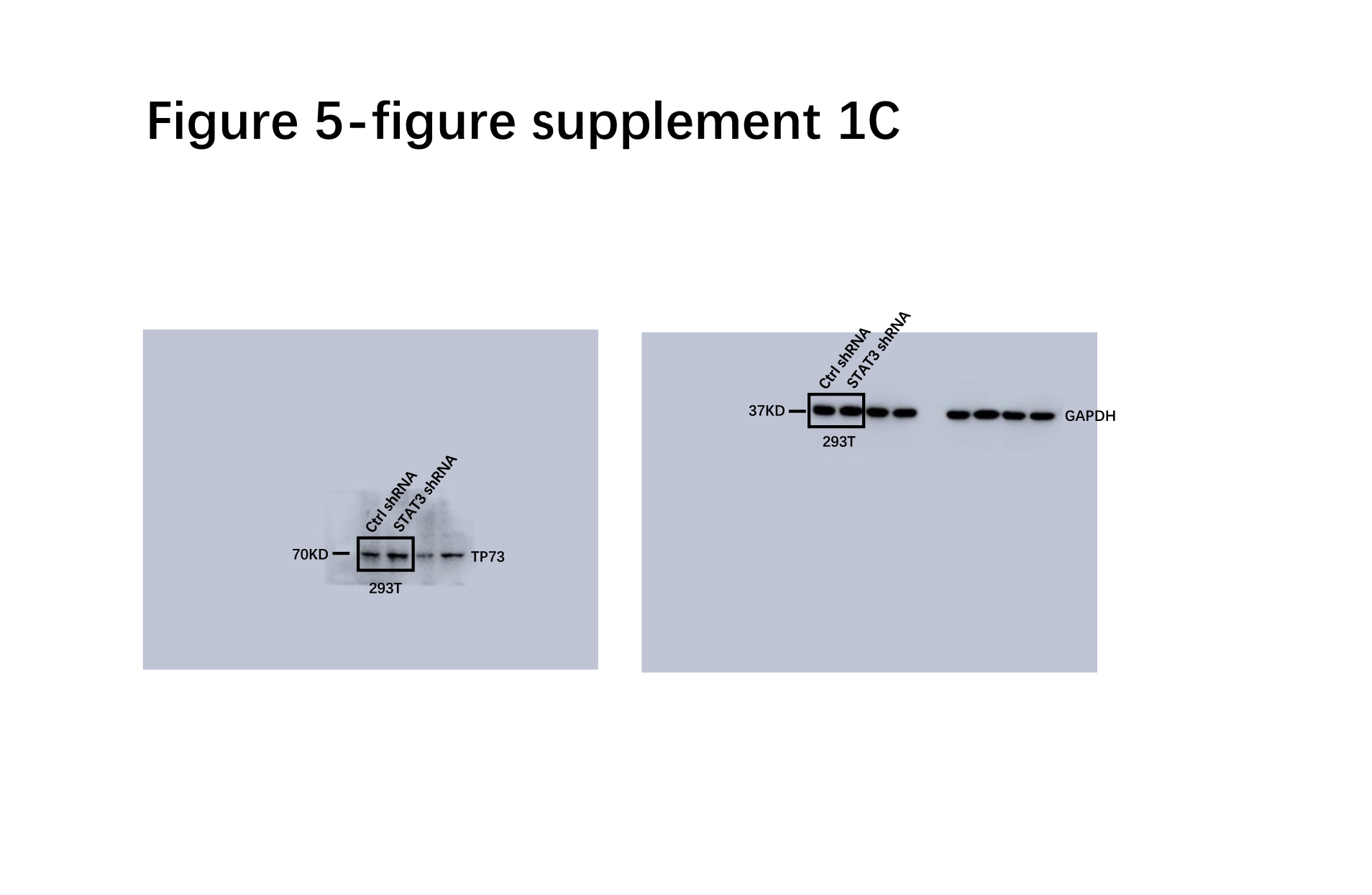

Supplement: Figure 5—figure supplement 1—source data 1. [file elife-82826-fig5-figsupp1-data1.zip › Figure 5-figure supplement 1-source data 1/Labeled Western blot/Figure 5-figure supplement 1C.tif]

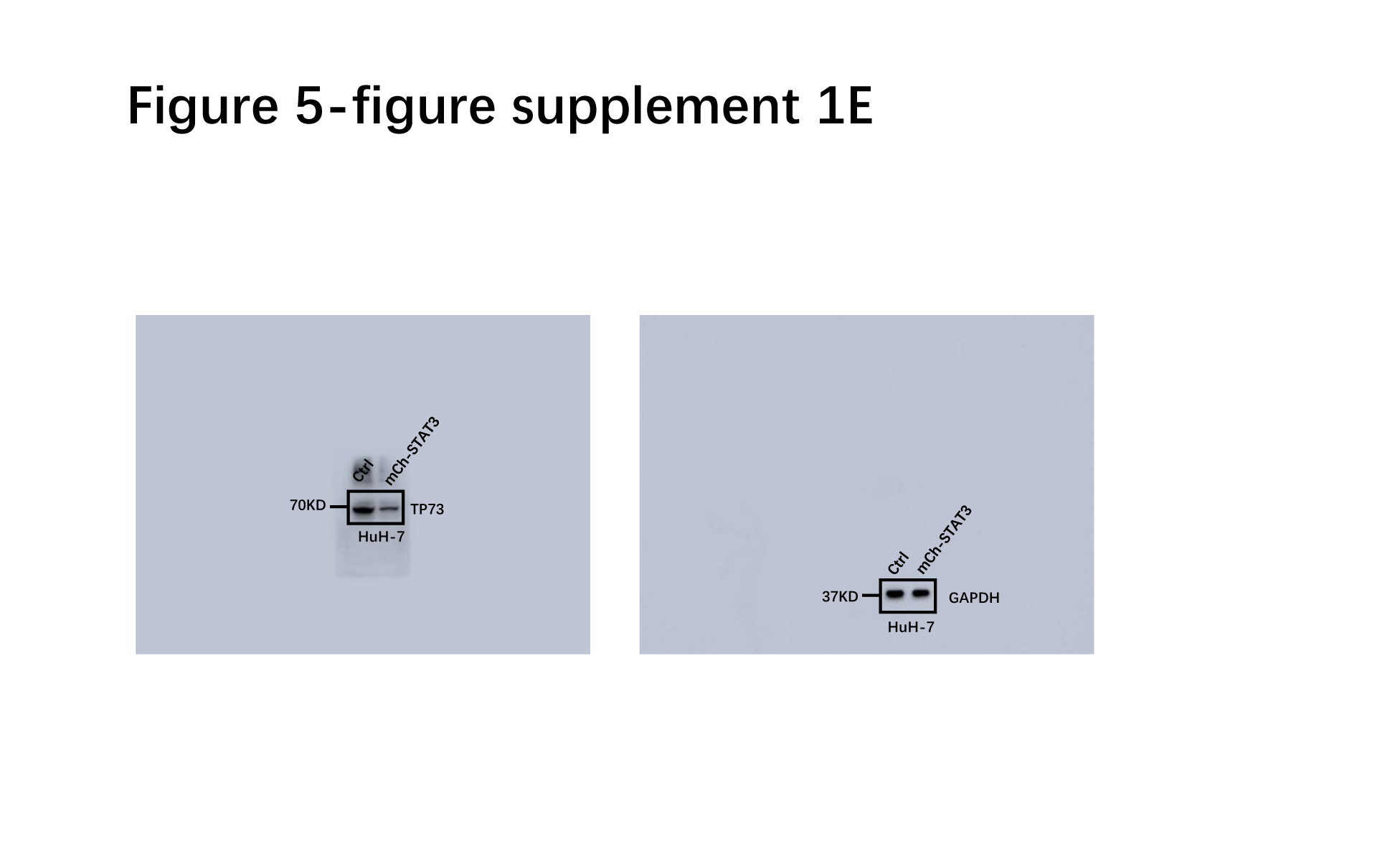

Supplement: Figure 5—figure supplement 1—source data 1. [file elife-82826-fig5-figsupp1-data1.zip › Figure 5-figure supplement 1-source data 1/Labeled Western blot/Figure 5-figure supplement 1E.tif]

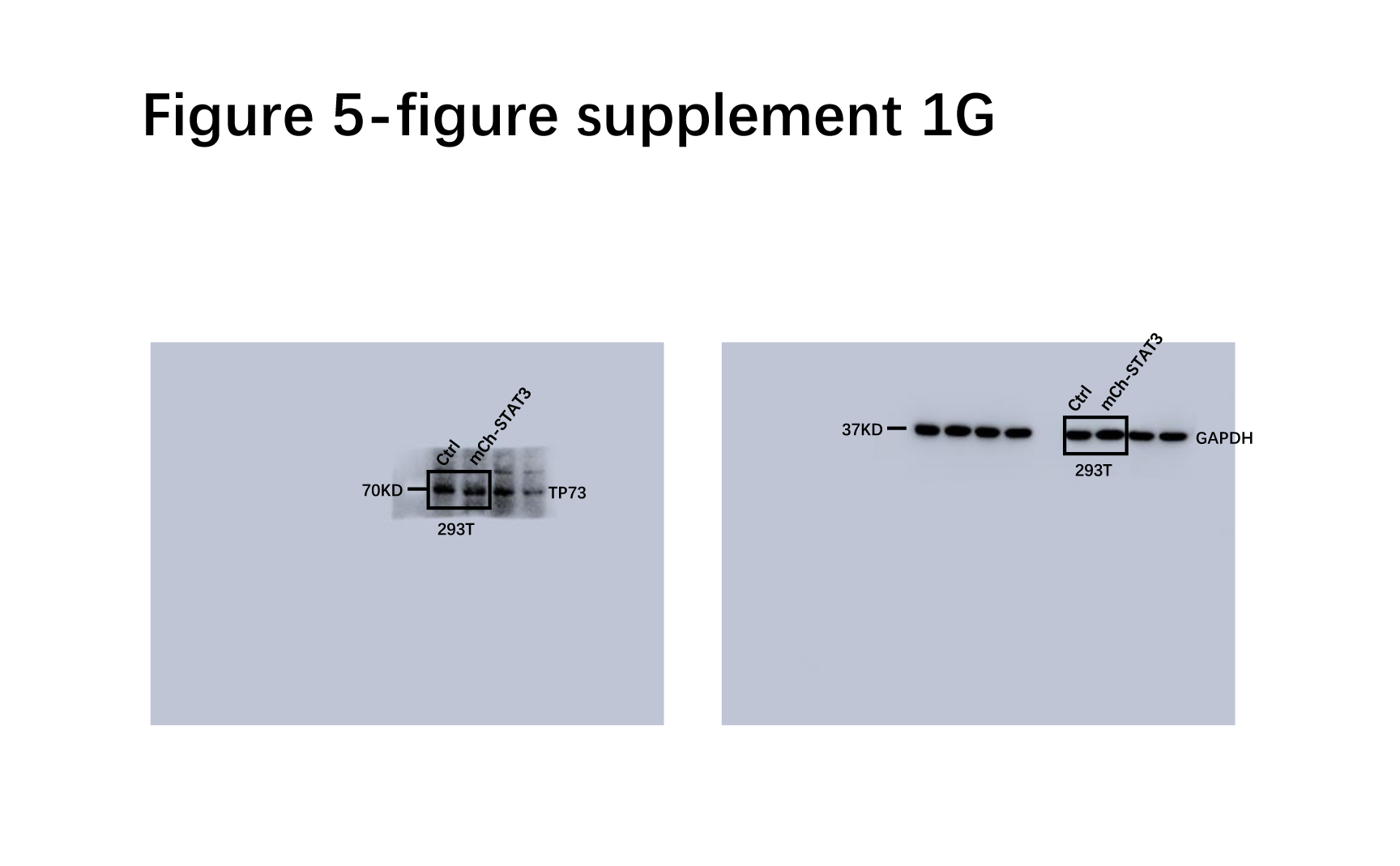

Supplement: Figure 5—figure supplement 1—source data 1. [file elife-82826-fig5-figsupp1-data1.zip › Figure 5-figure supplement 1-source data 1/Labeled Western blot/Figure 5-figure supplement 1G.tif]

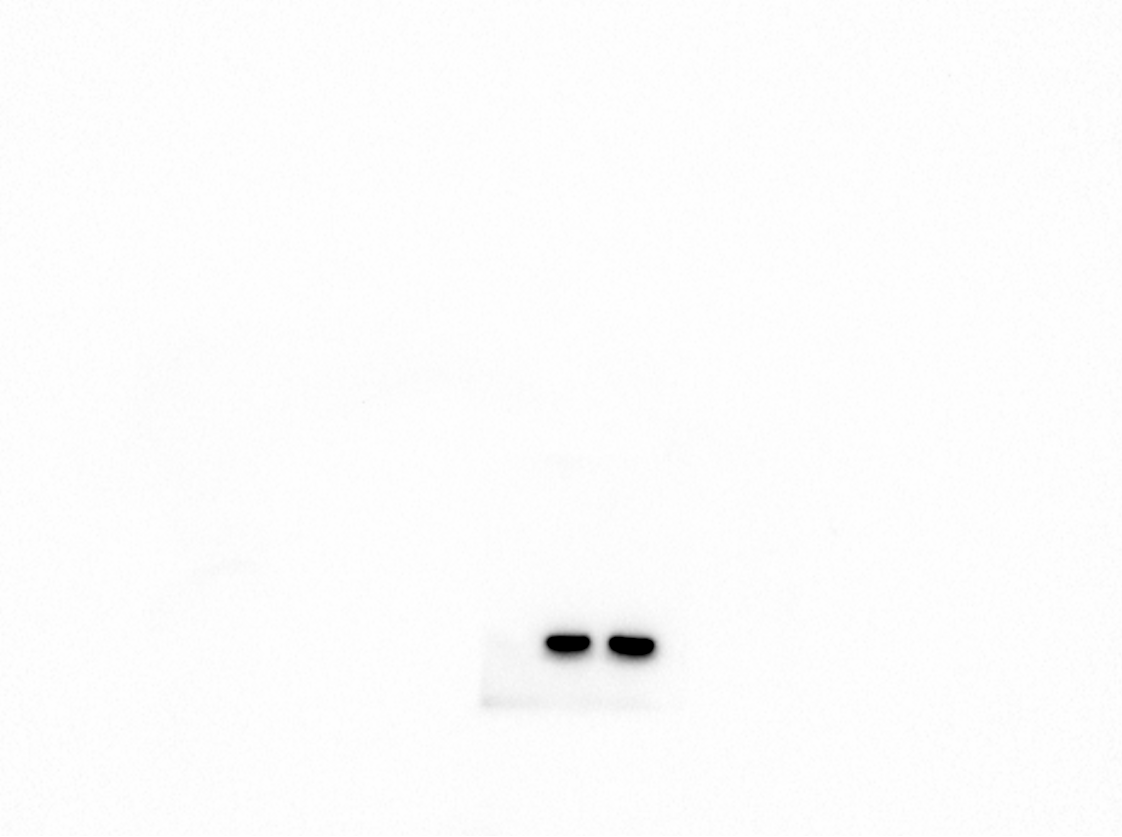

Supplement: Figure 5—figure supplement 1—source data 1. [file elife-82826-fig5-figsupp1-data1.zip › Figure 5-figure supplement 1-source data 1/Unlabeled Western blot/Fig 5-fig sup 1A-GAPDH.tif]

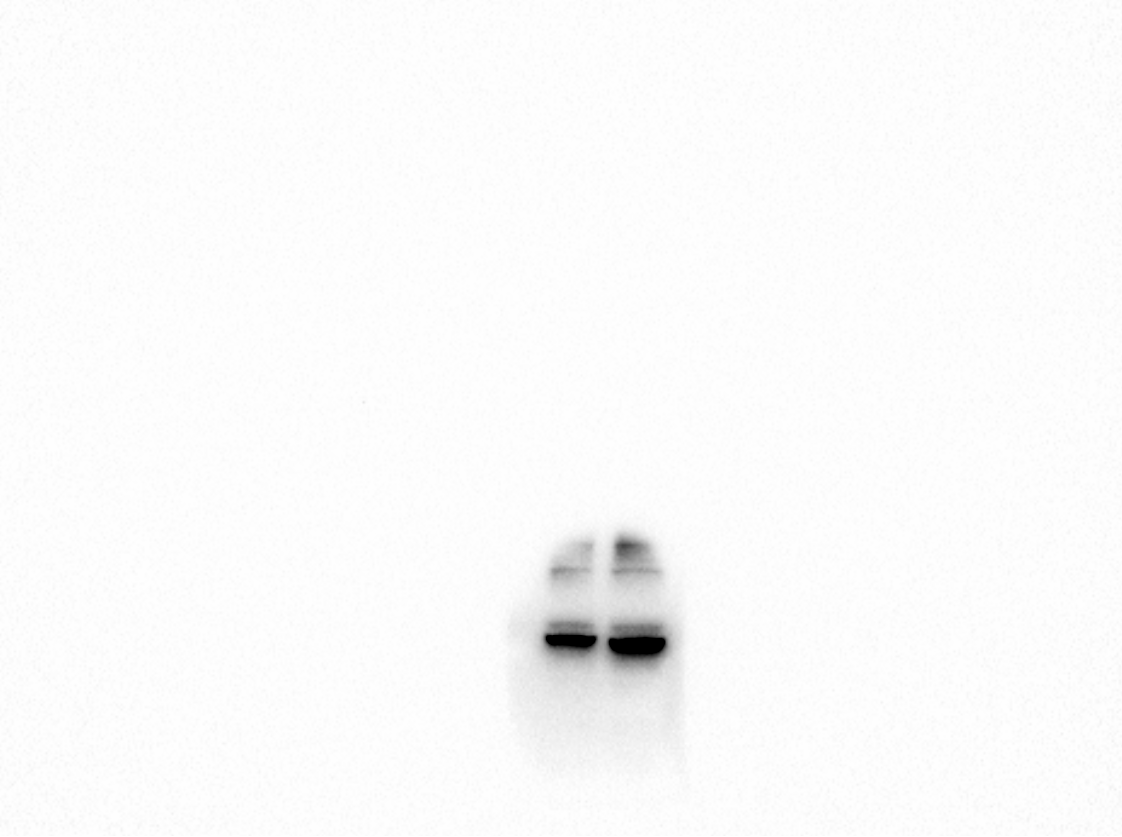

Supplement: Figure 5—figure supplement 1—source data 1. [file elife-82826-fig5-figsupp1-data1.zip › Figure 5-figure supplement 1-source data 1/Unlabeled Western blot/Fig 5-fig sup 1A-TP73.tif]

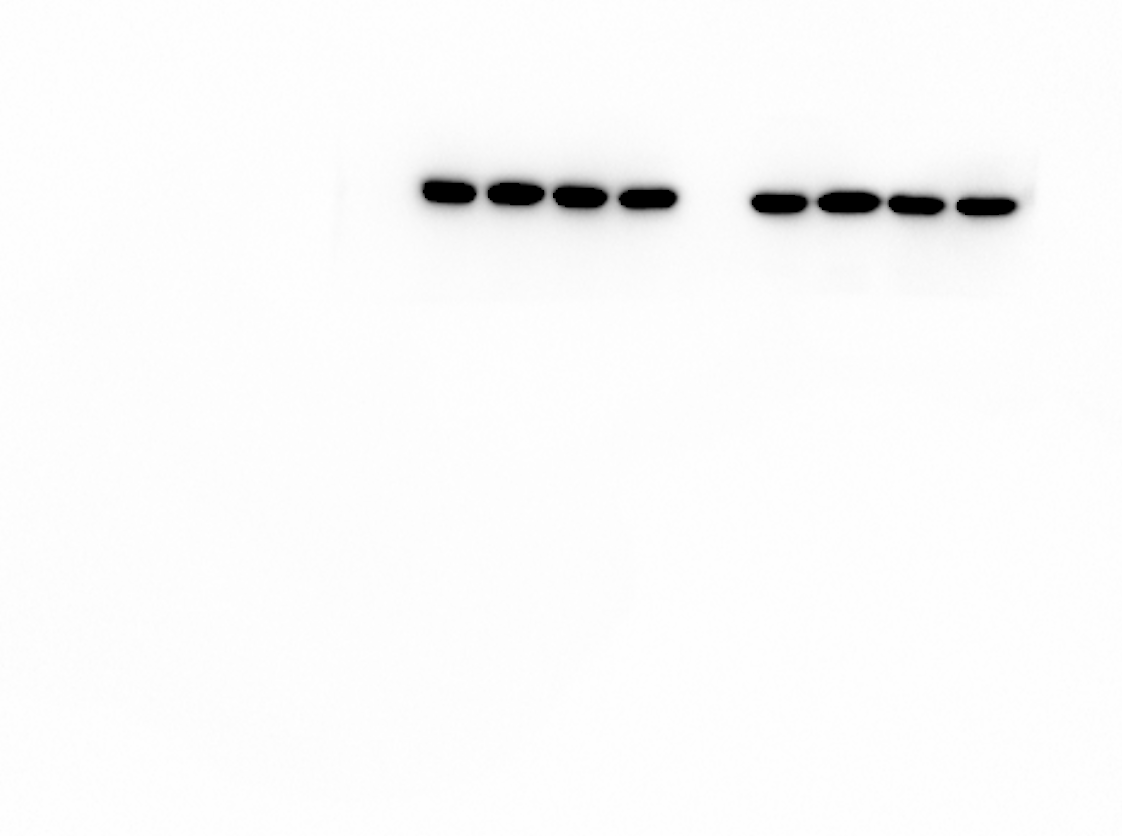

Supplement: Figure 5—figure supplement 1—source data 1. [file elife-82826-fig5-figsupp1-data1.zip › Figure 5-figure supplement 1-source data 1/Unlabeled Western blot/Fig 5-fig sup 1C-GAPDH.tif]

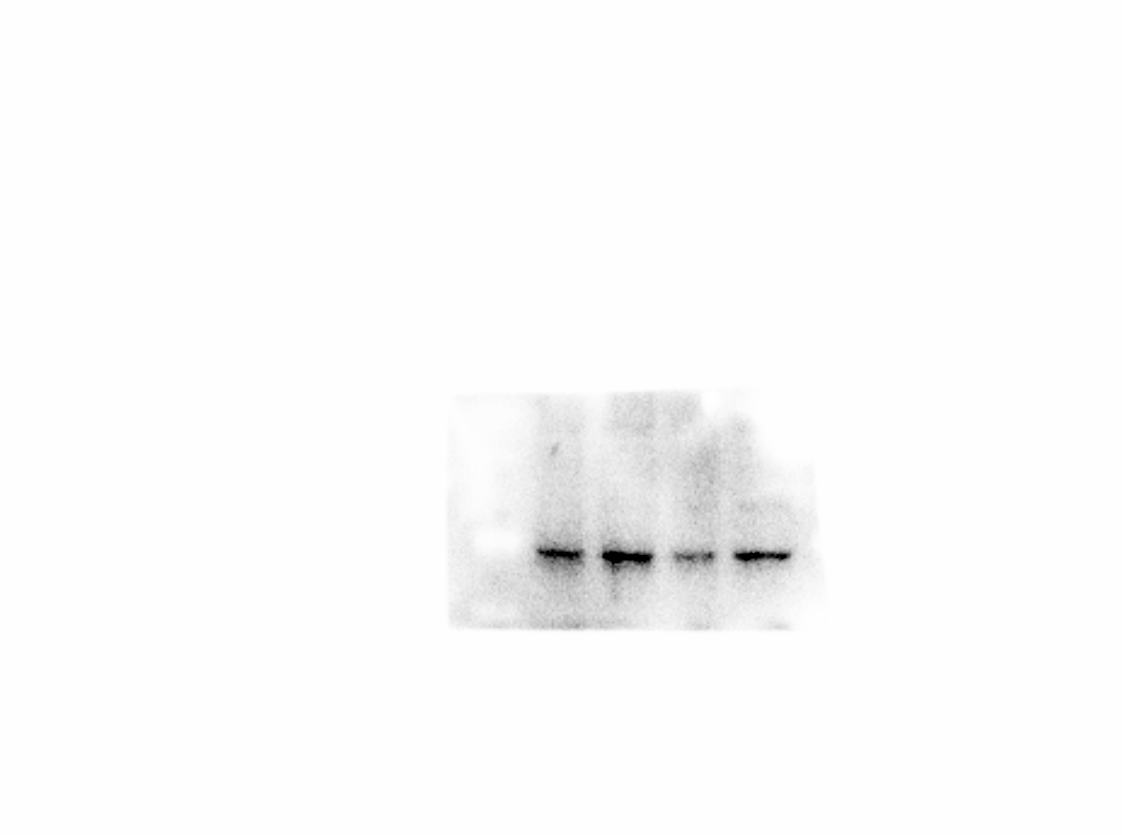

Supplement: Figure 5—figure supplement 1—source data 1. [file elife-82826-fig5-figsupp1-data1.zip › Figure 5-figure supplement 1-source data 1/Unlabeled Western blot/Fig 5-fig sup 1C-TP73.tif]

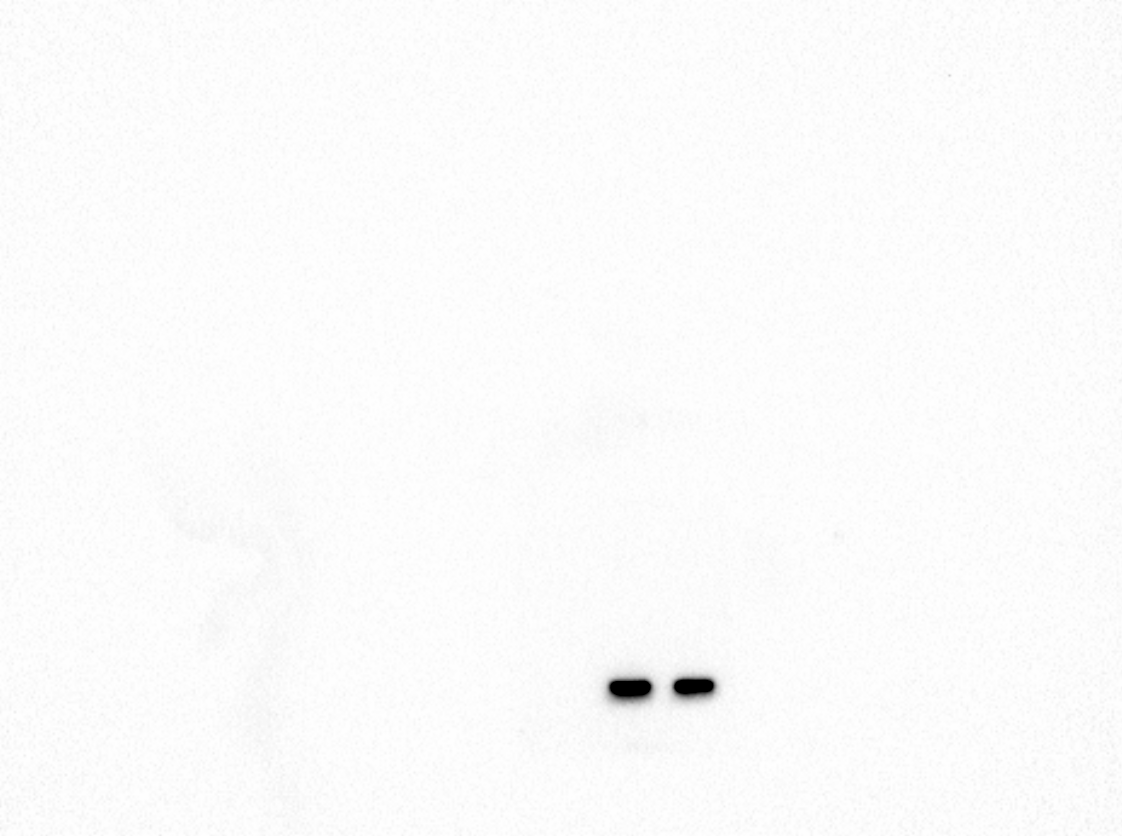

Supplement: Figure 5—figure supplement 1—source data 1. [file elife-82826-fig5-figsupp1-data1.zip › Figure 5-figure supplement 1-source data 1/Unlabeled Western blot/Fig 5-fig sup 1E-GAPDH.tif]

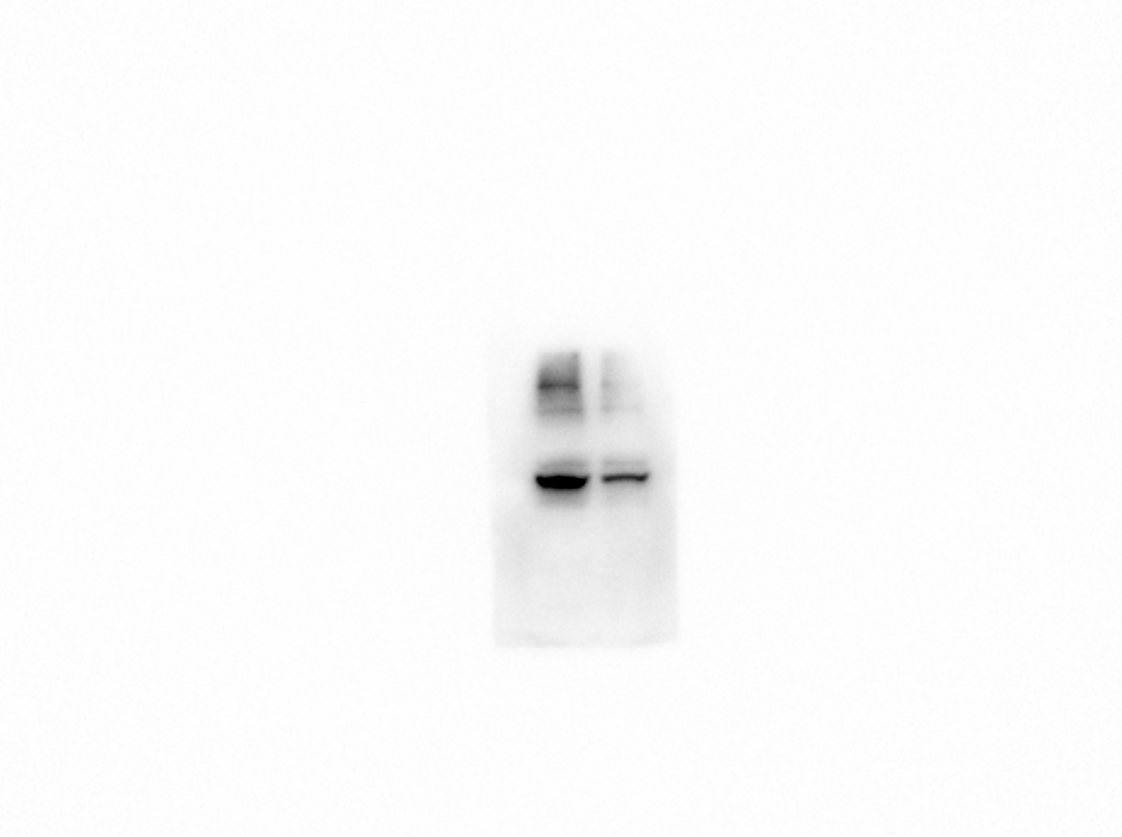

Supplement: Figure 5—figure supplement 1—source data 1. [file elife-82826-fig5-figsupp1-data1.zip › Figure 5-figure supplement 1-source data 1/Unlabeled Western blot/Fig 5-fig sup 1E-TP73.tif]

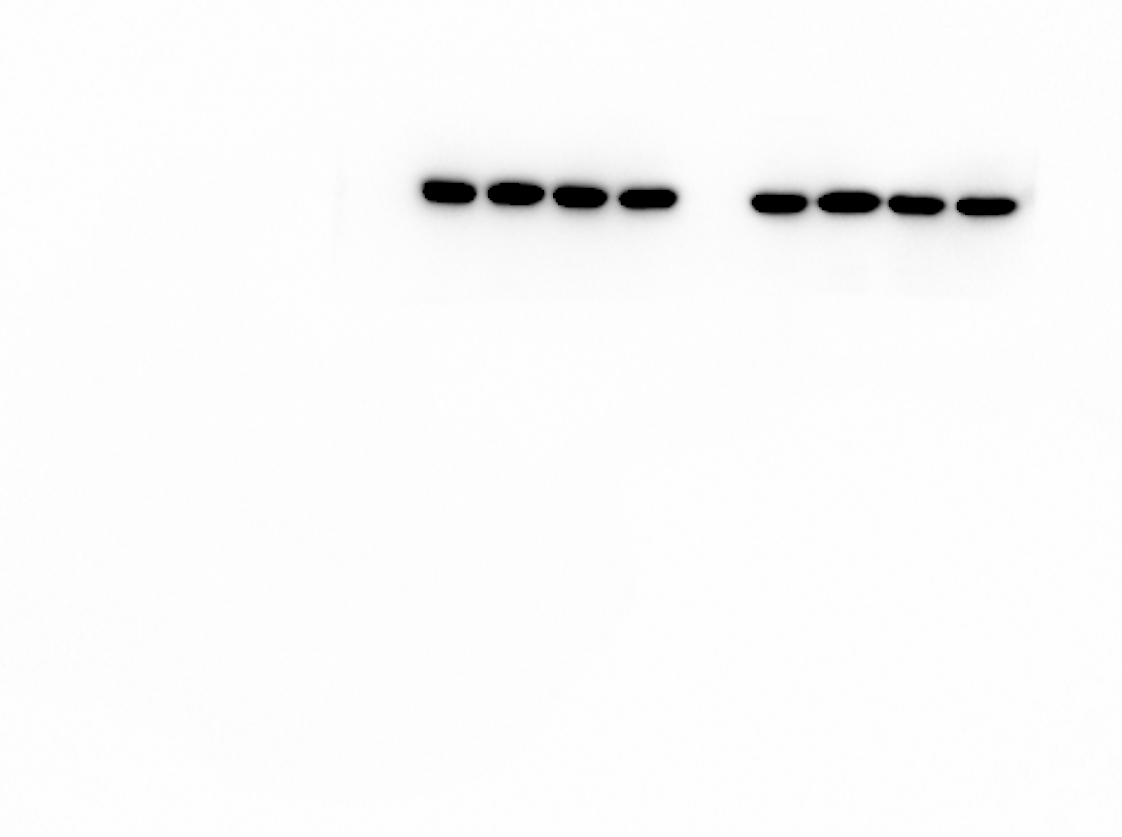

Supplement: Figure 5—figure supplement 1—source data 1. [file elife-82826-fig5-figsupp1-data1.zip › Figure 5-figure supplement 1-source data 1/Unlabeled Western blot/Fig 5-fig sup 1G-GAPDH.tif]

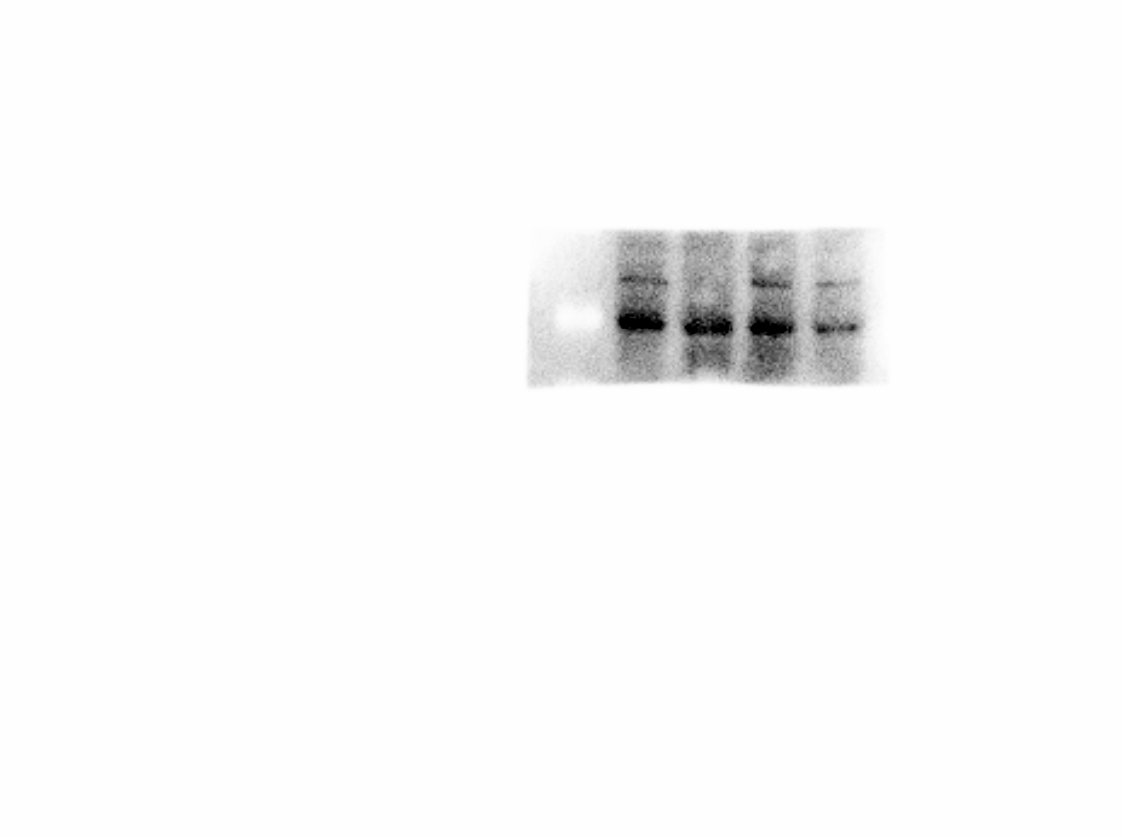

Supplement: Figure 5—figure supplement 1—source data 1. [file elife-82826-fig5-figsupp1-data1.zip › Figure 5-figure supplement 1-source data 1/Unlabeled Western blot/Fig 5-fig sup 1G-TP73.tif]

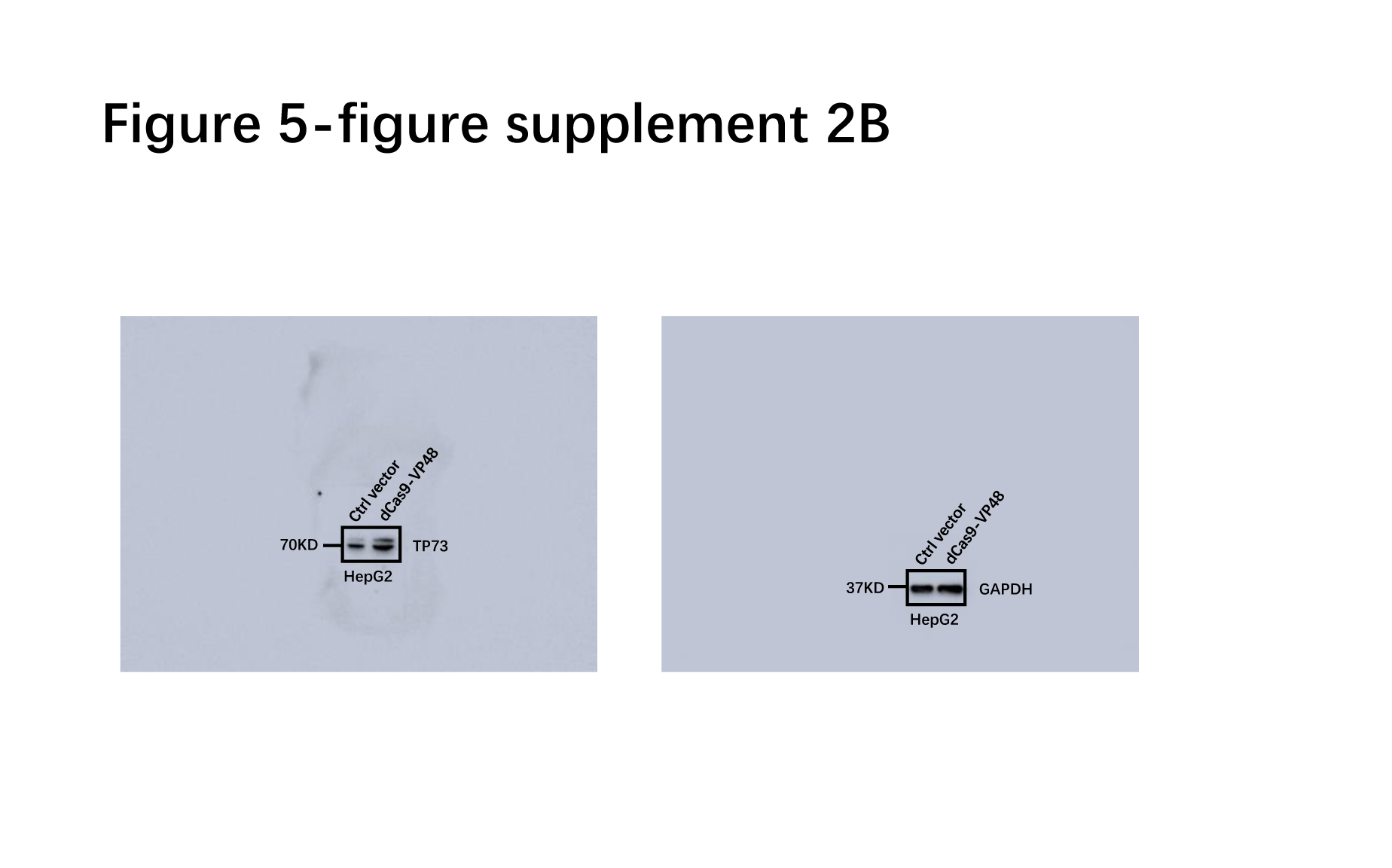

Supplement: Figure 5—figure supplement 2—source data 1. [file elife-82826-fig5-figsupp2-data1.zip › Figure 5-figure supplement 2-source data 1/Labeled Western blot/Figure 5-figure supplement 2B.tif]

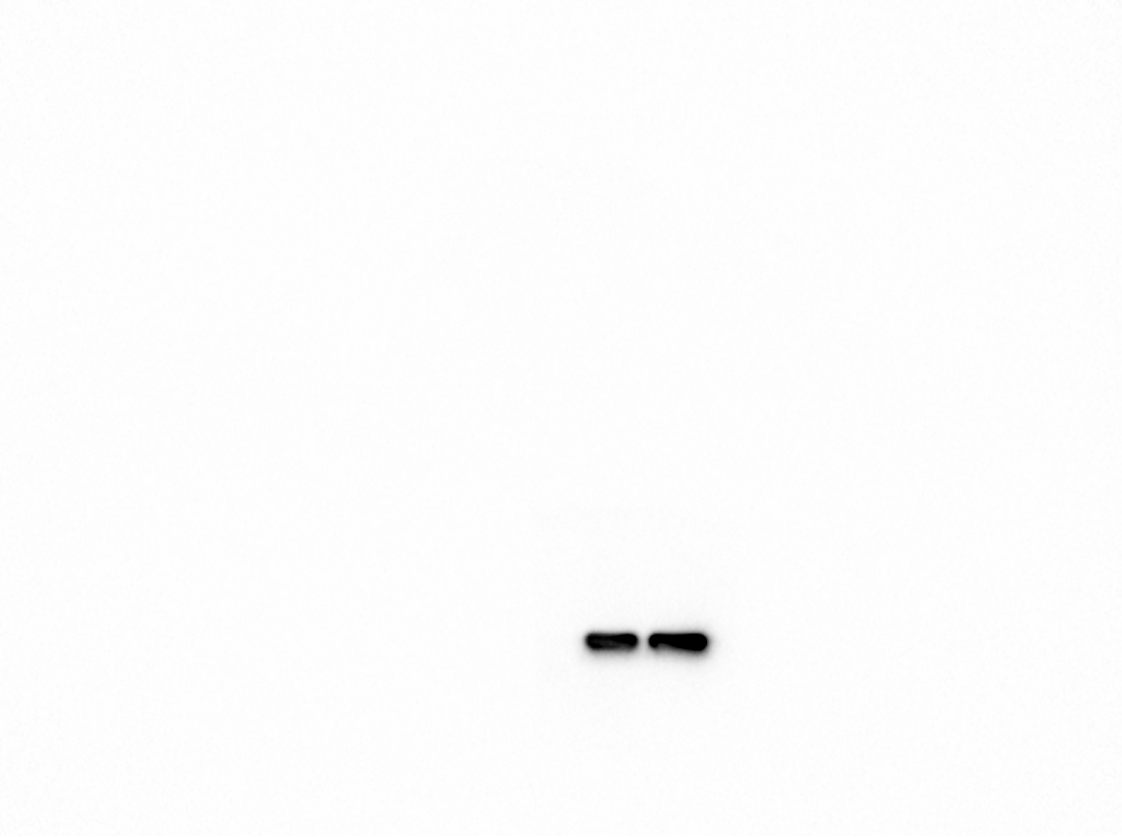

Supplement: Figure 5—figure supplement 2—source data 1. [file elife-82826-fig5-figsupp2-data1.zip › Figure 5-figure supplement 2-source data 1/Unlabeled Western blot/Fig 5- fig sup 2B-GAPDH.tif]

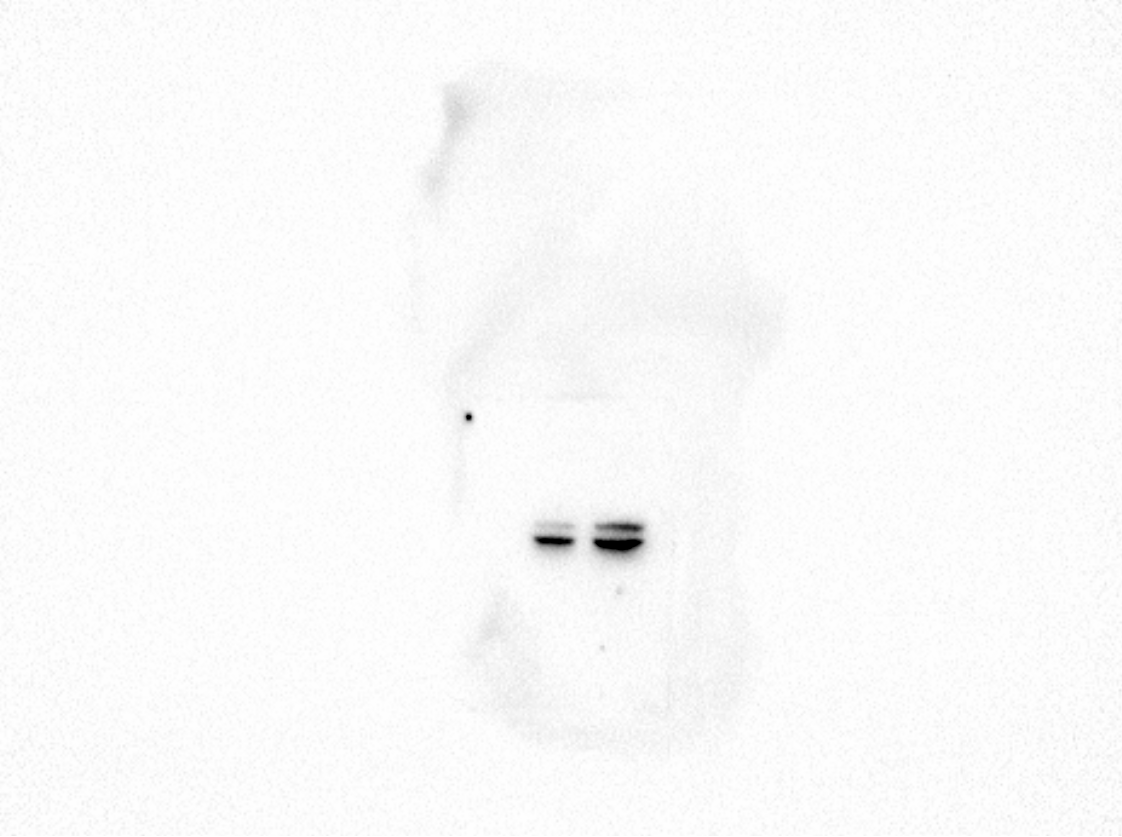

Supplement: Figure 5—figure supplement 2—source data 1. [file elife-82826-fig5-figsupp2-data1.zip › Figure 5-figure supplement 2-source data 1/Unlabeled Western blot/Fig 5- fig sup 2B-TP73.tif]

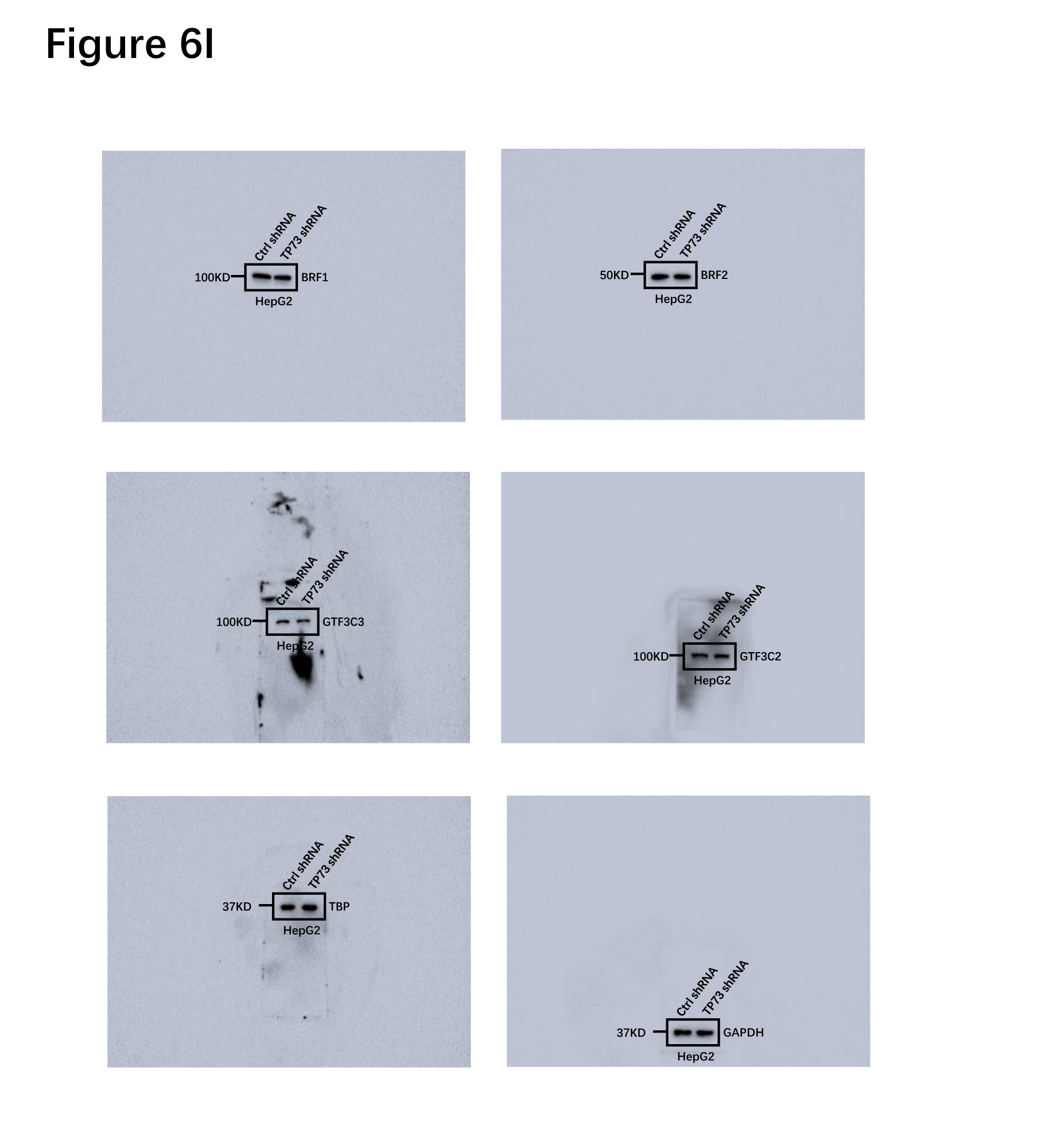

Supplement: Figure 6—source data 1. [file elife-82826-fig6-data1.zip › Figure 6-source data 1/Labeled Western blot/Figure 6I.tif]

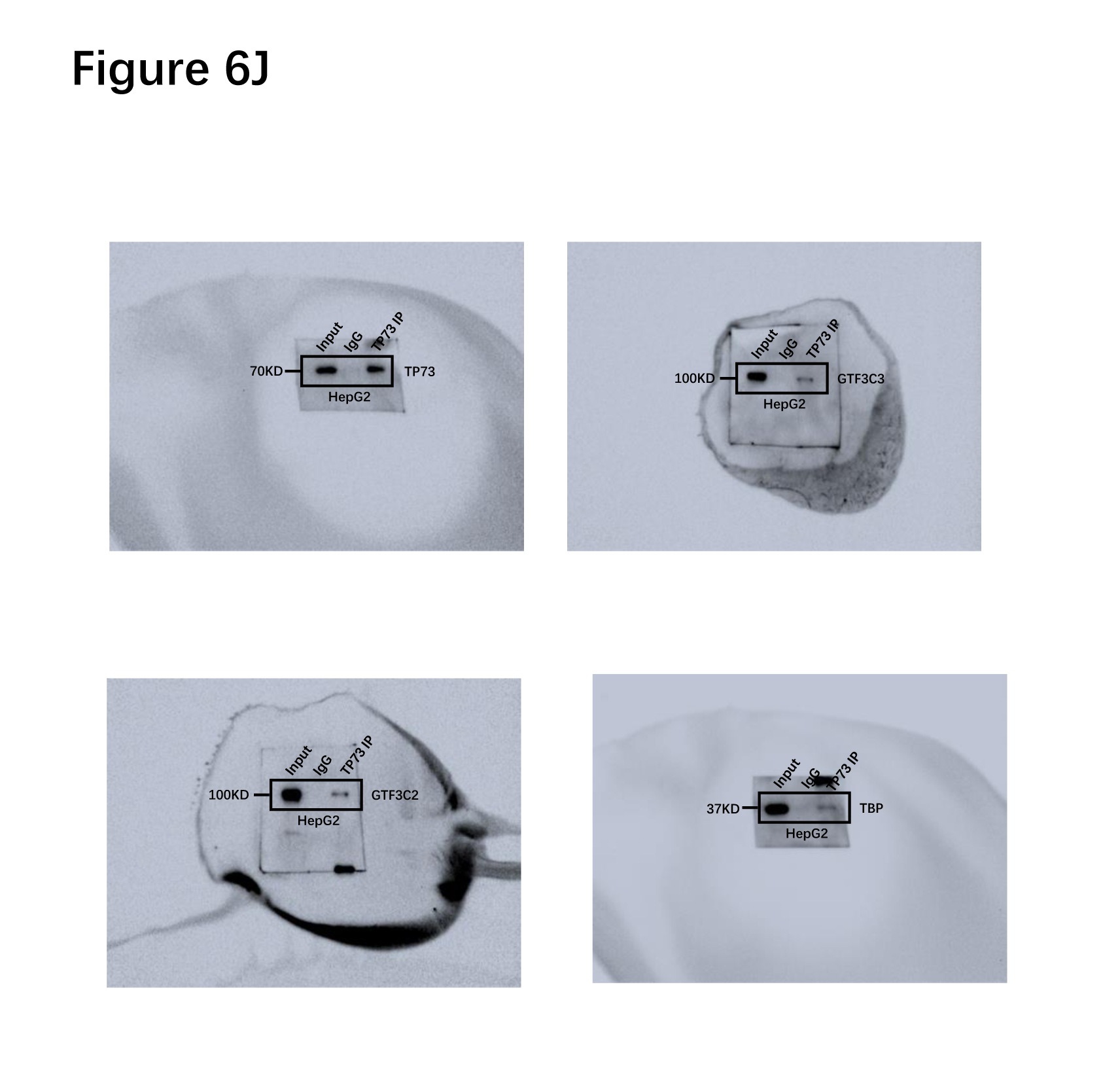

Supplement: Figure 6—source data 1. [file elife-82826-fig6-data1.zip › Figure 6-source data 1/Labeled Western blot/Figure 6J.tif]

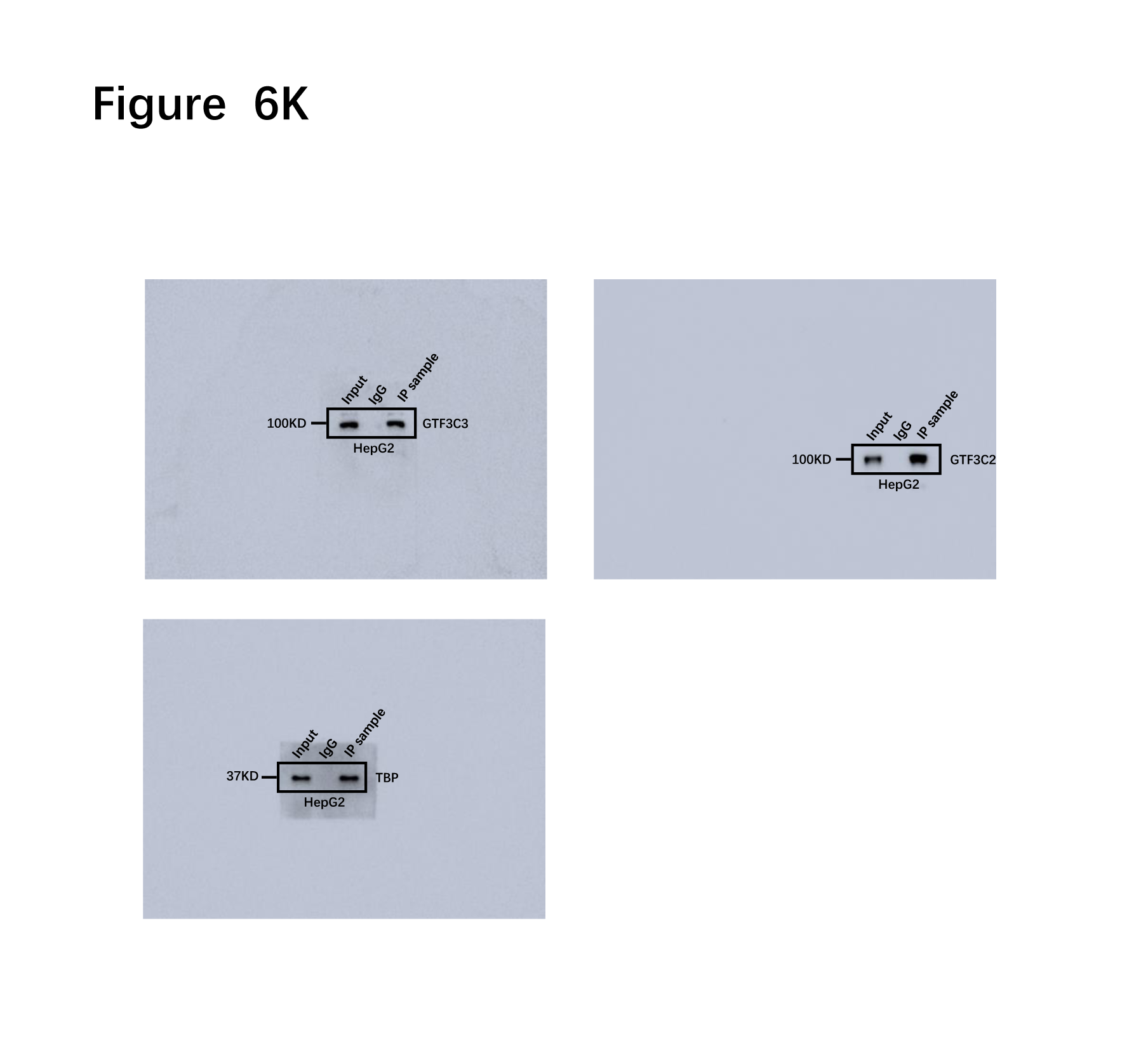

Supplement: Figure 6—source data 1. [file elife-82826-fig6-data1.zip › Figure 6-source data 1/Labeled Western blot/Figure 6K.tif]

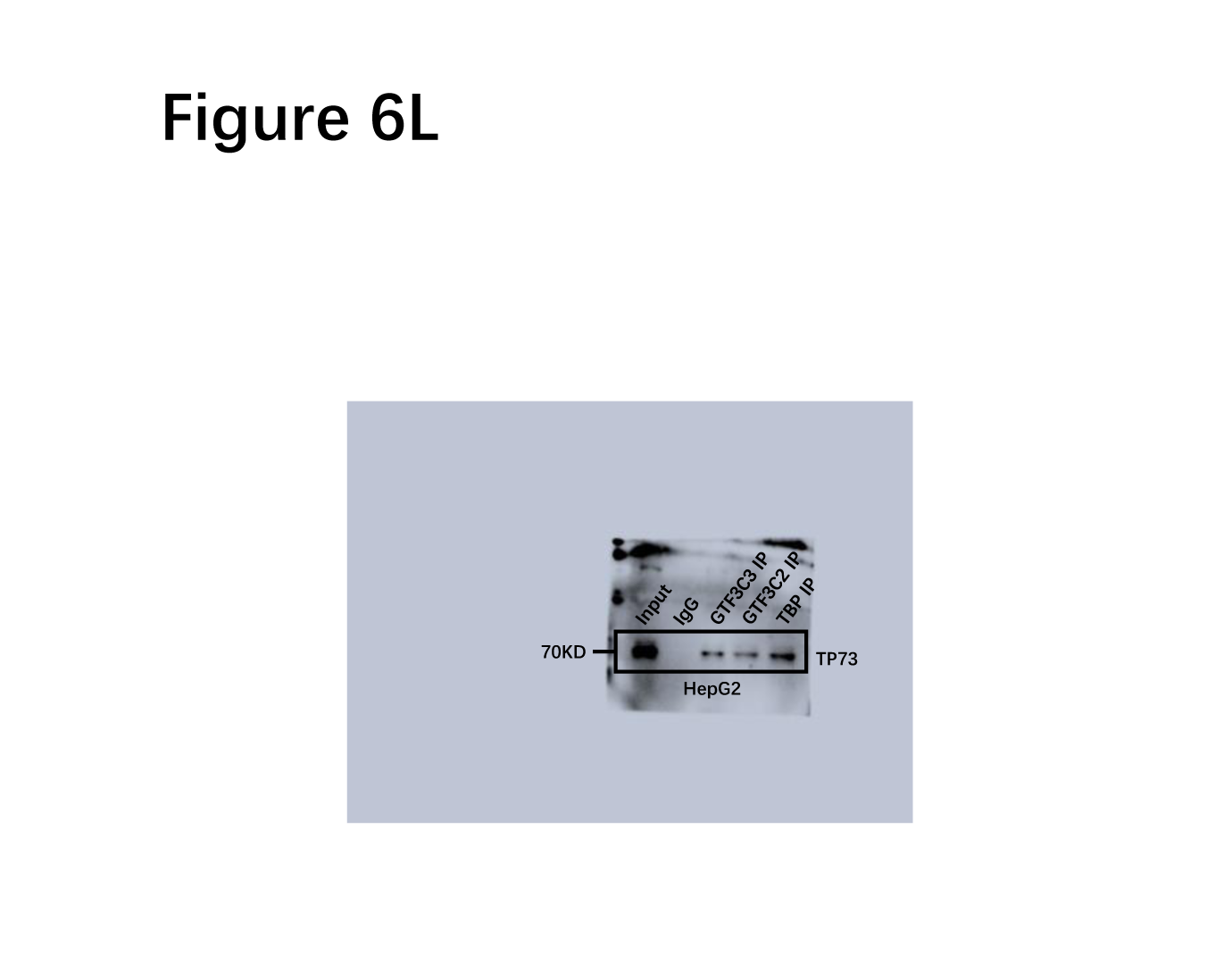

Supplement: Figure 6—source data 1. [file elife-82826-fig6-data1.zip › Figure 6-source data 1/Labeled Western blot/Figure 6L.tif]

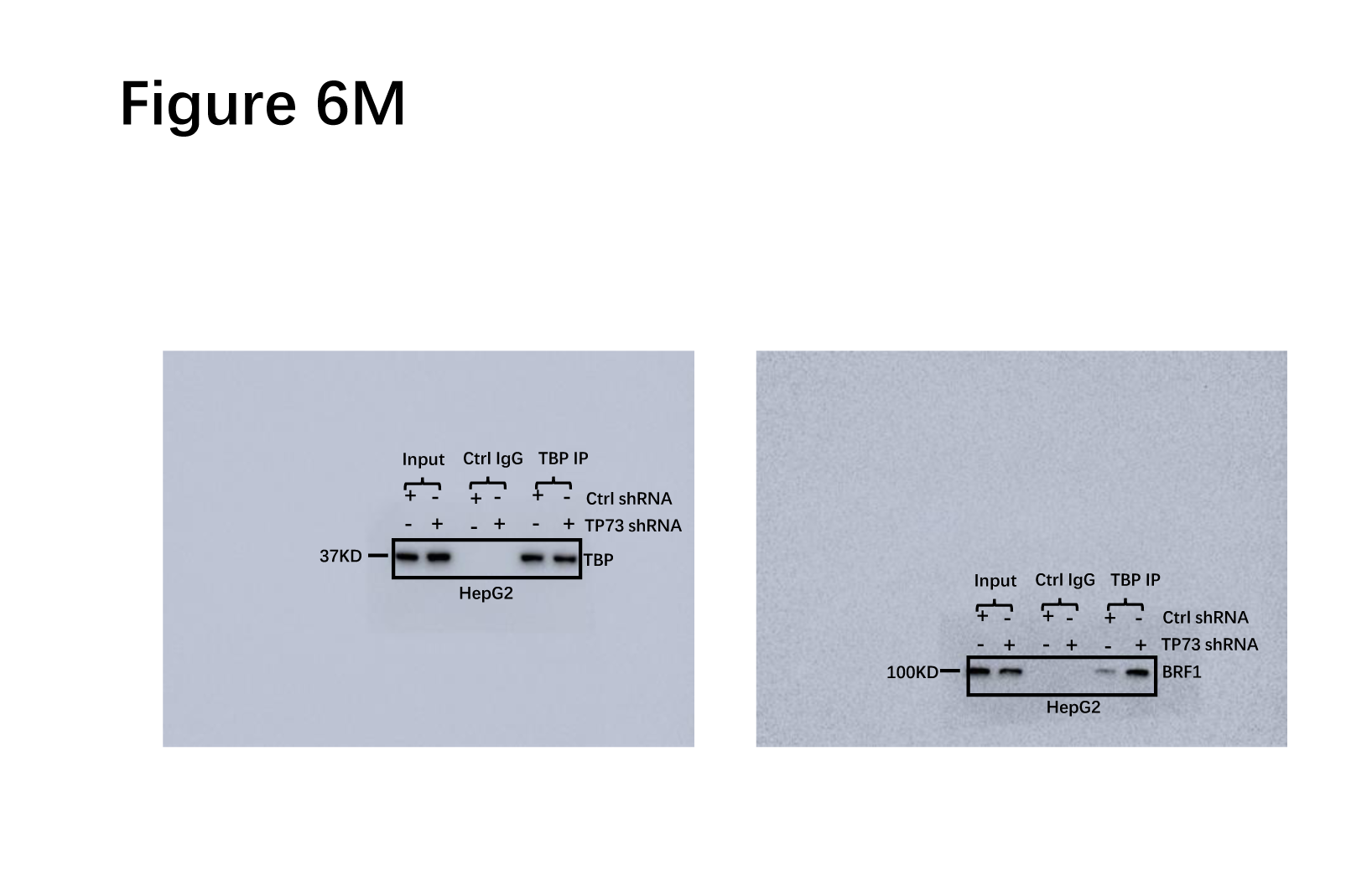

Supplement: Figure 6—source data 1. [file elife-82826-fig6-data1.zip › Figure 6-source data 1/Labeled Western blot/Figure 6M.tif]

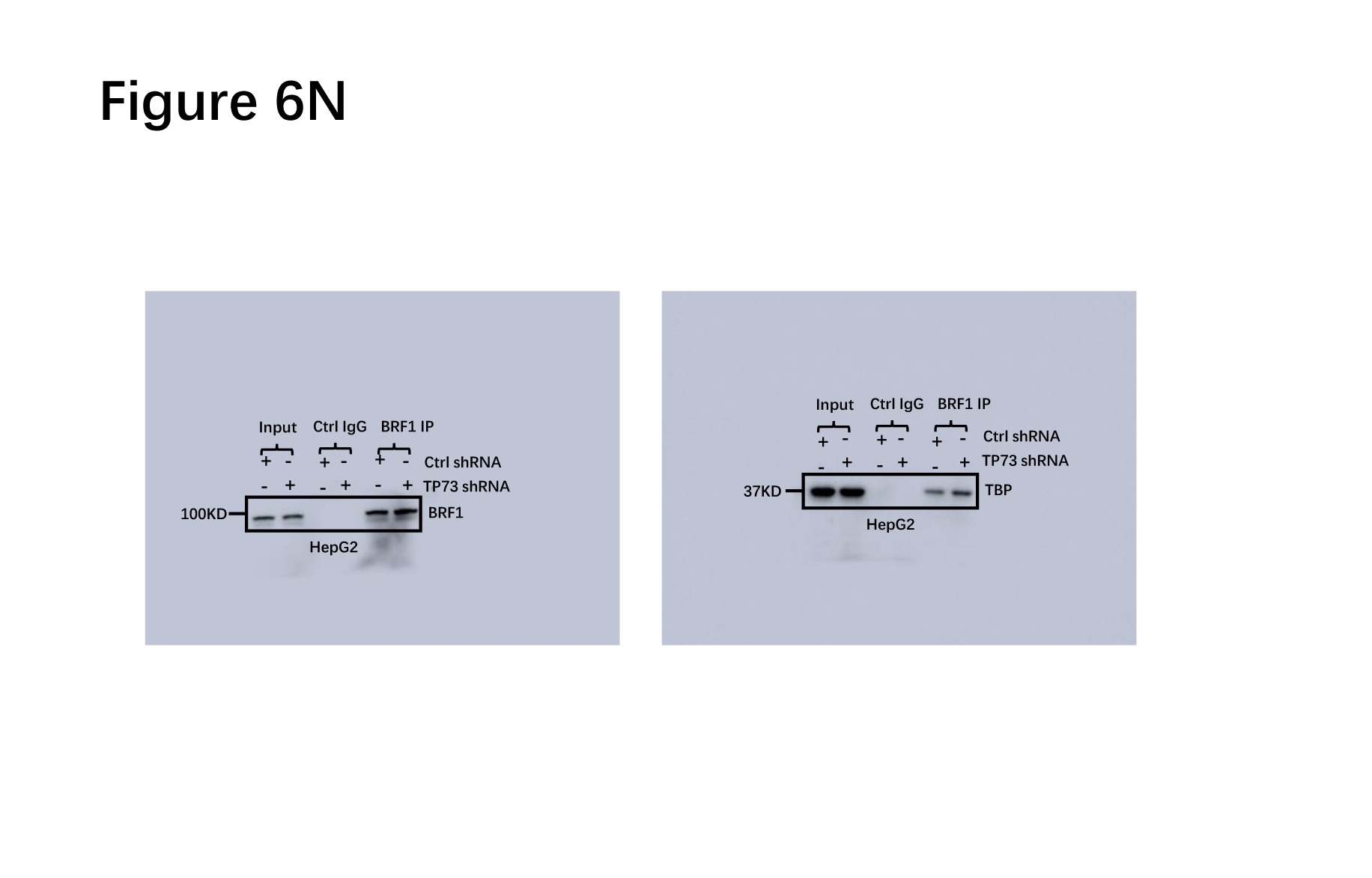

Supplement: Figure 6—source data 1. [file elife-82826-fig6-data1.zip › Figure 6-source data 1/Labeled Western blot/Figure 6N.tif]

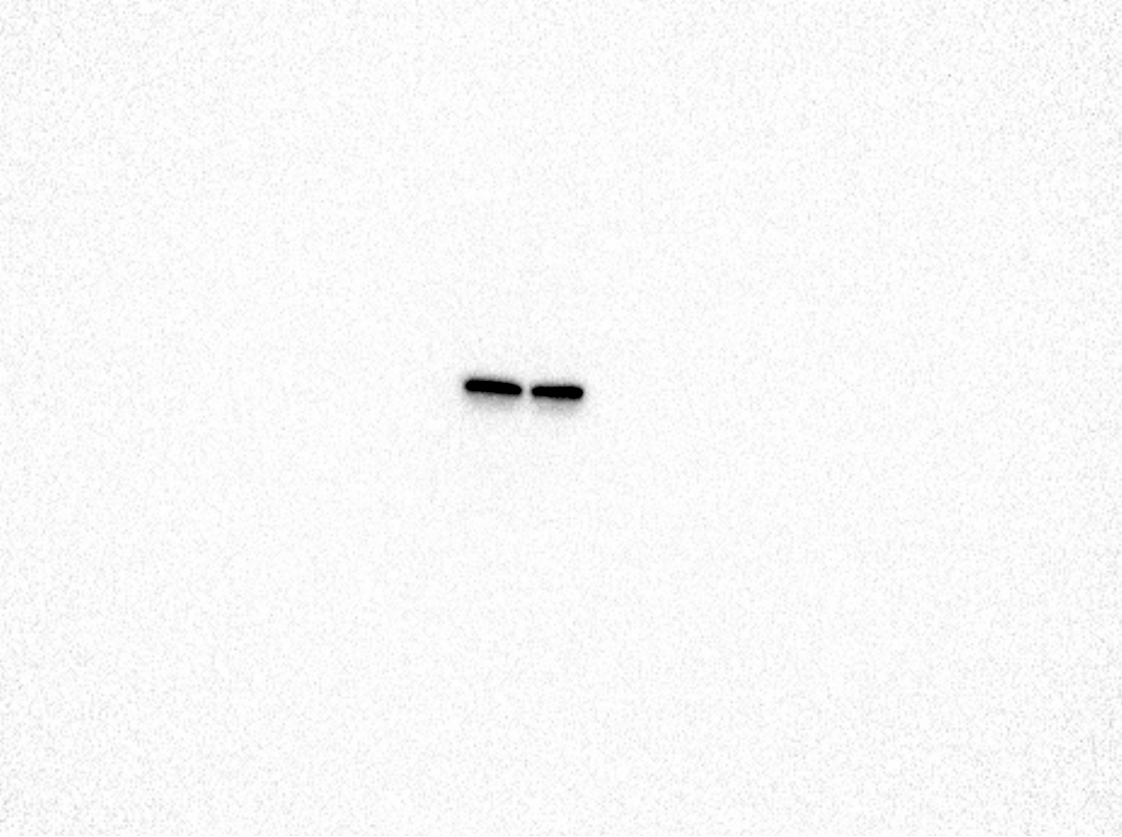

Supplement: Figure 6—source data 1. [file elife-82826-fig6-data1.zip › Figure 6-source data 1/Unlabeled Western blot/Figure 6I-BRF1.tif]

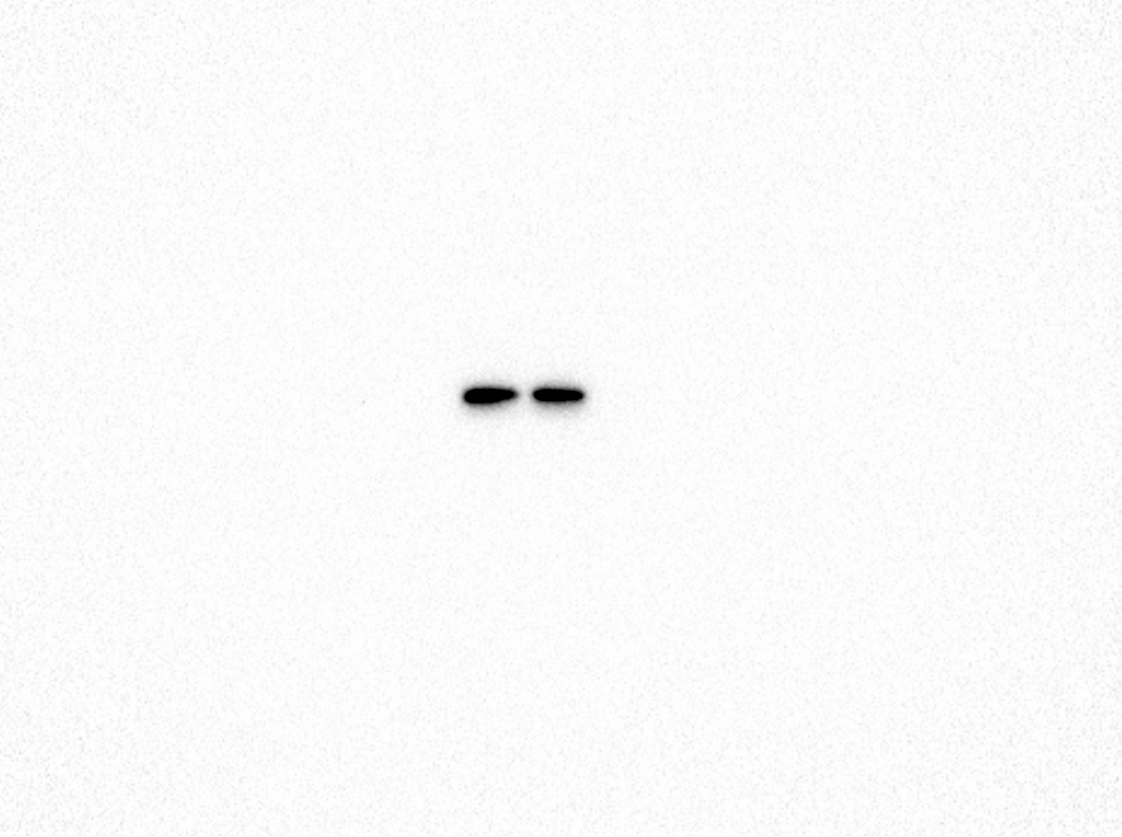

Supplement: Figure 6—source data 1. [file elife-82826-fig6-data1.zip › Figure 6-source data 1/Unlabeled Western blot/Figure 6I-BRF2.tif]

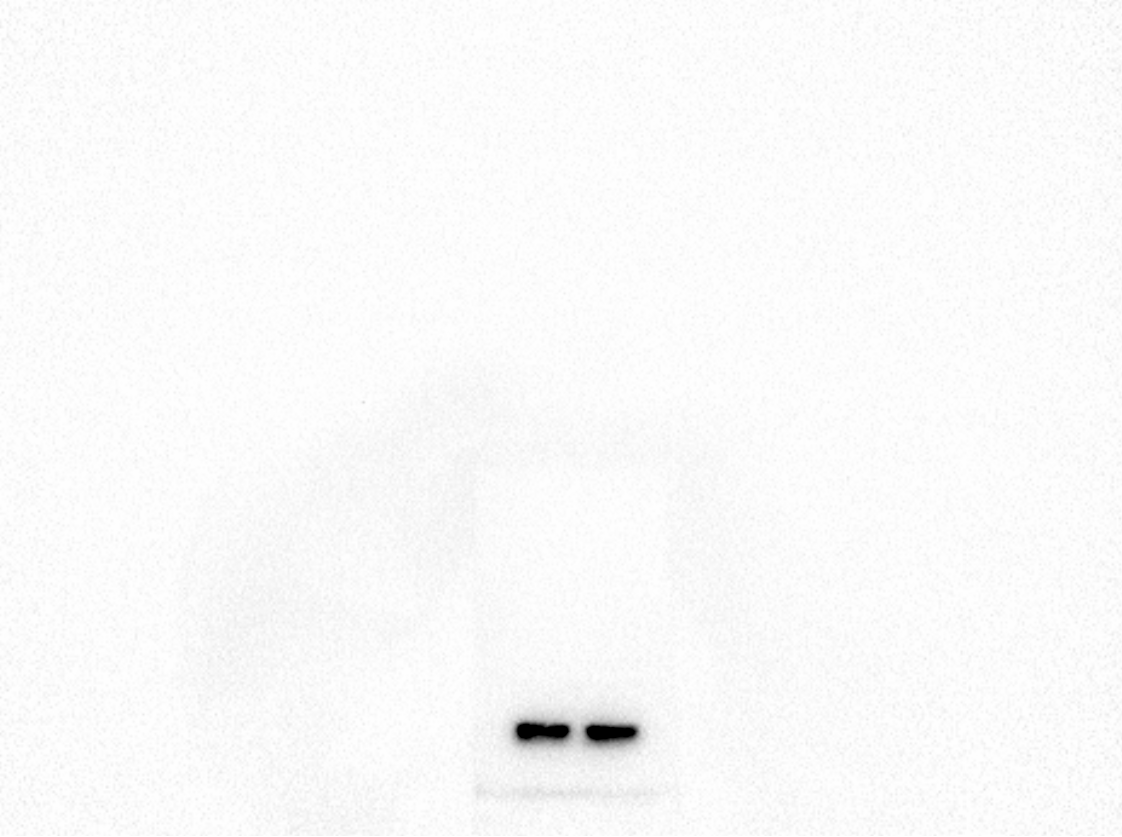

Supplement: Figure 6—source data 1. [file elife-82826-fig6-data1.zip › Figure 6-source data 1/Unlabeled Western blot/Figure 6I-GAPDH.tif]

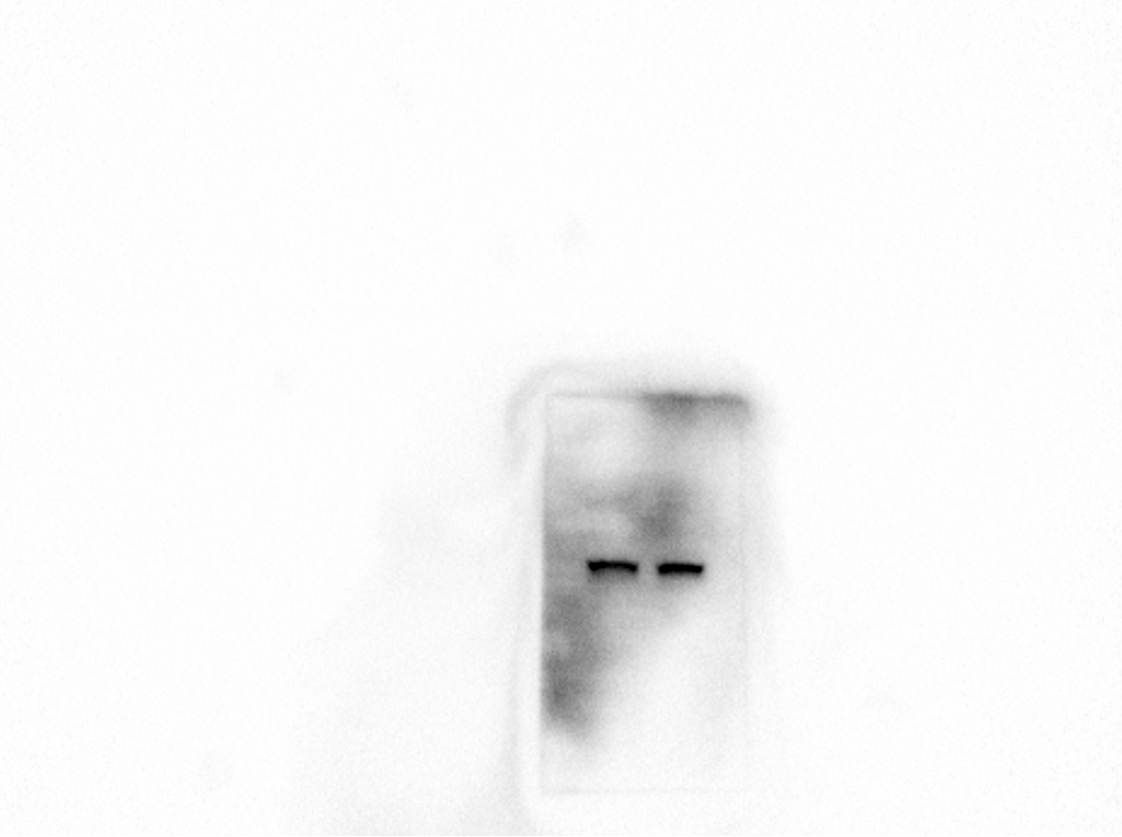

Supplement: Figure 6—source data 1. [file elife-82826-fig6-data1.zip › Figure 6-source data 1/Unlabeled Western blot/Figure 6I-GTF3C2.tif]

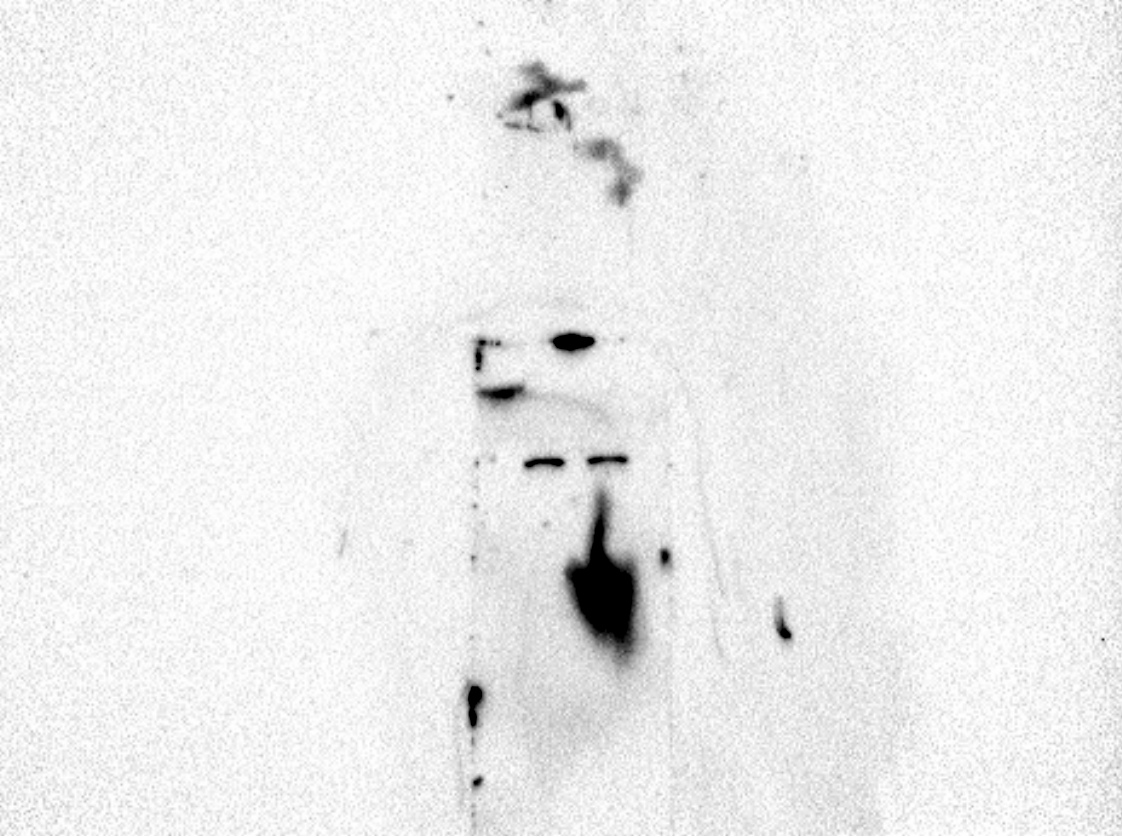

Supplement: Figure 6—source data 1. [file elife-82826-fig6-data1.zip › Figure 6-source data 1/Unlabeled Western blot/Figure 6I-GTF3C3.tif]

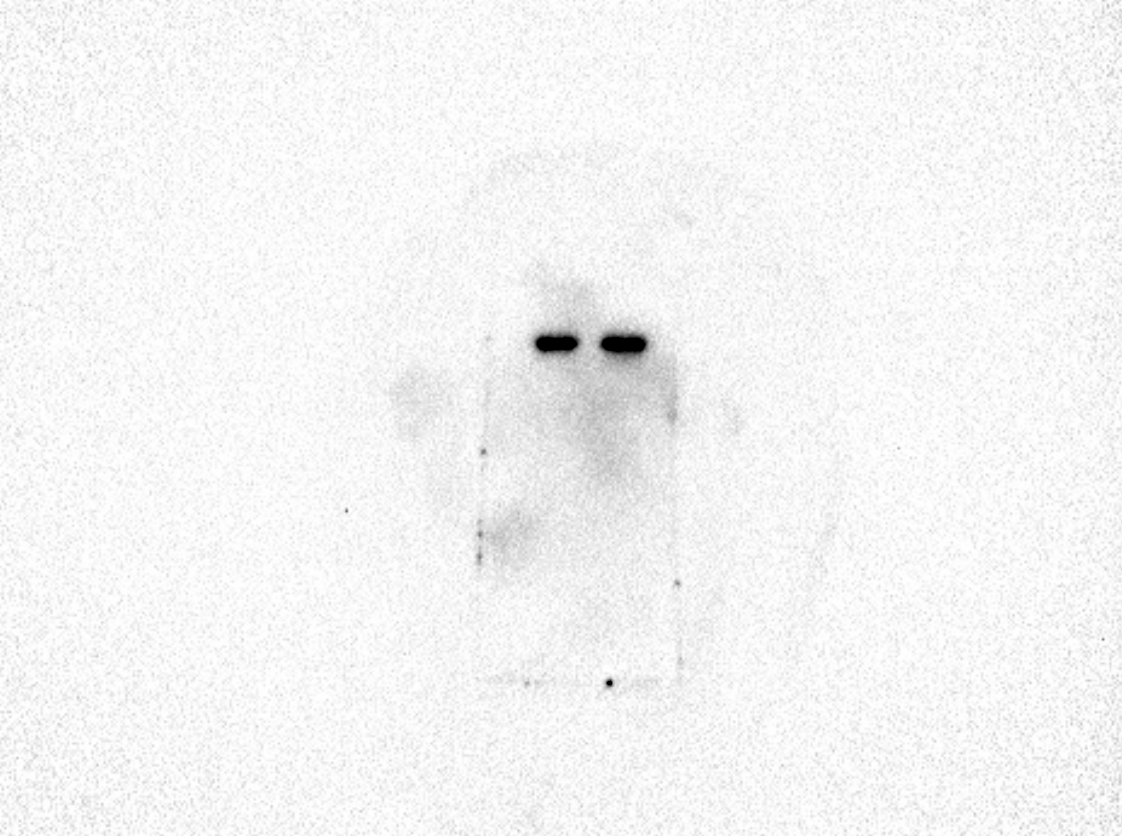

Supplement: Figure 6—source data 1. [file elife-82826-fig6-data1.zip › Figure 6-source data 1/Unlabeled Western blot/Figure 6I-TBP.tif]

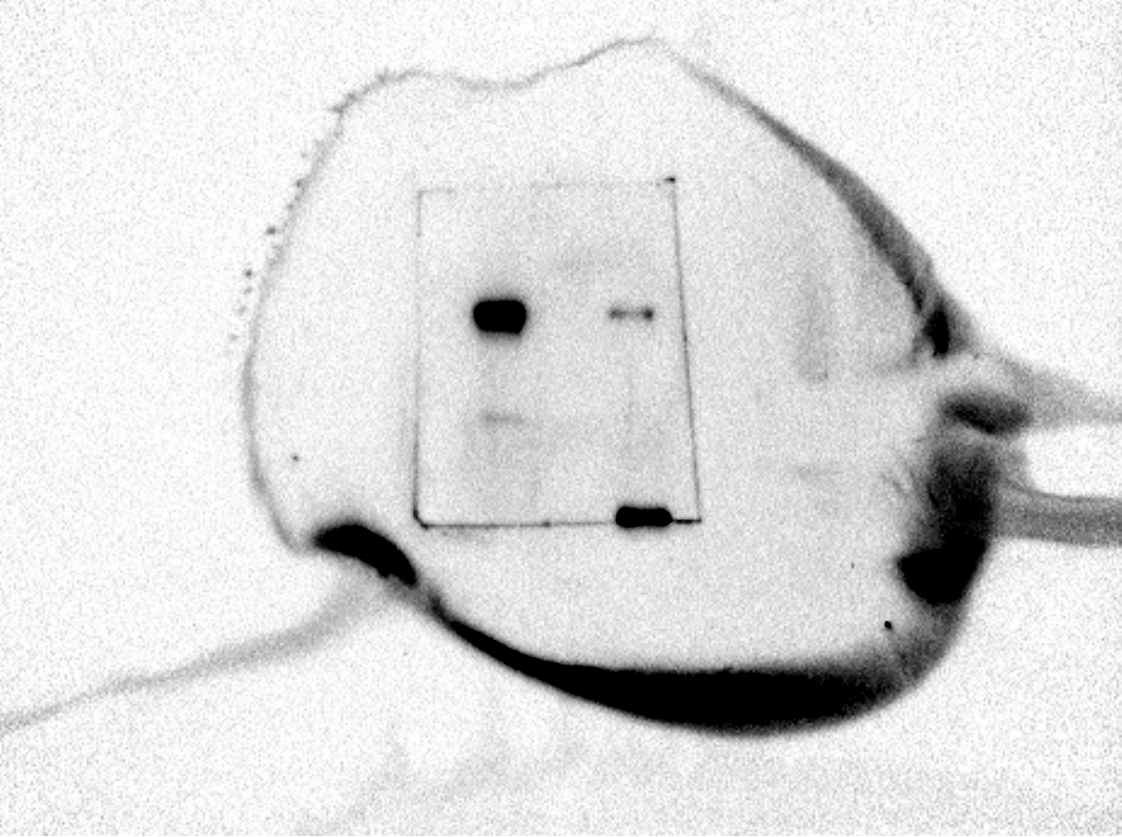

Supplement: Figure 6—source data 1. [file elife-82826-fig6-data1.zip › Figure 6-source data 1/Unlabeled Western blot/Figure 6J-GTF3C2.tif]

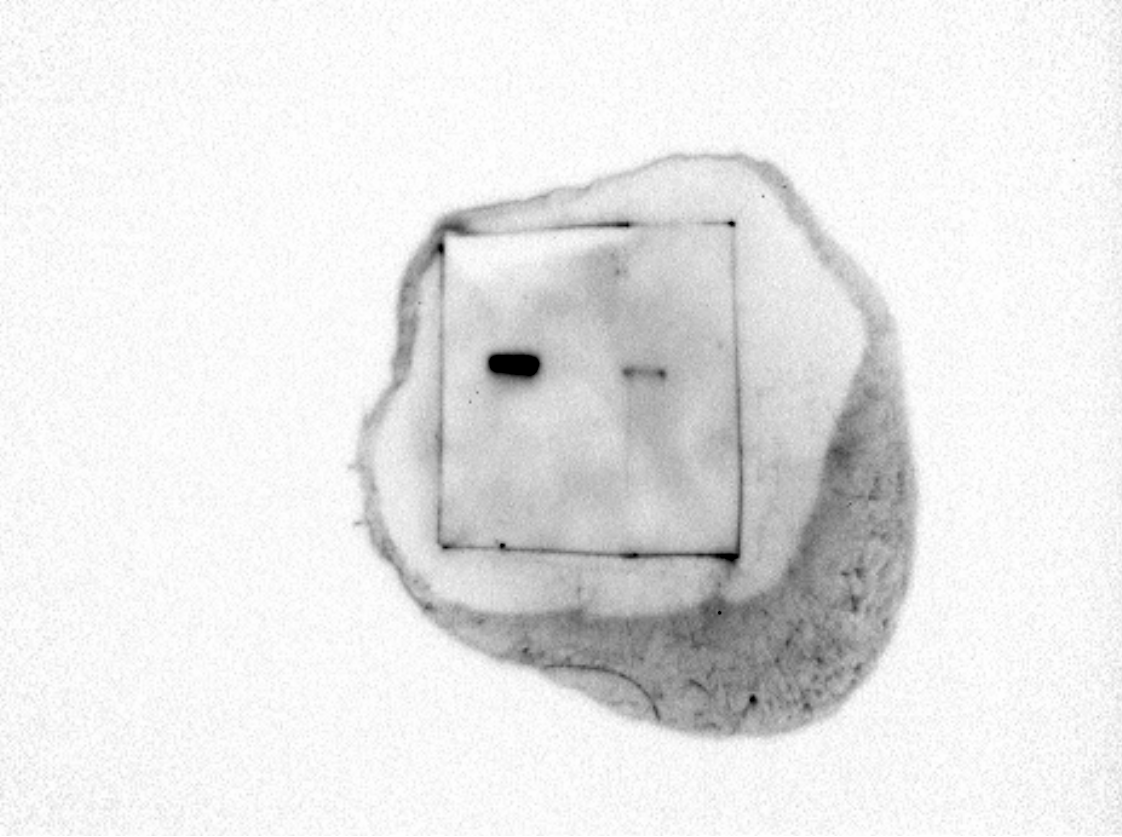

Supplement: Figure 6—source data 1. [file elife-82826-fig6-data1.zip › Figure 6-source data 1/Unlabeled Western blot/Figure 6J-GTF3C3.tif]

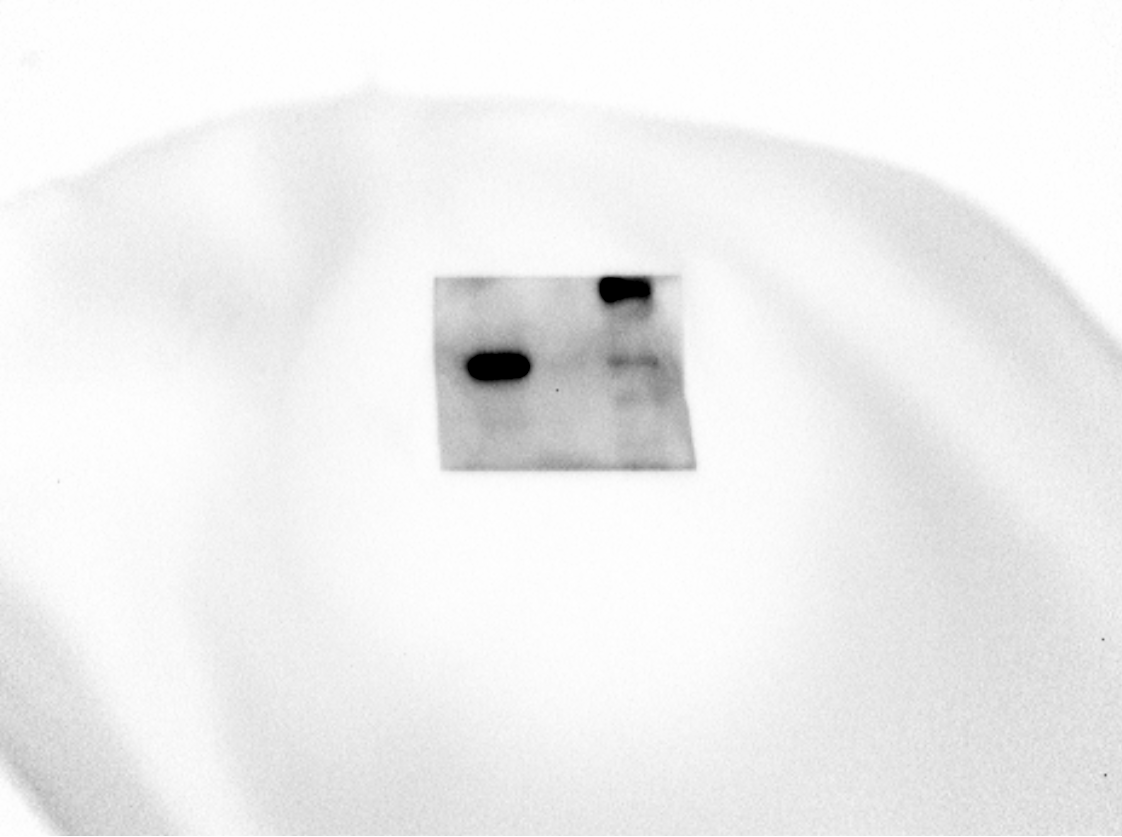

Supplement: Figure 6—source data 1. [file elife-82826-fig6-data1.zip › Figure 6-source data 1/Unlabeled Western blot/Figure 6J-TBP.tif]

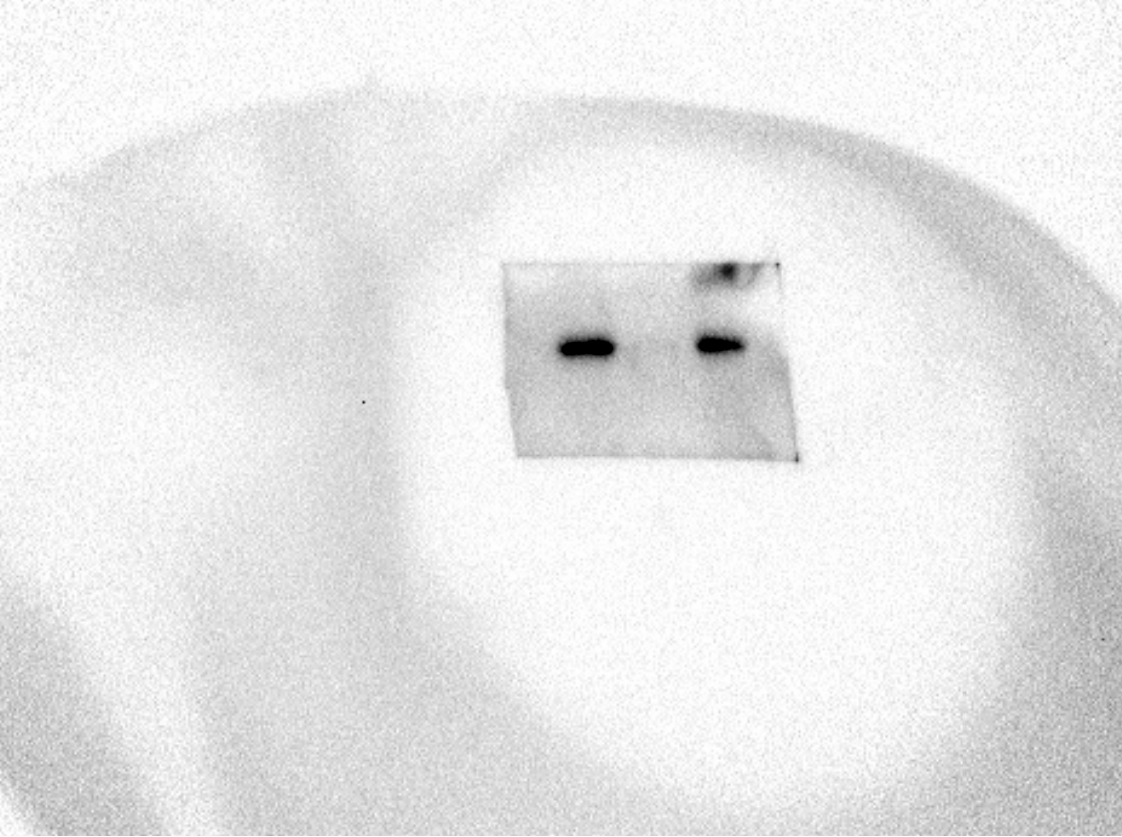

Supplement: Figure 6—source data 1. [file elife-82826-fig6-data1.zip › Figure 6-source data 1/Unlabeled Western blot/Figure 6J-TP73.tif]

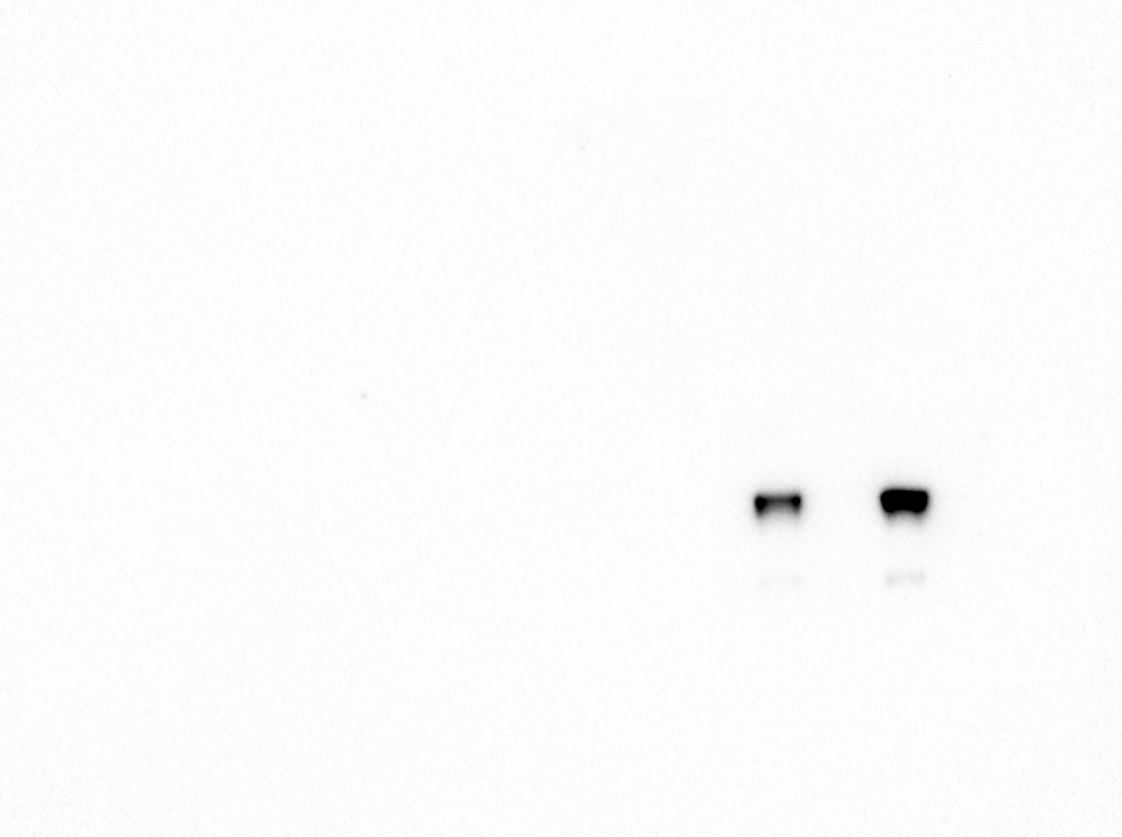

Supplement: Figure 6—source data 1. [file elife-82826-fig6-data1.zip › Figure 6-source data 1/Unlabeled Western blot/Figure 6K-GTF3C2.tif]

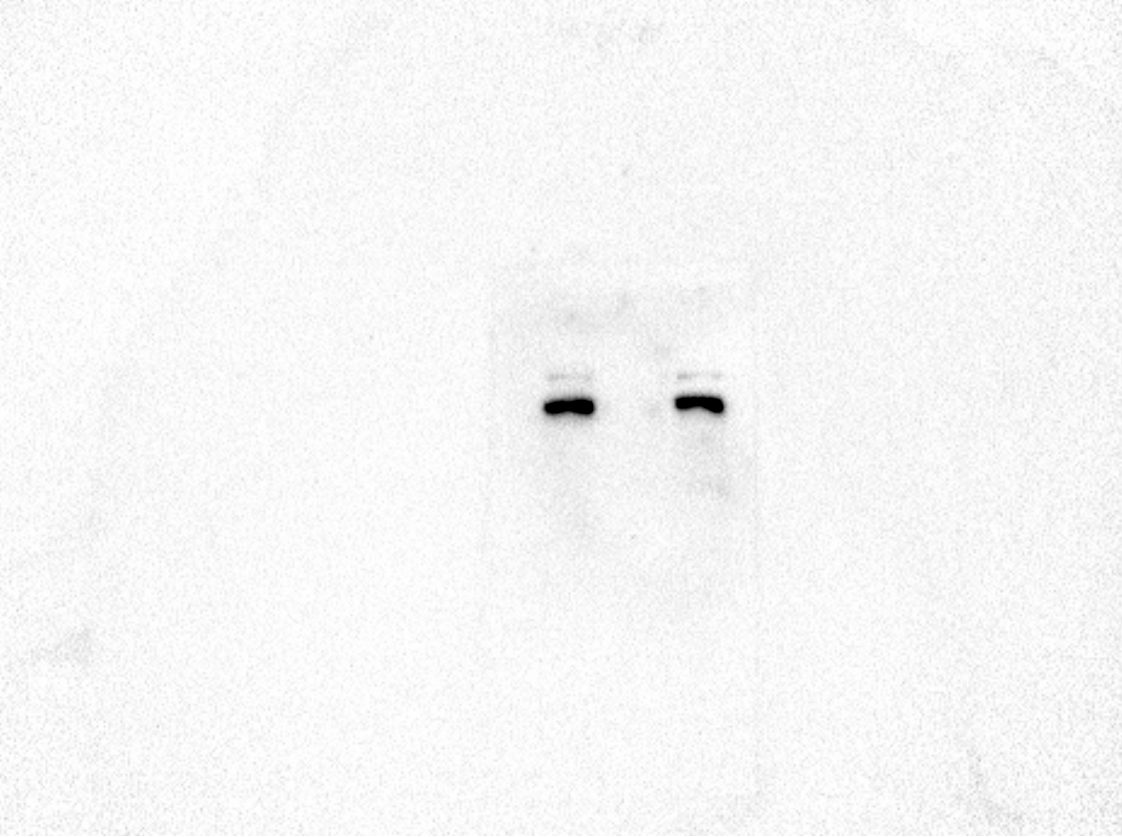

Supplement: Figure 6—source data 1. [file elife-82826-fig6-data1.zip › Figure 6-source data 1/Unlabeled Western blot/Figure 6K-GTF3C3.tif]

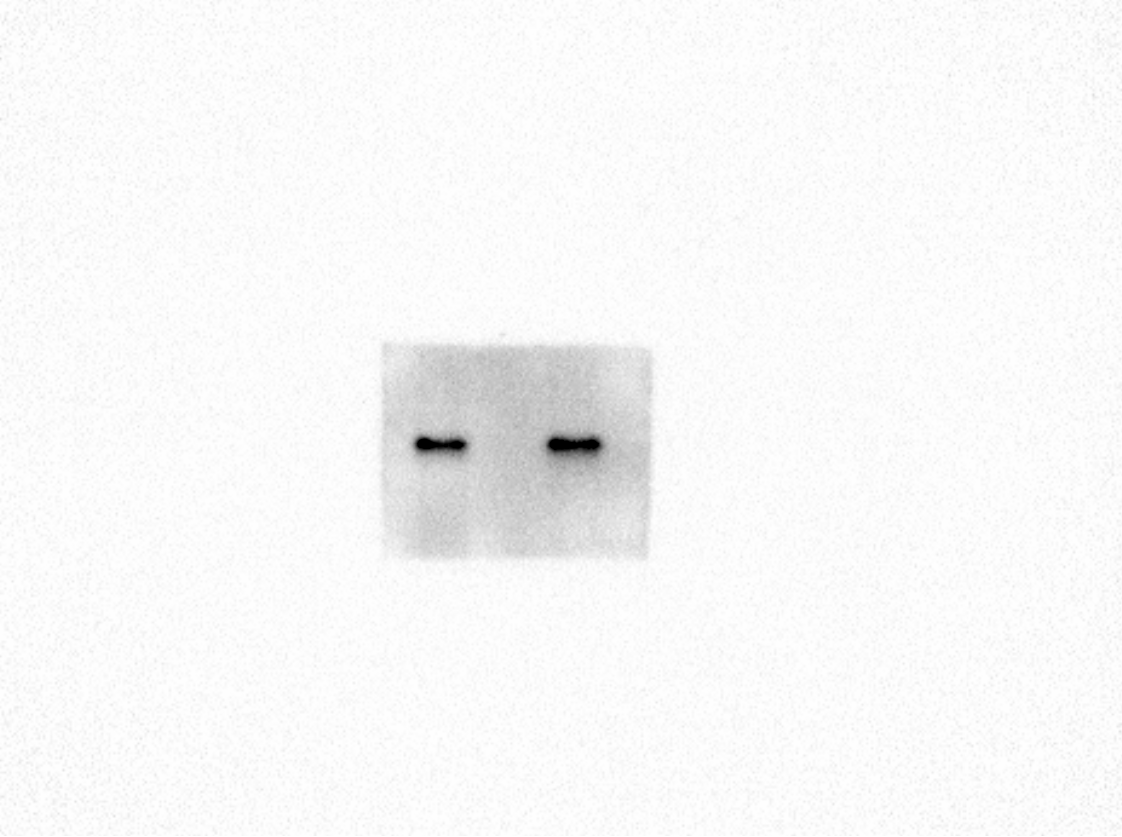

Supplement: Figure 6—source data 1. [file elife-82826-fig6-data1.zip › Figure 6-source data 1/Unlabeled Western blot/Figure 6K-TBP.tif]

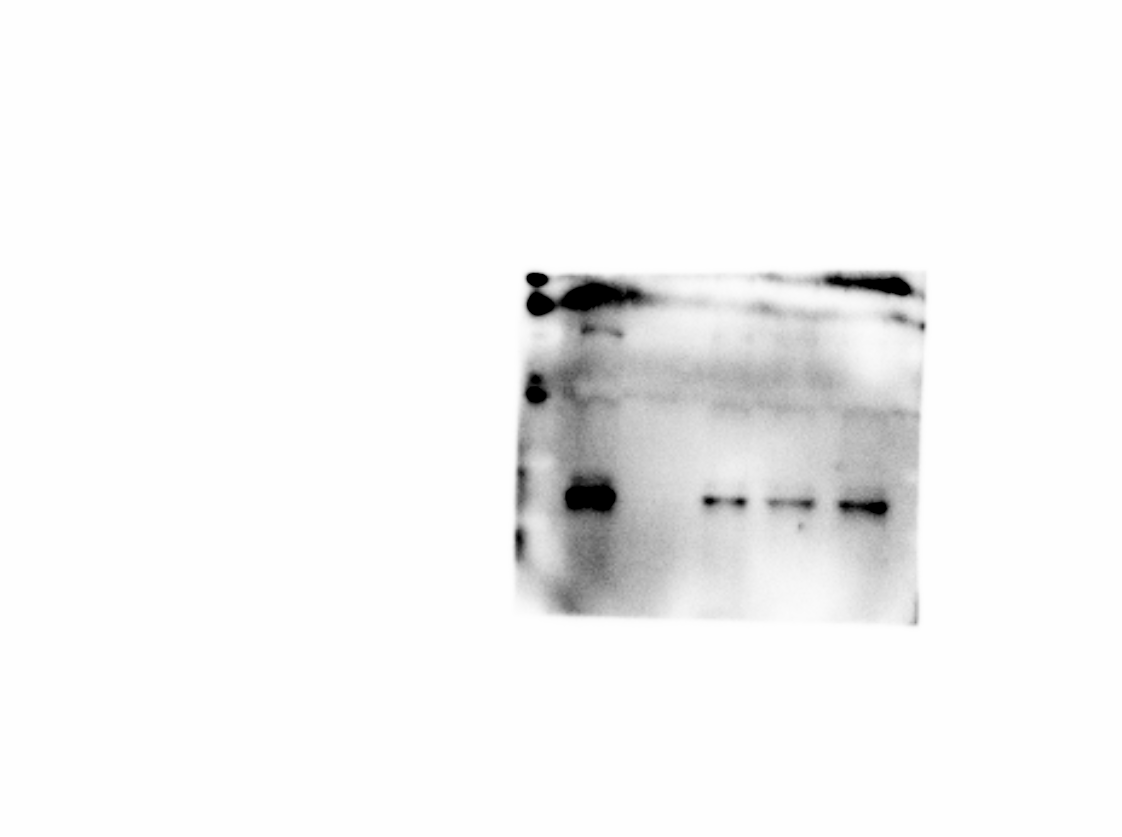

Supplement: Figure 6—source data 1. [file elife-82826-fig6-data1.zip › Figure 6-source data 1/Unlabeled Western blot/Figure 6L-TP73.tif]

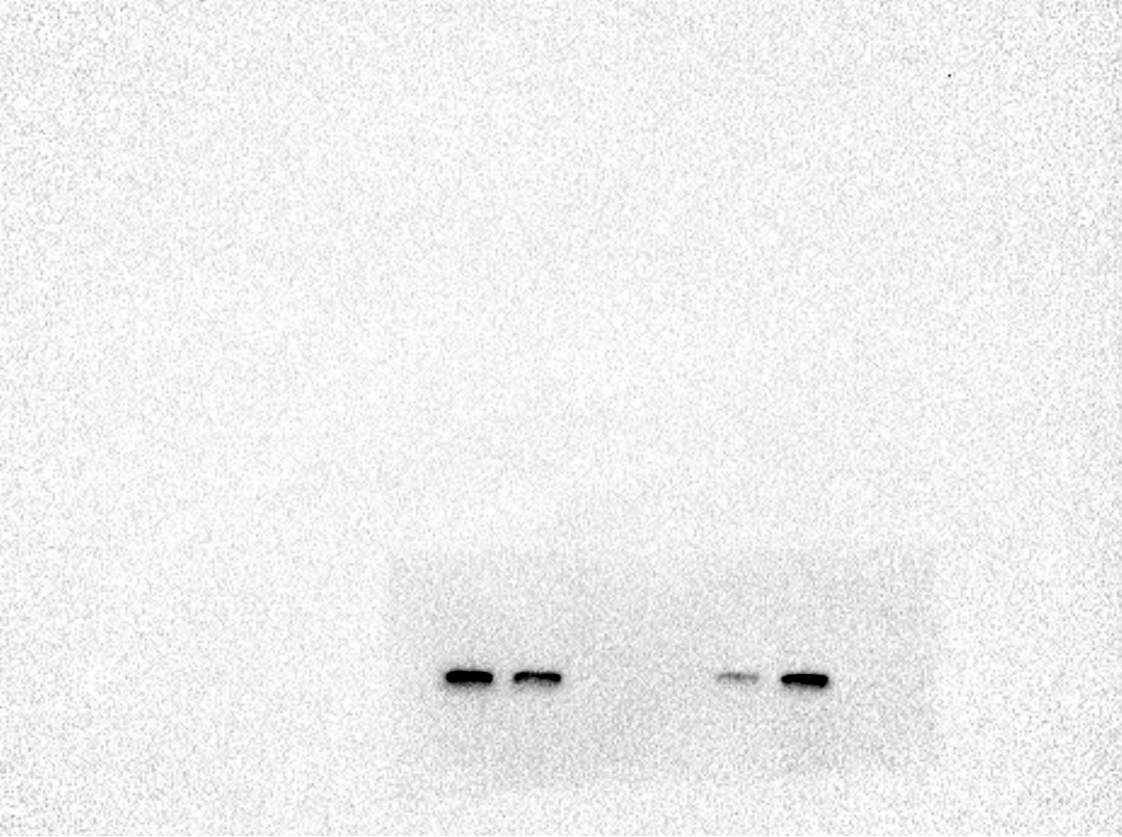

Supplement: Figure 6—source data 1. [file elife-82826-fig6-data1.zip › Figure 6-source data 1/Unlabeled Western blot/Figure 6M-BRF1.tif]

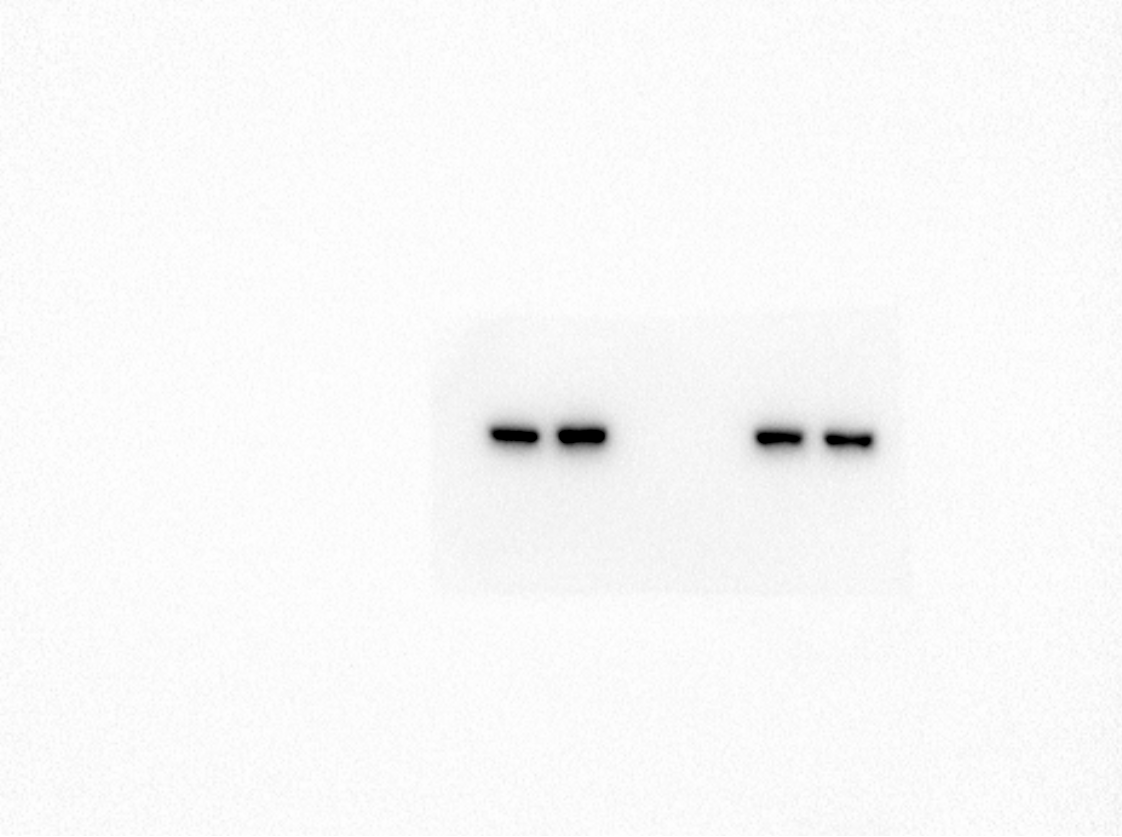

Supplement: Figure 6—source data 1. [file elife-82826-fig6-data1.zip › Figure 6-source data 1/Unlabeled Western blot/Figure 6M-TBP.tif]

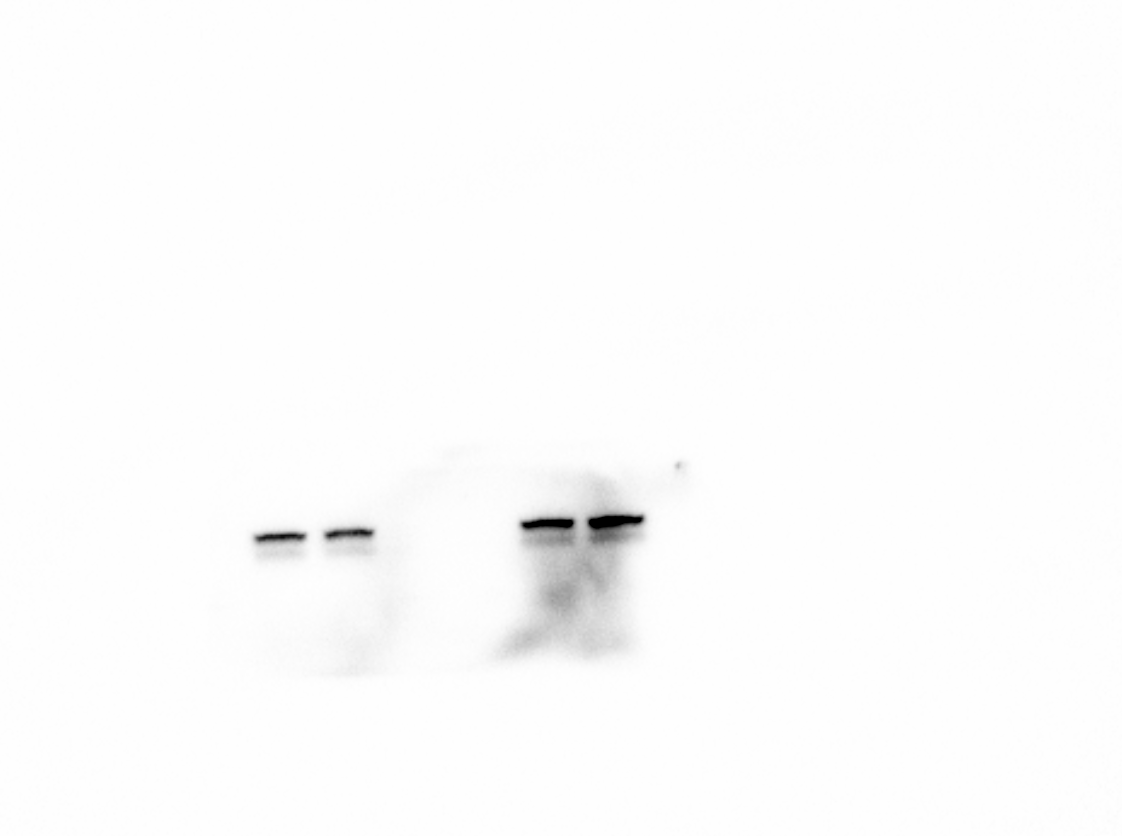

Supplement: Figure 6—source data 1. [file elife-82826-fig6-data1.zip › Figure 6-source data 1/Unlabeled Western blot/Figure 6N-BRF1.tif]

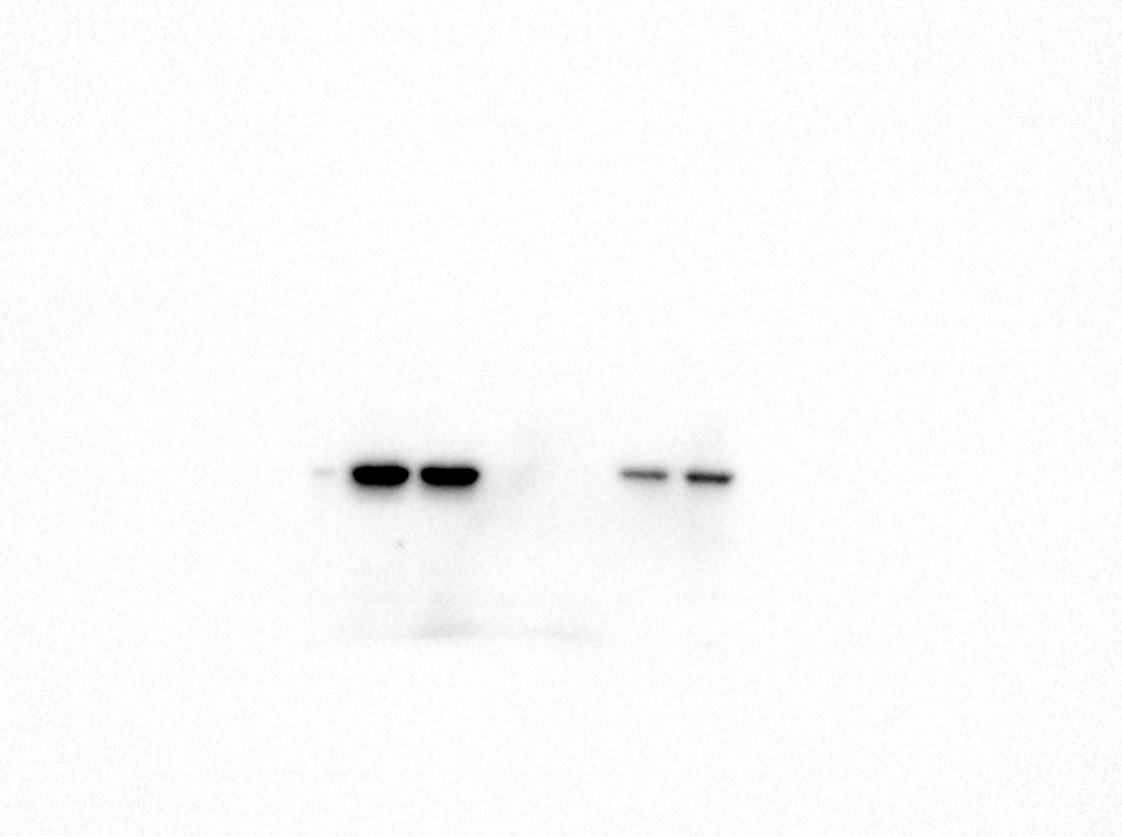

Supplement: Figure 6—source data 1. [file elife-82826-fig6-data1.zip › Figure 6-source data 1/Unlabeled Western blot/Figure 6N-TBP.tif]

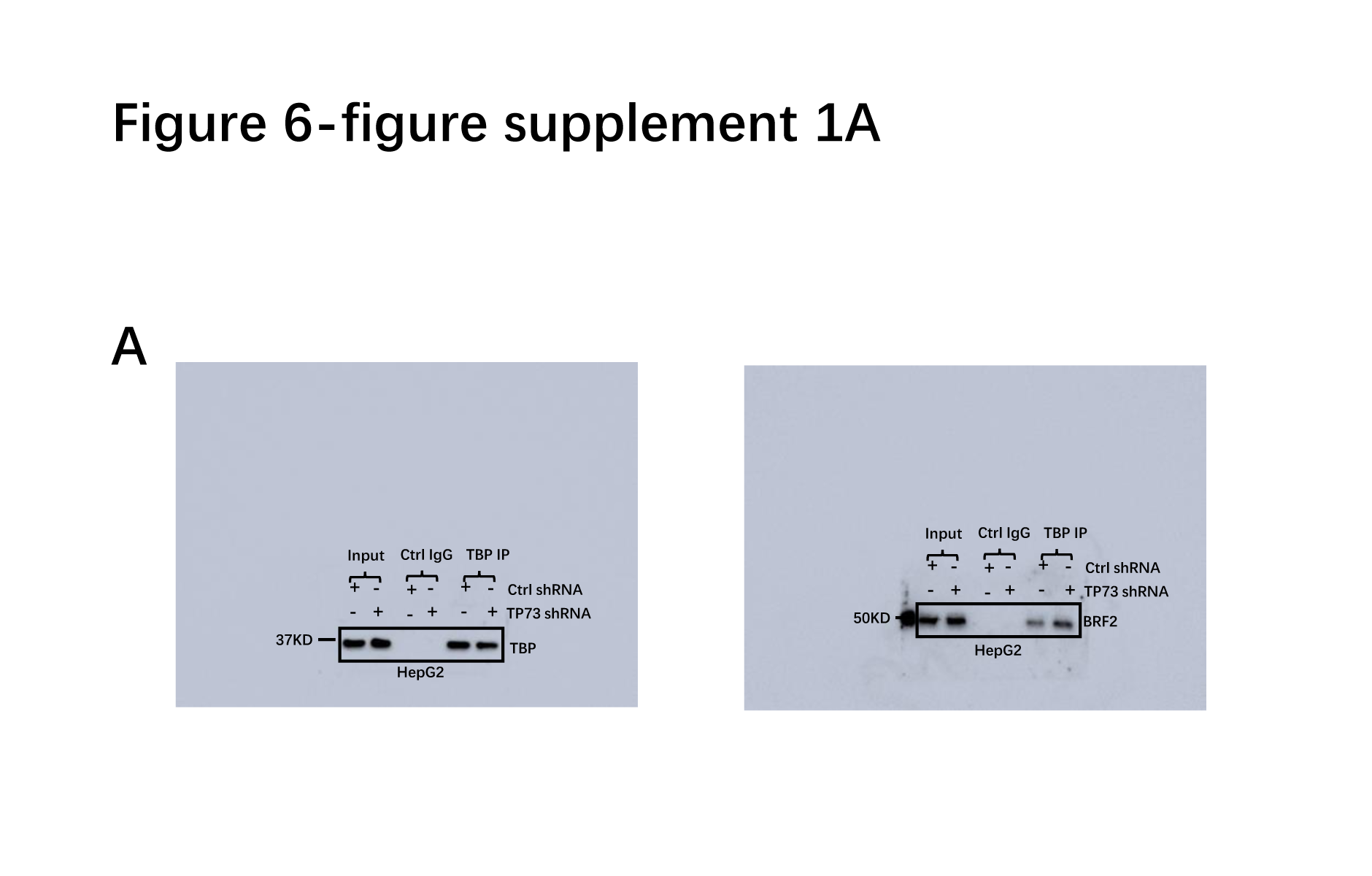

Supplement: Figure 6—figure supplement 1—source data 1. [file elife-82826-fig6-figsupp1-data1.zip › Figure 6-figure supplement 1-source data 1/Labeled Western blot/Figure 6-figure supplement 1A.tif]

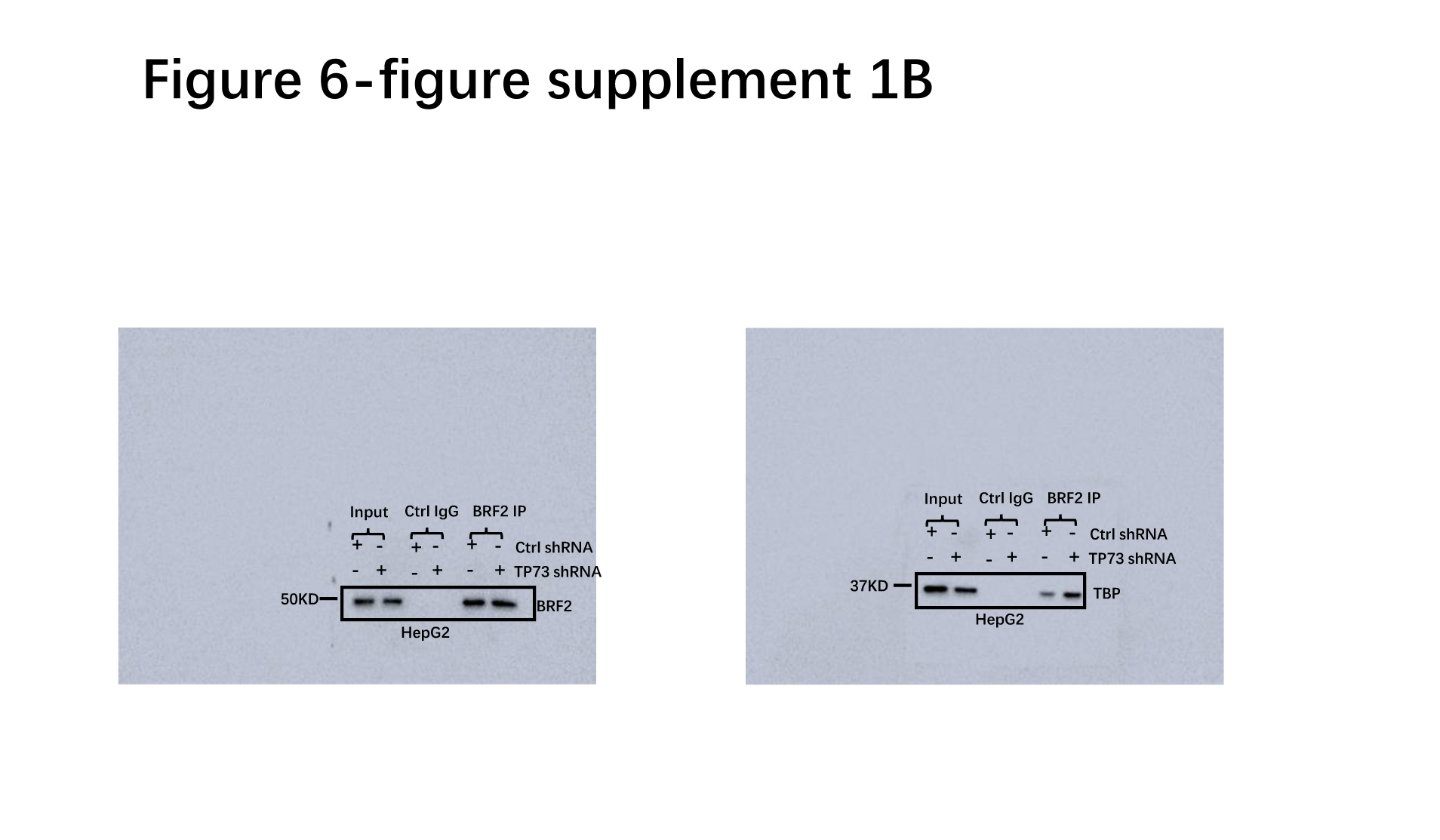

Supplement: Figure 6—figure supplement 1—source data 1. [file elife-82826-fig6-figsupp1-data1.zip › Figure 6-figure supplement 1-source data 1/Labeled Western blot/Figure 6-figure supplement 1B.tif]

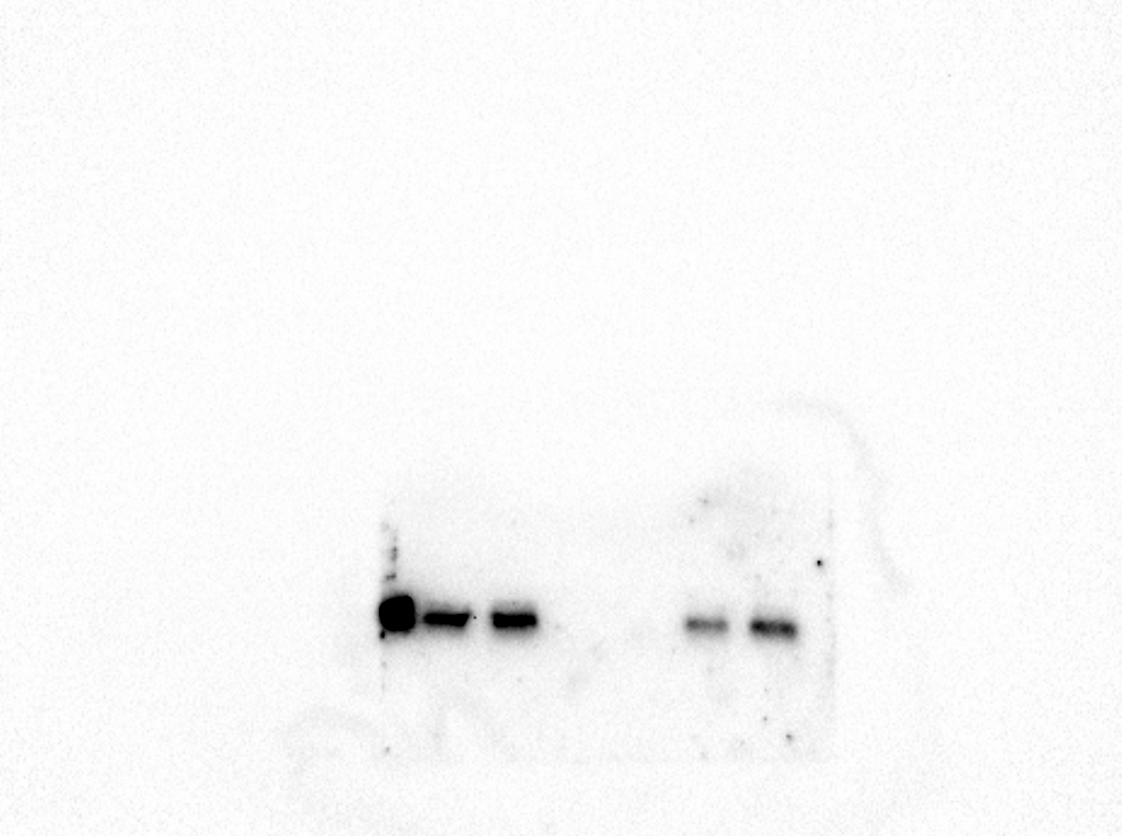

Supplement: Figure 6—figure supplement 1—source data 1. [file elife-82826-fig6-figsupp1-data1.zip › Figure 6-figure supplement 1-source data 1/Unlabeled Western blot/Fig 6-fig-sup 6A-TBP IP-BRF2 .tif]

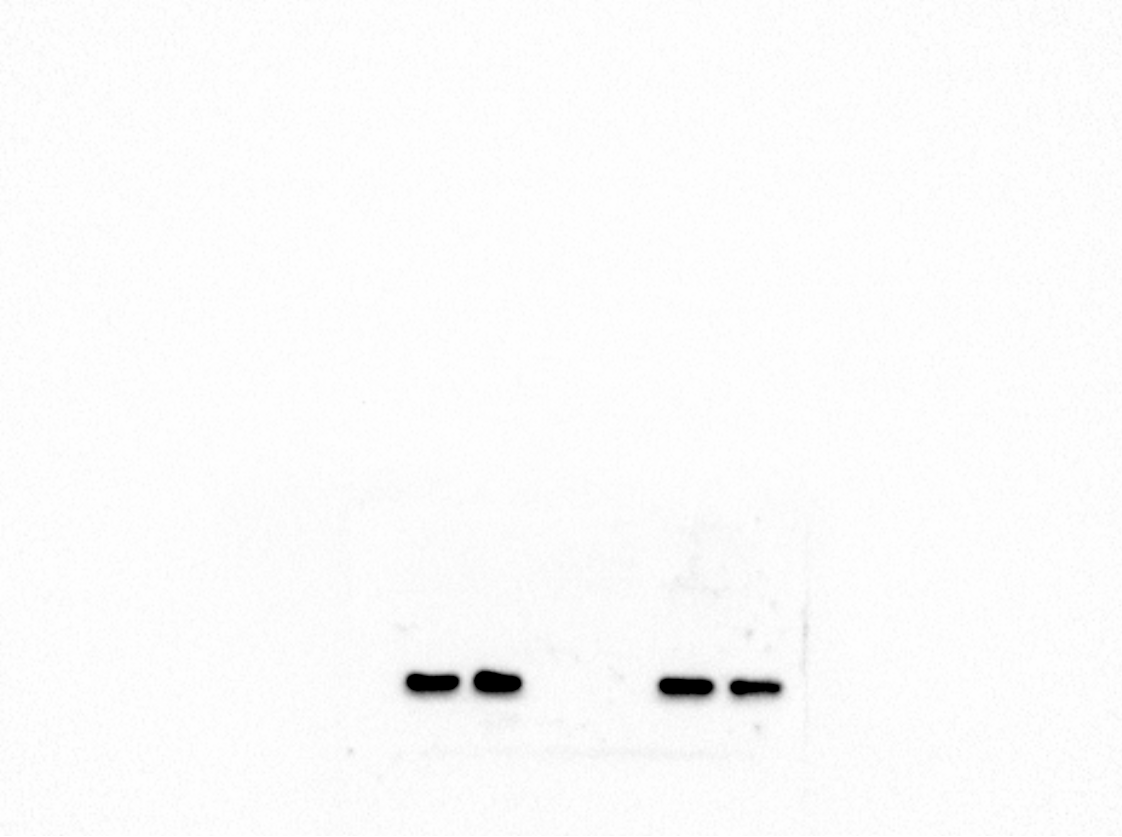

Supplement: Figure 6—figure supplement 1—source data 1. [file elife-82826-fig6-figsupp1-data1.zip › Figure 6-figure supplement 1-source data 1/Unlabeled Western blot/Fig 6-fig-sup 6A-TBP IP-TBP .tif]

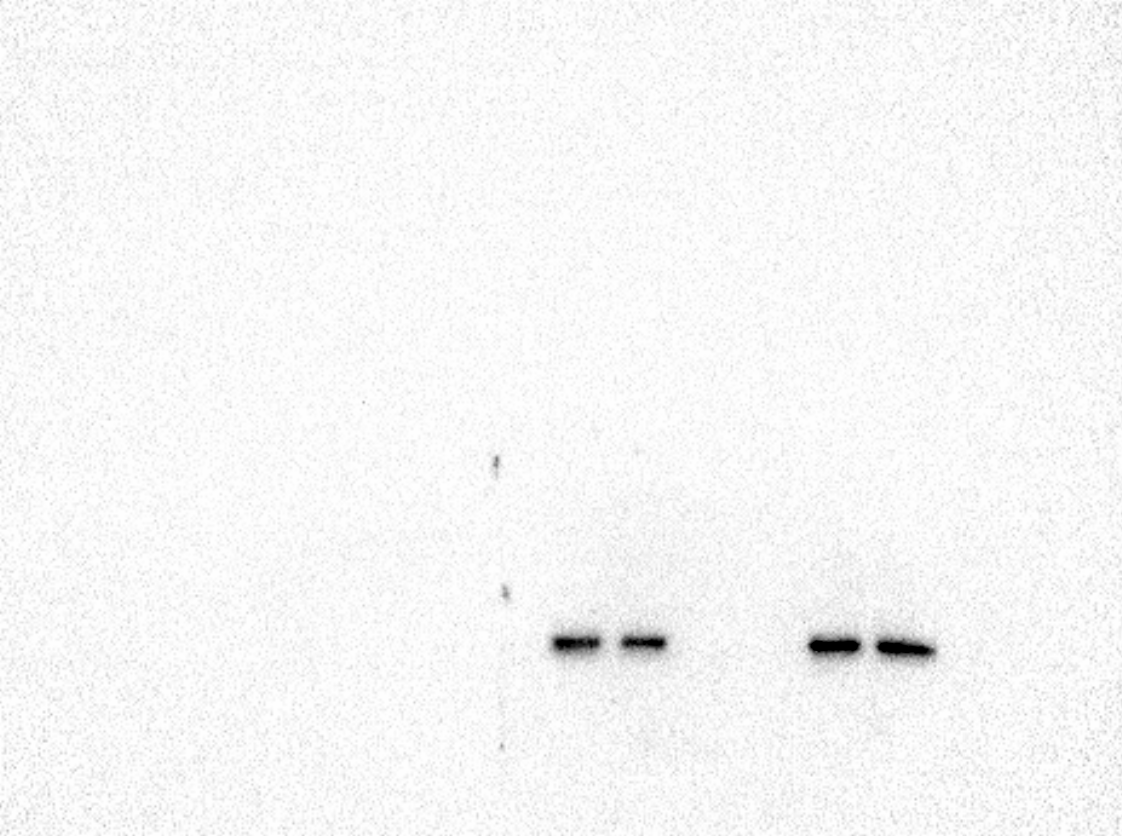

Supplement: Figure 6—figure supplement 1—source data 1. [file elife-82826-fig6-figsupp1-data1.zip › Figure 6-figure supplement 1-source data 1/Unlabeled Western blot/Fig 6-fig-sup 6B-BRF2 IP-BRF2 .tif]

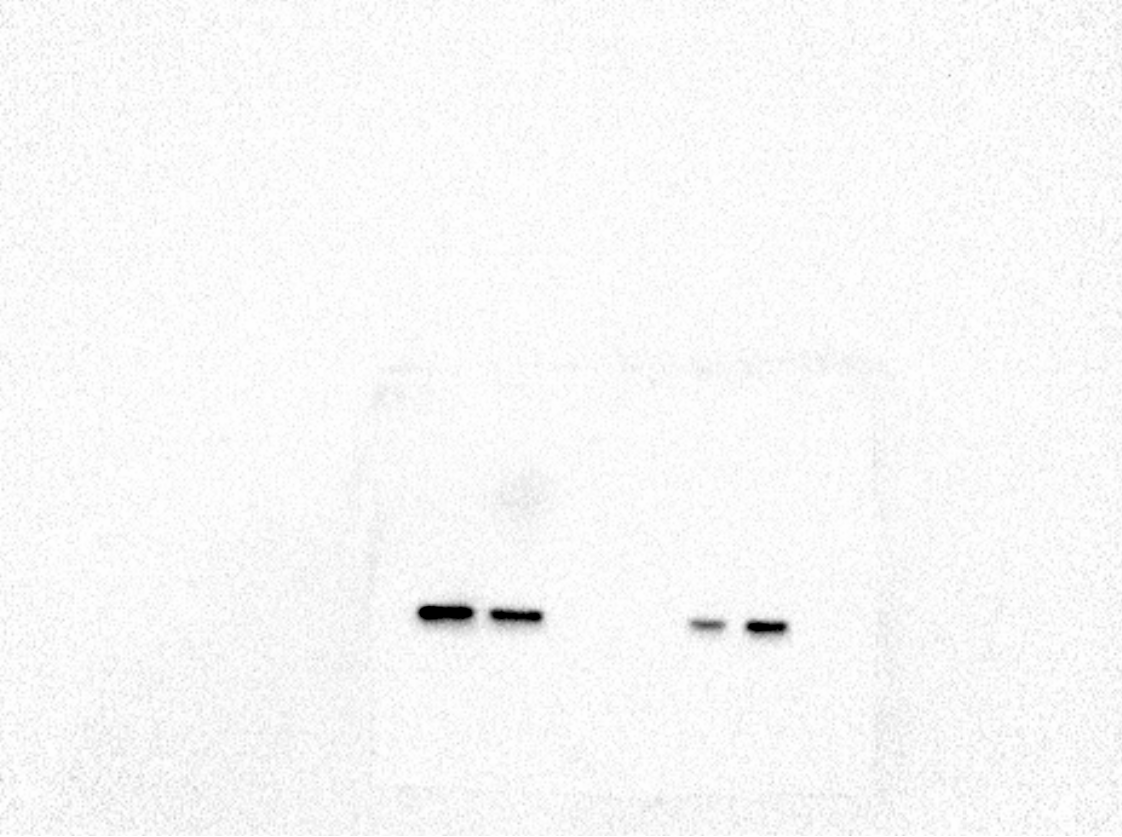

Supplement: Figure 6—figure supplement 1—source data 1. [file elife-82826-fig6-figsupp1-data1.zip › Figure 6-figure supplement 1-source data 1/Unlabeled Western blot/Fig 6-fig-sup 6B-BRF2 IP-TBP.tif]

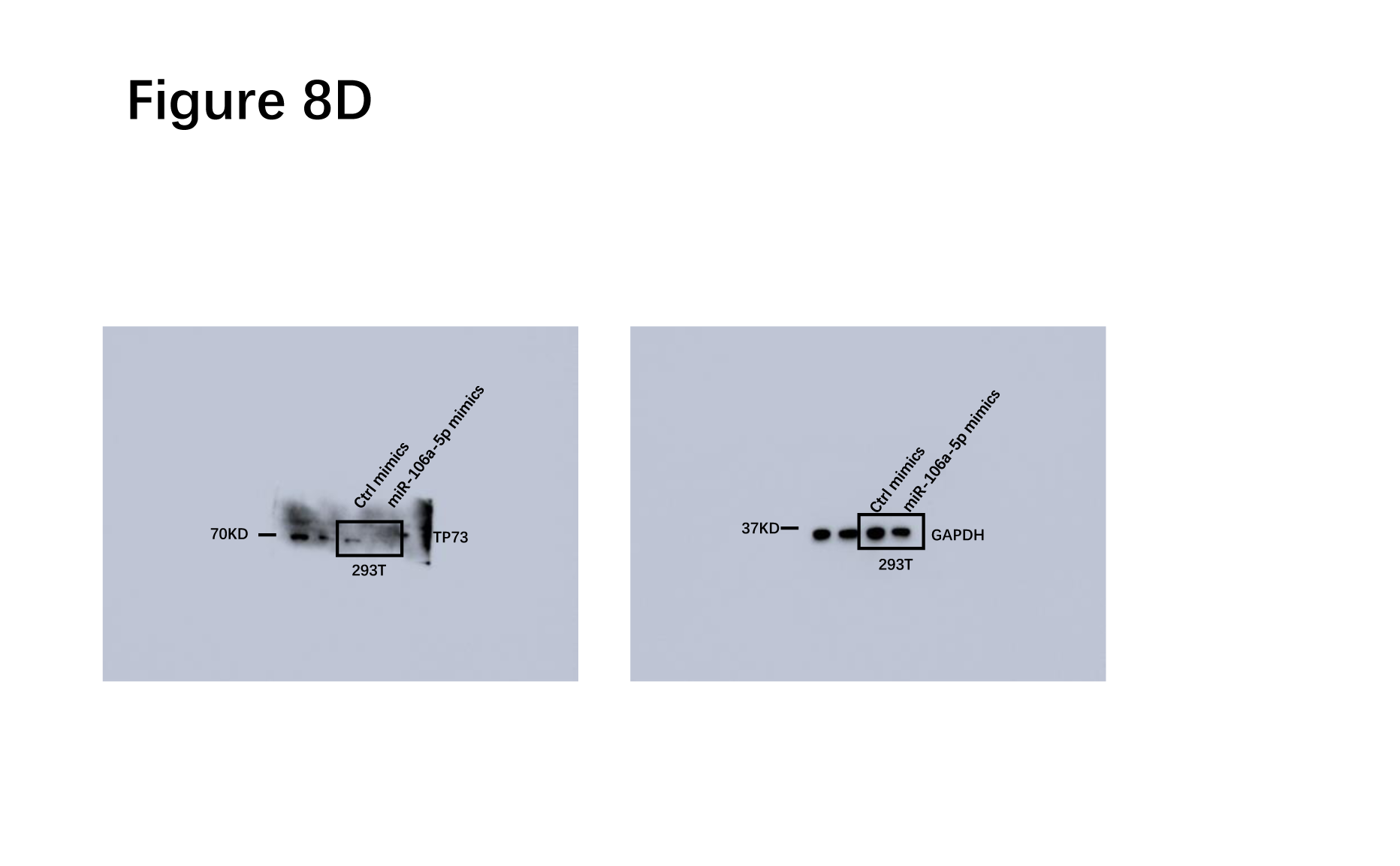

Supplement: Figure 8—source data 1. [file elife-82826-fig8-data1.zip › Figure 8 source data 1/Labeled Western blot/Figure 8D.tif]

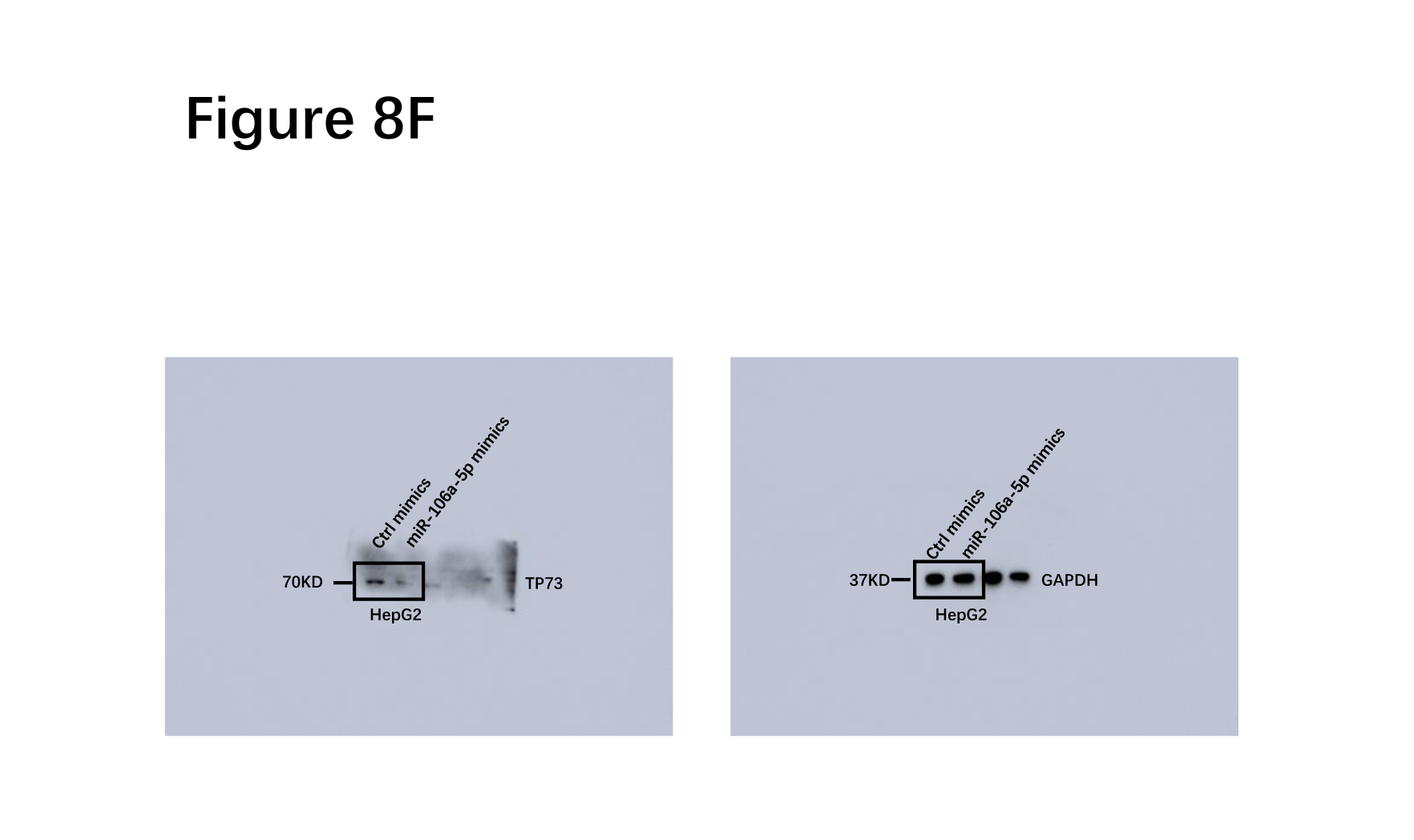

Supplement: Figure 8—source data 1. [file elife-82826-fig8-data1.zip › Figure 8 source data 1/Labeled Western blot/Figure 8F.tif]

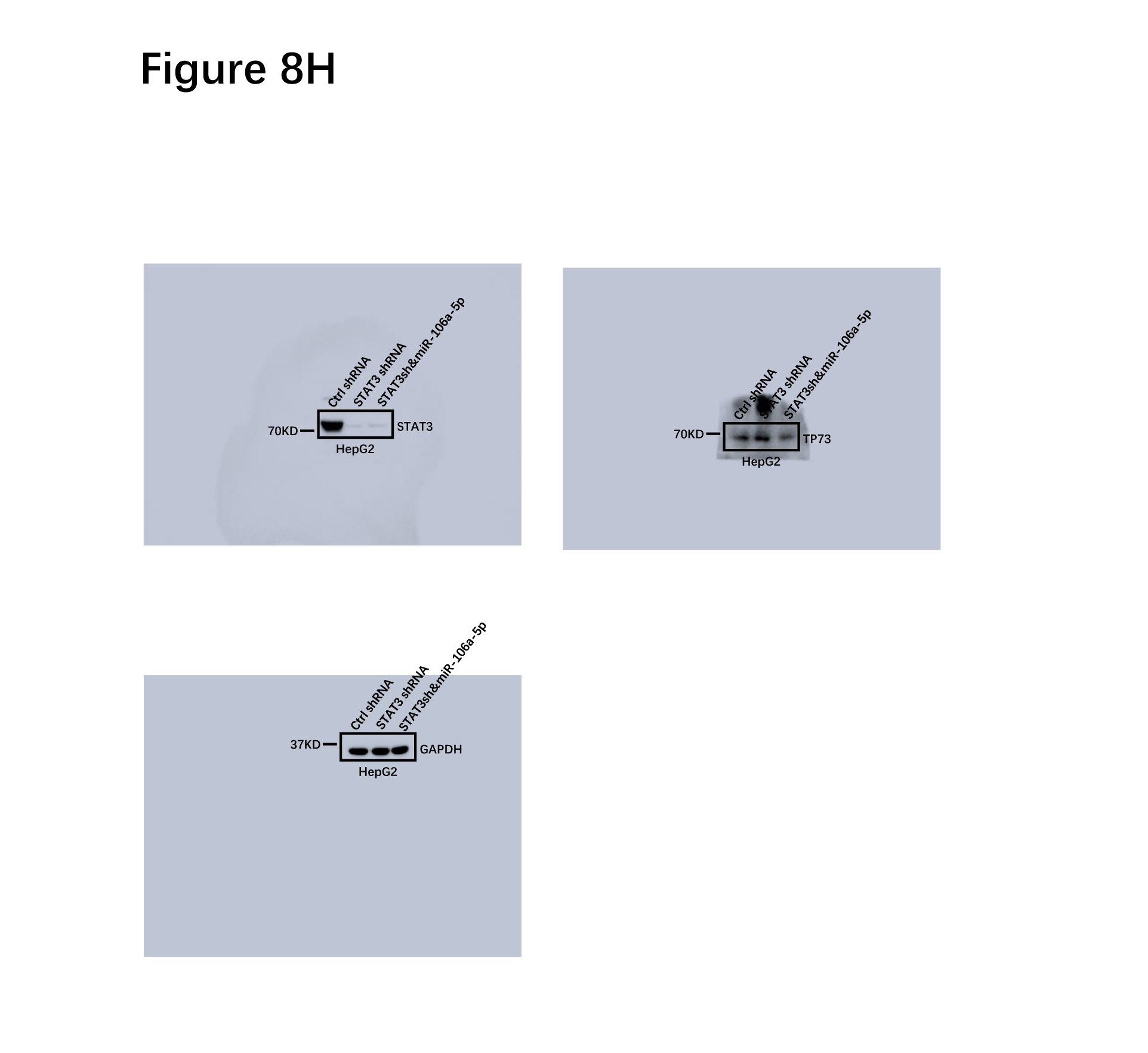

Supplement: Figure 8—source data 1. [file elife-82826-fig8-data1.zip › Figure 8 source data 1/Labeled Western blot/Figure 8H.tif]

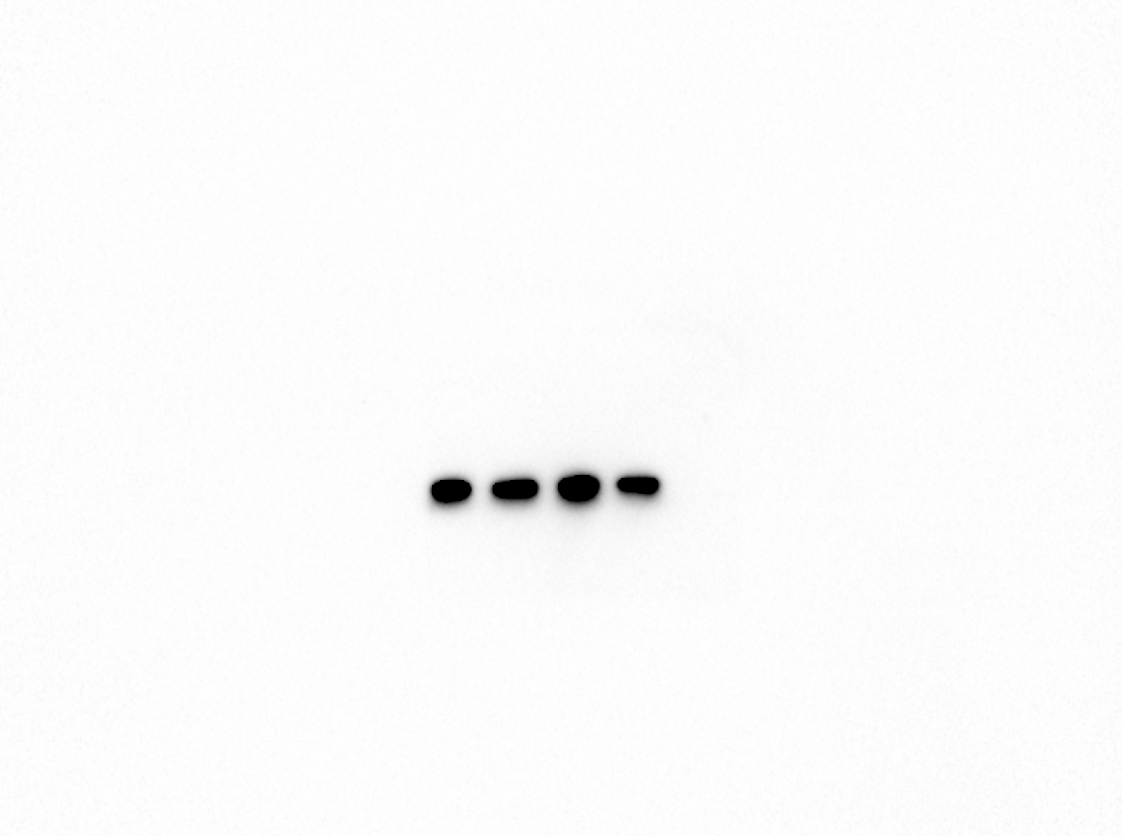

Supplement: Figure 8—source data 1. [file elife-82826-fig8-data1.zip › Figure 8 source data 1/Unlabeled Western blot/‏Figure 8D-GAPDH.tif]

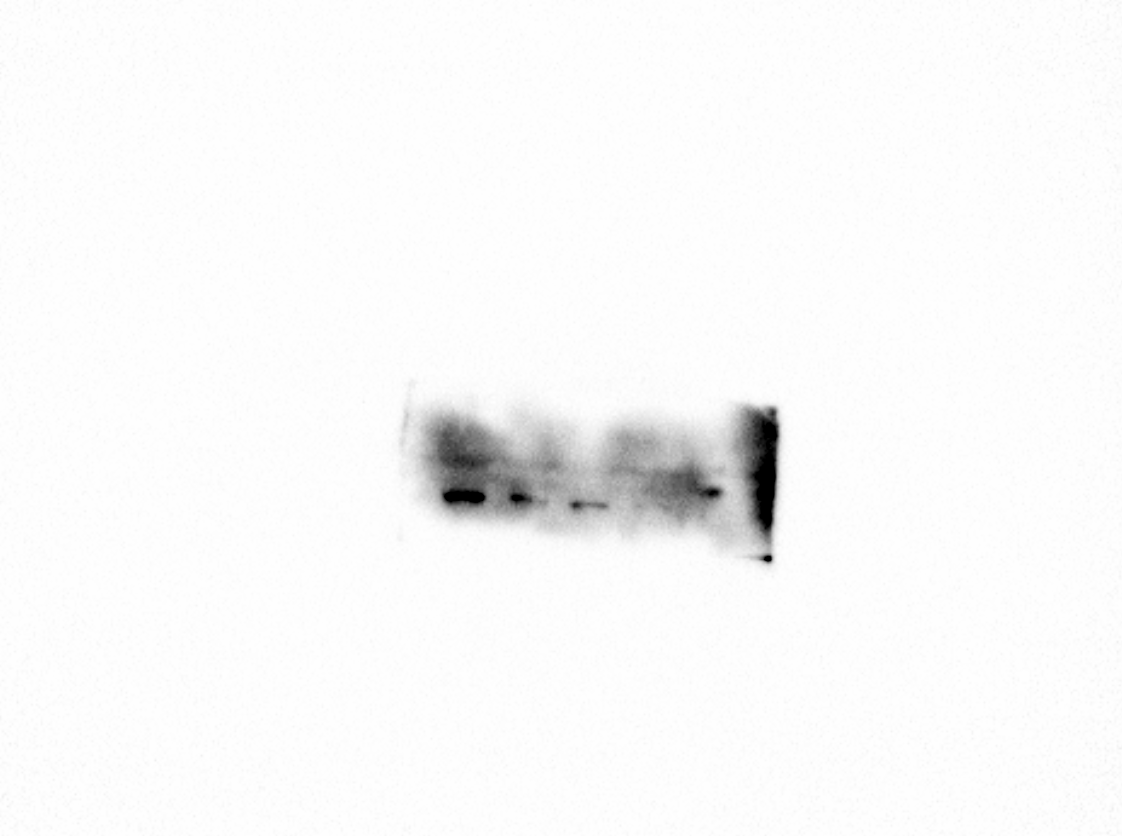

Supplement: Figure 8—source data 1. [file elife-82826-fig8-data1.zip › Figure 8 source data 1/Unlabeled Western blot/‏Figure 8D-TP73.tif]

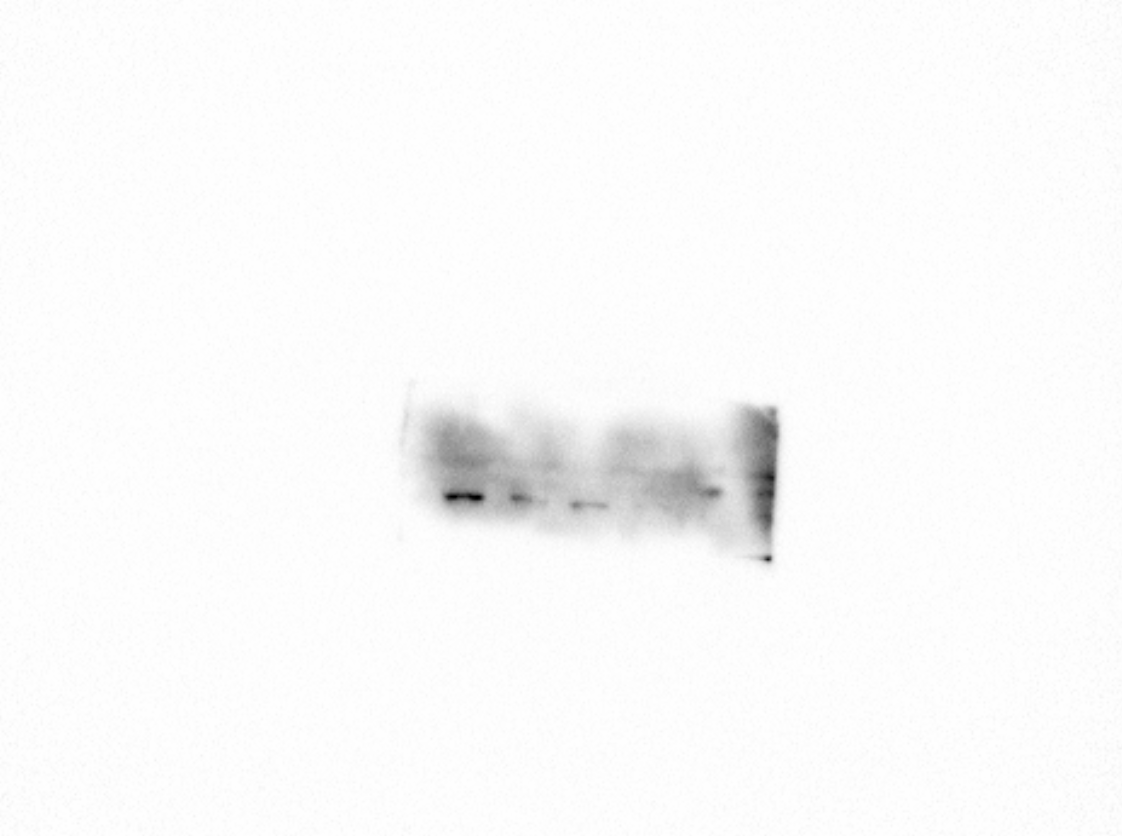

Supplement: Figure 8—source data 1. [file elife-82826-fig8-data1.zip › Figure 8 source data 1/Unlabeled Western blot/‏Figure 8F-TP73.tif]

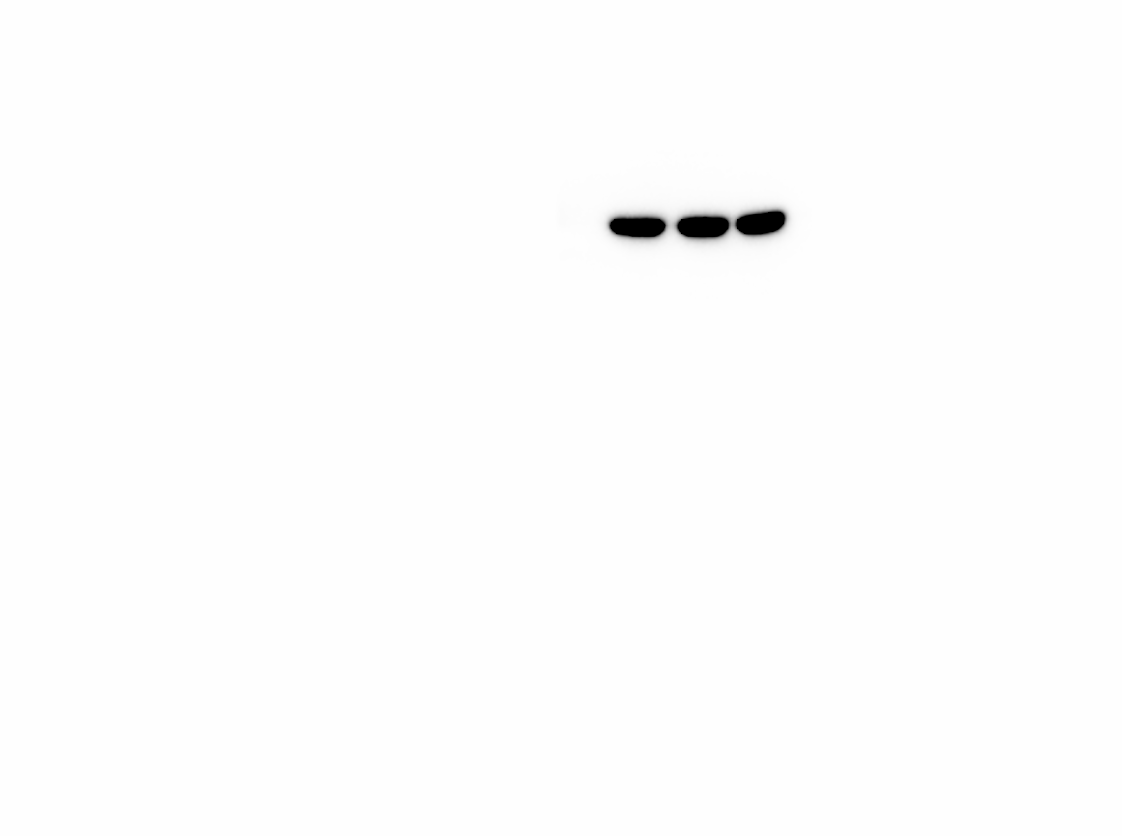

Supplement: Figure 8—source data 1. [file elife-82826-fig8-data1.zip › Figure 8 source data 1/Unlabeled Western blot/‏Figure 8H-GAPDH.tif]
