# Supplementary figures and images for: STAT3 promotes RNA polymerase III-directed transcription by controlling the miR-106a-5p/TP73 axis (part 2 of 2)
Source: eLife. 2023 Jan 19;12:e82826. doi: 10.7554/eLife.82826 (PMC9851613; doi:10.7554/eLife.82826)

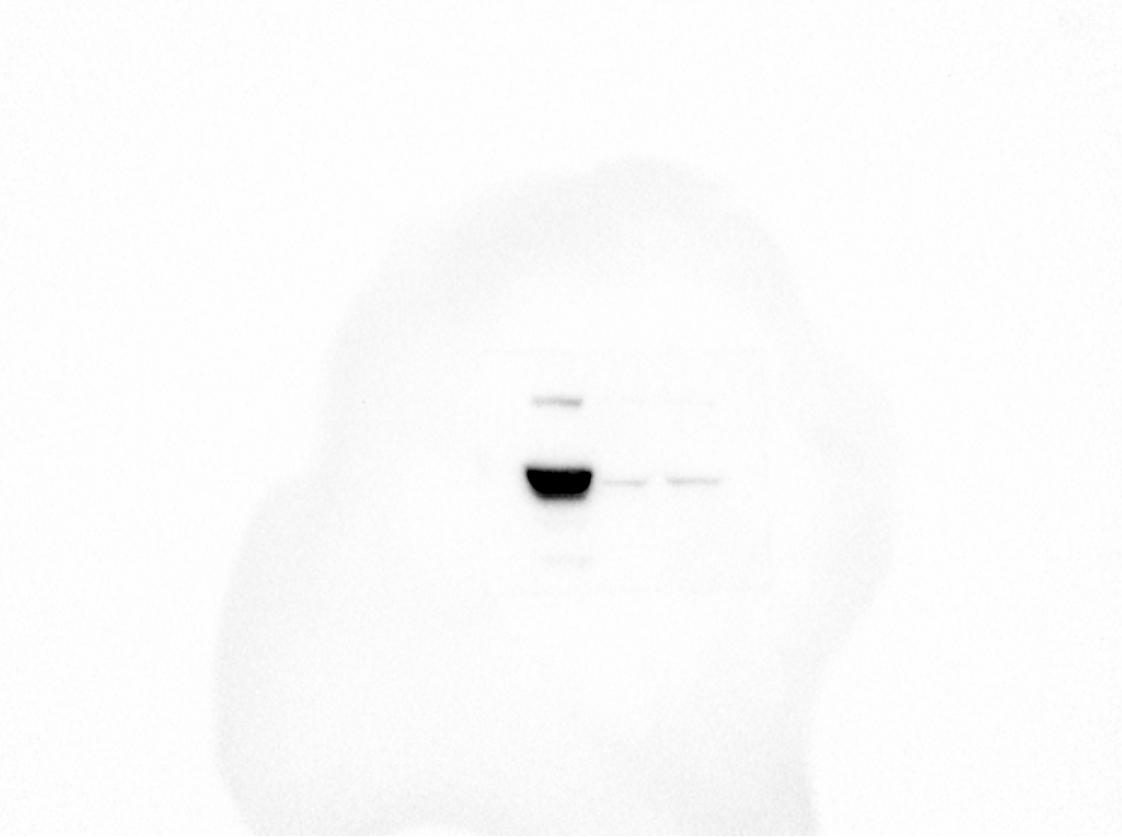

Supplement: Figure 8—source data 1. [file elife-82826-fig8-data1.zip › Figure 8 source data 1/Unlabeled Western blot/‏Figure 8H-STAT3.tif]

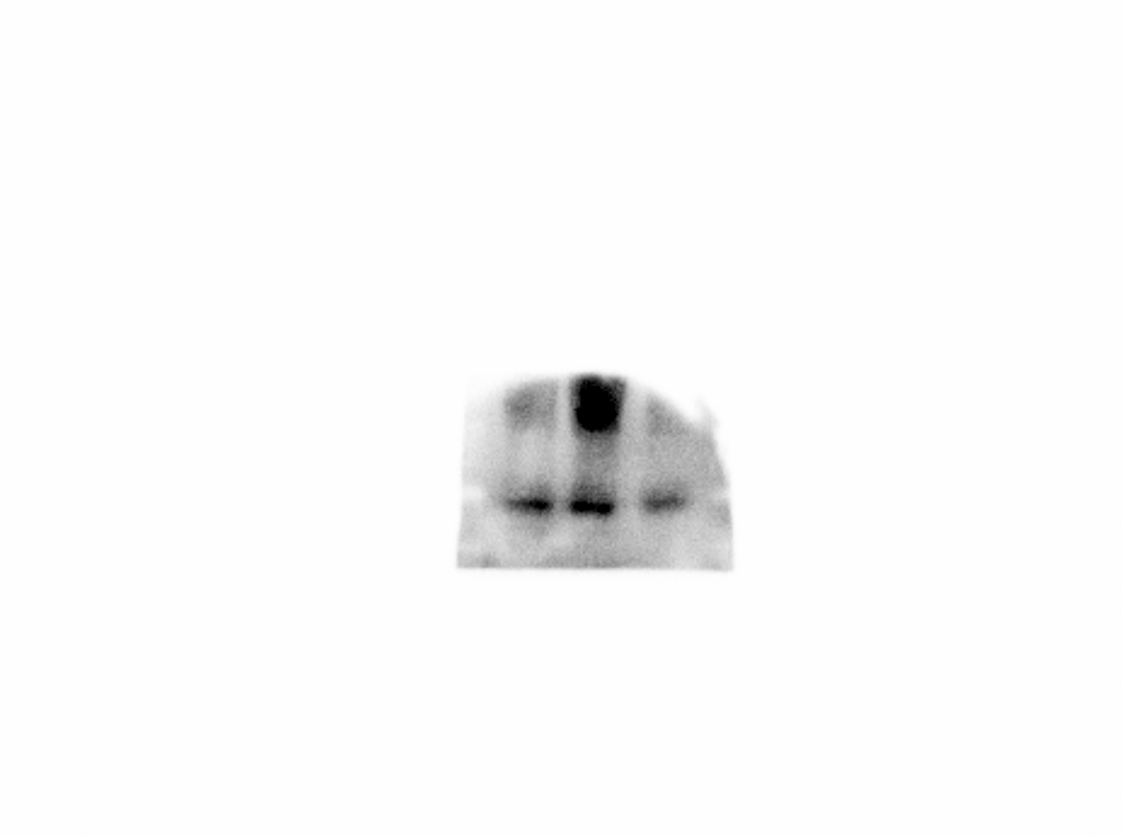

Supplement: Figure 8—source data 1. [file elife-82826-fig8-data1.zip › Figure 8 source data 1/Unlabeled Western blot/‏Figure 8H-TP73.tif]
